# Mitochondrial Transplantation: A Novel Therapeutic Approach for Treating Diseases

**DOI:** 10.1002/mco2.70253

**Published:** 2025-06-11

**Authors:** Xinglu Miao, Pei Jiang, Zhaoping Wang, Weihua Kong, Lei Feng

**Affiliations:** ^1^ Department of Neurosurgery Jining NO.1 People's Hospital Shandong First Medical University Jining China; ^2^ Medical Integration and Practice Center Cheeloo College of Medicine Shandong University Jinan China; ^3^ Institute of Central Nervous Vascular Injury and Repair Jining Academy of Medical Sciences Jining China; ^4^ Translational Pharmaceutical Laboratory Jining NO.1 People's Hospital Shandong First Medical University Jining China; ^5^ Institute of Translational Pharmacy Jining Medical Research Academy Jining China

**Keywords:** disease therapy, mitochondria, mitochondrial transplantation, therapeutic strategy

## Abstract

Advances in mitochondrial biology have led to the development of mitochondrial transplantation as a novel and promising therapeutic strategy. This review provides a comprehensive analysis of the multifaceted roles of mitochondria in health and disease, highlighting their central functions in energy production, antioxidant defense, calcium signaling, apoptosis regulation, and mitochondrial homeostasis maintenance. We explore the mechanisms by which transplanted mitochondria exert their therapeutic effects, including restoring ATP production, attenuating oxidative stress, modulating inflammatory responses, reducing cellular apoptosis, promoting cell repair and regeneration, facilitating neural circuit reconstruction, and exhibiting antitumor properties. Key preclinical studies demonstrating the efficacy of mitochondrial transplantation across in vitro and in vivo disease models are discussed, along with the status of clinical trials. The review also critically compares mitochondrial transplantation with other mitochondria‐targeted therapies, evaluating their relative advantages and limitations. Finally, we discuss the current challenges of translating this innovative therapy into clinical practice, such as mitochondrial isolation and purification, storage, targeted delivery, potential immune responses, and long‐term safety and efficacy concerns. This review aims to stimulate further research and development in this promising field, paving the way for novel therapeutic interventions for various diseases.

## Graphical abstract

This article describes the roles of mitochondria in health and disease and reviews the effects of mitochondrial transplantation in disease, including restoring ATP production, attenuating oxidative stress, modulating inflammatory responses, reducing apoptosis, promoting cell repair, and exhibiting antitumor properties. The article also reviews preclinical and clinical studies showing mitochondrial transplantation's efficacy in disease, and explores challenges in its clinical translation.

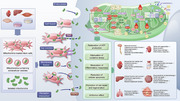

## 1 Introduction

Mitochondria are double‐membrane‐bound organelles widely recognized as the “powerhouses of the cell” due to their essential role in adenosine triphosphate (ATP) production through oxidative phosphorylation (OXPHOS) [1]. Since their discovery in the 19th century, extensive studies have revealed their multifaceted roles beyond energy production, including their involvement in calcium homeostasis, redox balance, and apoptotic signaling, which are critical mechanisms for cellular function and survival [2, 3, 4, 5]. Moreover, mitochondria possess their own deoxyribonucleic acid (DNA), enabling them to replicate independently and produce specific proteins essential for mitochondrial function [6]. Dysfunction in mitochondrial processes has been implicated in various diseases, including neurodegenerative, metabolic, and cardiovascular disorders, underscoring their significance in health and disease [7, 8].

In recent decades, advances in mitochondrial biology have provided novel insights into their roles in cellular homeostasis and pathophysiology. Emerging evidence highlights the centrality of mitochondria in complex cellular processes such as intracellular signaling, neurotransmitter synthesis, synaptic plasticity, and cellular repair [9, 10, 11]. However, mitochondrial dysfunction often leads to ATP depletion, oxidative stress, apoptosis, and inflammatory responses, which contribute to the pathogenesis of various diseases [12, 13]. Consequently, therapeutic strategies targeting mitochondria have garnered considerable attention, leading to the development of mitochondrial transplantation. This approach introduces exogenous healthy mitochondria into damaged cells or tissues, with the goal of restoring cellular energetics, reducing oxidative stress, and promoting tissue regeneration.

Mitochondrial transplantation represents a transformative frontier in disease therapy, distinguishing itself from conventional single‐target therapies by simultaneously addressing multiple pathological mechanisms. Preclinical studies in both in vitro and in vivo models have demonstrated promising results across a spectrum of diseases, including neurological disorders, cardiac injuries, and cancer [14, 15, 16]. Mechanistic studies suggest that transplanted mitochondria can restore ATP production, attenuate oxidative damage, reduce cellular apoptosis, and modulate inflammation, thereby facilitating cell repair and survival [14, 15, 16]. Furthermore, early‐stage clinical trials are beginning to explore the feasibility and safety of this innovative approach in humans. Despite its immense potential, several challenges remain, such as optimizing mitochondrial isolation, ensuring the functionality and integrity of transplanted mitochondria, and ensuring long‐term safety [17, 18, 19, 20].

This review aims to comprehensively examine mitochondrial transplantation as a novel therapeutic strategy. First, we discuss the critical roles of mitochondria in maintaining cellular health and their dysfunction in disease progression. Next, we explore the mechanisms by which mitochondrial transplantation exerts its therapeutic effects and summarize key preclinical and clinical studies supporting its efficacy. Additionally, we compare mitochondrial transplantation with other mitochondria‐targeted therapies, evaluating their respective advantages and limitations. Finally, we highlight the challenges and prospects of translating mitochondrial transplantation into clinical practice. By systematically analyzing current research and identifying existing gaps, this review seeks to stimulate further investigation and innovation in mitochondrial transplantation. It aims to provide researchers and clinicians with an integrated understanding of this promising therapeutic strategy and its potential applications in addressing a variety of complex and multifactorial diseases.

## 2 The Role of Mitochondria in Health and Disease

### 2.1 Energy Transduction and Provision

Mitochondria are critical organelles responsible for energy transduction and provision within eukaryotic cells. Often termed as the “powerhouses” of the cell, they are primarily involved in the production of ATP, which fuels various cellular processes. This energy transduction occurs through a sophisticated series of biochemical reactions known as OXPHOS.

OXPHOS occurs across the inner mitochondrial membrane (IMM) and involves the electron transport chain (ETC) and ATP synthase. The ETC consists of four multi‐subunit complexes (complexes I–IV) and two mobile electron carriers, ubiquinone and cytochrome *C* (Cyt *c*). Electrons derived from metabolic substrates such as nicotinamide adenine dinucleotide (NADH) and flavin adenine dinucleotide hydride (FADH2) are transferred through these complexes, driving protons across the IMM and creating proton motive force, an electrochemical gradient [21]. ATP synthase utilizes this proton motive force to catalyze ATP synthesis from adenosine diphosphate (ADP) and inorganic phosphate [21, 22]. This process is highly efficient and tightly regulated, ensuring that ATP production meets the cellular energy demands under varying physiological conditions.

In summary, during OXPHOS, mitochondria utilize NADH and FADH2 produced by the tricarboxylic acid (TCA) cycle in the mitochondrial matrix, to fuel the electron transport system located in IMM. This drives ADP conversion to ATP through OXPHOS, producing more than 90% brain ATP [23]. Neurons, compared with astrocytes, possess higher levels of active pyruvate dehydrogenase, exhibit faster TCA cycle activity, and prefer OXPHOS over aerobic glycolysis [24]. Conversely, astrocytes primarily satisfy their energy needs through aerobic glycolysis, resulting in fewer mitochondria in certain microdomains [25]. Consequently, neurons are especially susceptible to mitochondrial dysfunction and impaired mitochondrial transport to high energy‐demand regions such as growth cones [26, 27, 28].

Cardiomyocytes, which constitute approximately 80% cardiac cell volume, critically depend on mitochondrial OXPHOS for energy production. While OXPHOS normally produces manageable levels of reactive oxygen species (ROS) neutralized by endogenous antioxidant systems [29, 30], aging cardiomyocytes often exhibit compromised mitochondrial structural integrity and elevated ROS production, leading to progressive cellular dysfunction.

Mitochondria play a pivotal role in orchestrating immune responses through their energy‐generating capacity [31]. Immune cell activation necessitates substantial metabolic reprogramming to meet the increased energy demands associated with their effector functions, including inflammatory mediator synthesis and secretion and cellular migration to inflammation sites. To accommodate these intensive energy requirements, immune cells, particularly T and B cells, undergo metabolic adaptation by enhancing mitochondrial function [32, 33]. This increased mitochondrial function is a hallmark of activated T and B cells, as well as monocytes activated by other stimuli, such as TLR2 ligands, ensuring rapid ATP generation for immediate cellular demands [34].

### 2.2 Antioxidant Defense

Oxygen is crucial for the mitochondria during OXPHOS, meeting most cellular energy requirements [35]. A key element of OXPHOS is ETC, which comprises five protein complexes located in the IMM; generates ROS such as superoxide anion (O_2_^·−^), hydrogen peroxide (H_2_O_2_), and hydroxyl radicals (·OH) due to incomplete oxygen reduction; and is one of the primary ROS sources in cells.

During cellular respiration, electron leakage along the ETC forms mitochondrial ROS. Electrons from NADH or FADH2 are transferred to O_2_, generating O_2_^·−^, which is then converted to H_2_O_2_ [36]. Cellular ROS such as H_2_O_2_ and O_2_^·−^ undergo secondary reactions where O_2_^·−^ participates in iron‐catalyzed Fenton reactions yielding ·OH, or protonates to membrane‐damaging hydroperoxyl radicals [37] [38]. Superoxide dismutase (SOD) within mitochondria converts O_2_^·−^ into less reactive H_2_O_2_ [39] [40]. However, H_2_O_2_ generates ·OH via the Fenton reaction, damaging lipids, proteins, and nucleic acids [41].

ROS are regulated by various factors and enzymes and play critical physiological signaling roles, mediating essential cellular processes such as proliferation, differentiation, and migration [42, 43, 44]. Therefore, mitochondria exert regulatory control over metabolic processes and physiological functions at the cellular level through ROS production and redox‐dependent signaling pathways. Any perturbation in these signaling cascades may contribute to the pathogenesis of diverse pathological conditions.

Normally, cellular ROS concentrations are low; however, signaling specificity is lost when ROS levels exceed physiological thresholds, resulting in oxidative damage to macromolecules and cell death, termed “oxidative distress” [45]. Oxidative stress induces damage to macromolecules such as proteins and DNA, serving as a hallmark of age‐associated neurodegenerative disorders [46, 47]. ROS generation is pivotal in signaling processes that drive neuronal and axonal development and function [48, 49, 50, 51]. However, excess or incorrect ROS species can cause oxidative stress, disrupting the cytoskeleton and organelle functions, closely linked to neurodegeneration [52, 53]. Elevated levels of oxidative stress biomarkers are frequently observed in individuals with cardiovascular disorders, particularly those with hypertensive conditions and cardiac insufficiency [54, 55]. The accumulation of these stress indicators in cardiac muscle cells demonstrates a strong association with both the initiation and advancement of pathological cardiac remodeling [54, 56]. In the context of metabolic syndrome, which encompasses diabetic conditions, elevated arterial pressure, and adiposity, cardiac functional impairment primarily stems from intensified oxidative stress, which subsequently leads to mitochondrial damage, triggers death‐signaling cascades in mitochondria, and compromises the contractile capacity of cardiac myocytes [57]. The disruption of mitochondrial function, including a decline in membrane potential and excess ROS production, is an early trigger of kidney damage and a driving factor in chronic kidney disease initiation and progression [58]. Moreover, an intricate mechanistic interplay occurs between oxidative stress and inflammatory responses. The oxidative modification of biomolecules, proteins, and genetic material by mitochondria‐generated ROS initiates signaling pathways that promote inflammation, while the subsequent immune cell recruitment further perpetuates this cycle through localized ROS production, resulting in enhanced oxidative burden and tissue deterioration [59, 60].

Mitochondria depend on ROS clearance to protect the mitochondria ETC. SOD transforms superoxide into less reactive H_2_O_2_ [44]. Damage to mitochondria can enhance endogenous antioxidant systems, increasing SOD expression [61]. Catalase, glutathione peroxidase (GPx), and peroxiredoxin (Prx) enzymatically convert cellular H_2_O_2_ into H_2_O [62]. Mitochondria mainly rely on the combined actions of Prx, thioredoxins, and thioredoxin reductase 2 to decompose local H_2_O_2_^63^. Alternatively, GPx in the mitochondrial intermembrane space can convert H_2_O_2_ into water [63].

### 2.3 Calcium Homeostasis Maintenance

Mitochondria play a crucial role in maintaining cellular calcium homeostasis. They act as intracellular calcium ion (Ca^2+^) modulators and serve as high‐capacity Ca^2+^ storage systems. Mitochondria can uptake Ca^2+^ from the cytosol in a membrane potential‐dependent manner [64], with the mitochondrial matrix holding a negative charge of approximately −240 mV [65], facilitating the sequestration of positively charged Ca^2+^. The voltage‐dependent anion channel (VDAC) renders outer mitochondrial membrane (OMM) permeable to ions like Ca^2+^ [66], while IMM requires the specialized mitochondrial Ca^2+^ uniporter for Ca^2+^ passage [67, 68]. Mitochondrial Ca^2+^ sequestration prevents cytosolic Ca^2+^ overload and modulates feedback inhibition of Ca^2+^ transients [69, 70]. Mitochondria modulate intracellular Ca^2+^ levels through release and uptake mechanisms, thereby shaping cytoplasmic Ca^2+^ microdomains and influencing Ca^2+^ signal spread and frequency. This regulation is bidirectional, as mitochondrial Ca^2+^ uptake influences metabolic functions, including substrate uptake and mitochondrial dynamics [4, 5].

Ca^2+^ accumulation in mitochondria also modulates OXPHOS and energy production. This dual role as a Ca^2+^ buffer and cellular energy metabolism modulator is critical. Elevated respiratory rates necessitate substrate supply to mitochondria, with Ca^2+^ activating phosphorylase kinase via calmodulin, subsequently stimulating glycogen breakdown and increasing glucose availability. This pathway operates primarily in glycogen‐rich tissues, notably the muscle and liver [71], enhancing ATP supply [72, 73]. Ca^2+^ is also required for activating several metabolic enzymes, including pyruvate dehydrogenase, α‐ketoglutarate dehydrogenase, and isocitrate dehydrogenase. Ca^2+^ influx into the mitochondrial matrix enhances metabolism by interacting with these enzymes, thereby boosting ATP production [74]. The activity of pyruvate dehydrogenase is controlled via Ca^2+^‐dependent dephosphorylation, while calcium binding modulates the activities of α‐ketoglutarate dehydrogenase and isocitrate dehydrogenase, enhancing electron flow and boosting ATP production [75]. Neurons predominantly depend on OXPHOS for ATP generation and possess a limited ability to increase energy production through glycolysis when OXPHOS is impaired [76]. Consequently, mitochondria are essential targets for Ca^2+^ to ensure the activity‐dependent regulation of cellular energy metabolism.

Mitochondrial Ca^2+^ signaling extends beyond its role in OXPHOS regulation, significantly impacting cellular ROS production through two primary pathways: direct and indirect. Directly, Ca^2+^ activates ROS‐generating enzymes such as glycerol phosphate and α‐ketoglutarate dehydrogenase [77]. Indirectly, mitochondrial Ca^2+^ uptake slightly reduces the mitochondrial membrane potential, thereby contributing to ROS generation [78]. This intricate interplay between calcium signaling, ROS production, and cellular behavior highlights the complex role of mitochondrial Ca^2+^ in cellular function and response.

Mitochondrial Ca^2+^ is also vital for cellular processes, such as apoptosis and neurotransmitter release. Excess mitochondrial Ca^2+^ uptake can trigger mitochondrial permeability transition pore (MPTP) opening, initiating a cascade of events: mitochondrial swelling, membrane potential loss, and ATP synthesis disruption [78]. This sequence ultimately leads to apoptosis through a self‐reinforcing cycle [79]. Additionally, mitochondrial Ca^2+^ dynamics influence various cellular processes, including vesicular glutamate release from astrocytes, which in turn affects synaptic communication and neuronal excitability [80]. The importance of mitochondrial Ca^2+^ regulation is evident in neurological disorders. For instance, aberrant neuronal mitochondrial Ca^2+^ handling has been implicated in several genetic models of Parkinson's disease (PD) neurodegeneration [81, 82]. In Alzheimer's disease (AD), defective calcium handling exacerbates glutamate‐induced excitotoxicity, intracellular calcium accumulation, and ultimately, neuron loss [83]. In PD, upregulated mitochondrial calcium uptake leads to calcium overload within these organelles [84]. Similarly, in amyotrophic lateral sclerosis (ALS), elevated calcium flux coupled with impaired transfer from the endoplasmic reticulum to mitochondria contributes to neuronal calcium overload and subsequent damage [85]. The maintenance of proper mitochondrial Ca^2+^ flux, which governs cardiomyocyte contractile function, is critical since its perturbation (excess accumulation or depletion) serves as a pathogenic trigger in the onset and development of diverse cardiovascular disorders, such as cardiac ischemia–reperfusion (I/R) injury, cardiomyopathies, cardiac hypertrophy, and rhythm disturbances [86, 87]. Recent studies have revealed that mitochondrial Ca^2+^ homeostasis disruption contributes to tumor development, with numerous Ca^2+^‐regulating proteins being identified as oncogenes or tumor suppressors due to their critical roles in processes like cell proliferation and invasiveness [88, 89].

### 2.4 Mitochondrial Biogenesis

Upon receiving developmental cues and encountering environmental challenges, cells activate mitochondrial biogenesis, which is a highly complex and tightly regulated cellular process. This sophisticated machinery enables new mitochondrial unit proliferation through pre‐existing organelle replication, involving mitochondrial DNA (mtDNA) replication, transcription, and protein synthesis, as well as nuclear‐encoded protein import into mitochondria, ultimately ensuring functional mitochondria production [90, 91].

Mitochondrial biogenesis involves the growth and proliferation of existing mitochondria, ensuring a sufficient mitochondrial pool throughout cellular lifespan. This process is crucial for not only replenishing aging or damaged mitochondria, but also for meeting the dynamic energy demands of cells and compensating for any mitochondrial dysfunction [92, 93]. Notably, mitochondria cannot form de novo; hence, biogenesis relies on two primary mechanisms: incorporating newly synthesized proteins and lipids into pre‐existing mitochondria and the division of existing mitochondria through fission [91, 93].

Mitochondrial biogenesis can occur locally in distal axons of both the central and peripheral nervous systems [94]. This localized biogenesis is particularly upregulated in response to increased energetic demands, such as during axonal elongation and early disease response mechanisms [95]. Local mitochondrial biogenesis in axons is crucial for meeting the bioenergetic requirements of axonal growth, branching, and synaptic transmission. Emerging evidence suggests that mitochondrial biogenesis regulation represents a promising therapeutic avenue for various neurodegenerative disorders [96, 97]. This cellular process, when dysregulated, particularly contributes to the pathogenesis of several conditions including AD and PD [97, 98]. Research has demonstrated that enhancing mitochondrial generation and turnover could potentially ameliorate the progression of multiple neurological disorders, such as Huntington's disease (HD) and ALS [96]. The therapeutic potential of targeting mitochondrial renewal mechanisms has gained increasing attention, particularly owing to the observed deficits in this pathway among patients with neurodegenerative conditions.

Single‐cell transcriptional network analysis revealed that peroxisome proliferator‐activated receptor gamma coactivator 1‐alpha (PGC‐1α), a master orchestrator of mitochondrial biogenesis and metabolism, facilitates cardiomyocyte maturation via modulating YAP1 and SF3B2 signaling pathways [99]. In a diabetic myocardial model with hyperglycemic and hyperlipidemic conditions, adiponectin administration attenuates mitochondrial dysfunction through the activation of PGC‐1α‐dependent pathways [100]. The identification of this molecular mechanism elucidates a novel cardioprotective strategy and highlights its potential therapeutic implications in metabolic cardiac disorders. Furthermore, studies have demonstrated that compounds promoting mitochondrial biogenesis, notably alogliptin and the cyanobacterium *Spirulina platensis*, exhibit promising antidiabetic properties [101, 102].

While mitochondrial biogenesis predominantly occurs in healthy cells, cancer cells exhibit a notable correlation between enhanced OXPHOS, mitochondrial dynamics, and increased invasive and metastatic potential [103]. Targeted mitochondrial biogenesis therapies have demonstrated remarkable efficacy in managing relapsed and drug‐resistant malignancies [104]. In invasive cancers like osteosarcoma, aberrant mitochondrial dynamics can be effectively suppressed by 2‐methoxyestradiol through modulating key regulatory pathways including PGC‐1α, Cyt *c* oxidase I (COXI), and sirtuin 3 (SIRT3), thereby interrupting cancer stem cell propagation [105].

### 2.5 Mitochondrial Fission and Fusion

Mitochondrial dynamics, including fission and fusion processes, are vital for regulating mitochondrial morphology, size, distribution, and bioenergetic metabolism [106, 107]. Mitochondrial fission generates small, individualized organelles, which facilitates their distribution during cellular division and enables impaired mitochondrial unit isolation. In contrast, mitochondrial fusion generates elongated, reticular networks that optimize interorganelle communication and enable content exchange, thereby mitigating the accumulation of mtDNA mutations and oxidatively modified proteins.

Mitochondrial fission is a critical process in cellular dynamics, involving the division of a mitochondrion into two smaller units. This process is primarily mediated by the dynamin‐related protein 1 (DRP1) [108, 109], which interacts with mitochondrial adaptors including fission 1 protein (FIS1), mitochondrial fission factor, and 49 and 51 kDa mitochondrial dynamics proteins [110, 111]. Actin filaments and the endoplasmic reticulum also play supportive roles in this process [112]. Mitochondrial fusion is a complex process involving the merging of two mitochondria, typically through end‐to‐end collision. This mechanism is orchestrated by three proteins: Mitofusin (Mfn) 1 and Mfn2 facilitate OMM fusion, while optic atrophy 1 (OPA1) mediates IMM fusion [112, 113]. By facilitating content mixing between mitochondria, fusion serves multiple purposes: it alleviates stress on individual organelles, optimizes ATP production, and provides protection against mitophagy for stressed mitochondria [107, 114]. Working in concert with fission, these dynamic processes constitute an additional regulatory layer for maintaining the integrity and functionality of cellular mitochondria [115].

Mitochondrial dynamics, particularly the intricate interplay between fusion and fission mechanisms, emerge as crucial cellular homeostasis regulators [106]. These processes orchestrate mitochondrial morphological adaptations that influence vital cellular functions, including bioenergetic metabolism and programmed cell death pathways. Owing to their fundamental importance, perturbations in these dynamic processes have been implicated in the pathogenesis of various human pathologies, encompassing neoplastic diseases, metabolic regulation disorders, and neurodegenerative disorders [107]. Furthermore, the proteins mediating mitochondrial fission and fusion integrate multiple cellular signaling pathways to modulate mitochondrial shape and function in response to cellular needs [116]. The significance of these proteins extends beyond their role in mitochondrial dynamics, as mutations in their genes are associated with multiple disorders. For instance, *Mfn2* mutations are linked to Charcot‐Marie‐Tooth disease type 2A, a peripheral neuropathy [117]. *OPA1* mutations result in hereditary optic nerve degeneration and progressive blindness, while *DRP1* mutations have been implicated in abnormal brain development [118, 119].

While mitochondrial dynamics predominantly impact neuronal function, extensive experimental investigations have demonstrated that modulating mitochondrial dynamics, specifically the balance between fusion and fission processes, exerts profound effects on multiple aspects of tumor biology, including alterations in neoplastic metabolic reprogramming, cellular proliferative capacity, migratory potential, and cancer stem cell population maintenance within the tumor microenvironment [120]. The tumorigenic characteristics in pulmonary malignancies are correlated with enhanced mitochondrial fission, marked by elevated levels of both the native Drp1 protein and its hyperactive phosphorylated variant, Drp1–p616, suggesting a mechanistic link between mitochondrial fragmentation and cancer progression [121]. Subsequent experimental investigations have demonstrated that targeted suppression of Drp1‐mediated mitochondrial fission significantly attenuates the Ras‐driven xenograft tumor growth [122], highlighting the therapeutic potential of targeting mitochondrial dynamics in oncogenic Ras‐dependent malignancies.

### 2.6 Mitochondrial Quality Control

Defective mitochondria can result in a range of harmful effects, including calcium dysregulation, energy depletion, oxidative stress, and intrinsic apoptotic pathway activation [30, 123]. Mitochondrial quality control is crucial for preserving cellular function and maintaining cellular homeostasis by ensuring damaged or dysfunctional organelle removal, thereby preventing the accumulation that could compromise cellular health and viability. Three major pathways contribute to mitochondrial quality control, each targeting different levels of mitochondrial damage. Misfolded proteins within the mitochondrial matrix and inner membrane are degraded by ATP‐dependent mitochondrial AAA+ proteases [124]. These proteases, including i‐AAA and m‐AAA, reside on opposite sides of the MIM and selectively recognize and degrade misfolded proteins, ensuring mitochondrial proteome integrity. Mitochondrial‐derived vesicles serve as a mechanism for removing oxidized or damaged proteins and lipids, as well as facilitating assembled mitochondrial protein complex turnover [125]. This process involves vesicle budding from mitochondria [126]. These mitochondrial‐derived vesicles are subsequently targeted to lysosomes for degradation, providing a selective means of eliminating damaged components without compromising the entire organelle. Mitophagy, a specialized form of autophagy, targets entire damaged mitochondria or segregated subdomains for degradation. This process involves the sequestration of the damaged mitochondrion within autophagosomes, which then merge with lysosomes to create autolysosomes [127]. Mitophagy represents the most comprehensive quality control mechanism, capable of eliminating severely damaged or dysfunctional mitochondria to maintain overall cellular health.

A central mechanism governing mitophagy hinges on the interplay between PTEN‐induced kinase 1 (PINK1) and the cytosolic E3 ubiquitin ligase Parkin [128, 129], both genetically implicated in familial PD [130, 131]. Upon mitochondrial depolarization, PINK1 evades its usual proteolytic degradation and accumulates on OMM, which triggers Parkin recruitment to the OMM, initiating a cascade of phosphorylation and ubiquitination events targeting OMM proteins. This cascade ultimately flags the damaged mitochondria for degradation by recruiting the autophagosomal membrane and its associated machinery [132, 133]. Mitophagy, a crucial process in maintaining mitochondrial homeostasis and cellular signaling, involves the selective autophagic removal of mitochondria. While facilitated by mitochondrial fission and fragmentation, mitophagy is primarily triggered by mitochondrial dysfunction and operates through pathways distinct from general autophagy [134]. This process responds dynamically to various physiological signals, including oxygen deprivation, oocyte fertilization, and stem cell property maintenance [135, 136, 137].

The significance of mitophagy in human health has been highlighted by the discovery that mutations in genes governing this process are associated with various pathological conditions, particularly neurodegenerative disorders, such as PD, AD, and HD [138, 139, 140]. Furthermore, such mutations have also been documented in cancer [141], suggesting that impaired mitophagic processes play a crucial role in disease pathogenesis. In patients with chronic obstructive pulmonary disease (COPD), impaired mitophagy mechanisms result in cellular accumulation of dysfunctional mitochondria. These compromised organelles generate elevated ROS levels, subsequently initiating inflammatory cascades that contribute to COPD pathogenesis. Studies have demonstrated that *PRKN* (the gene encoding Parkin protein) expression, a critical mediator of mitophagic processes, is significantly diminished in pulmonary epithelial cells of affected individuals compared with that in healthy individuals [142]. This reduction in PRKN levels potentially explains the observed mitophagic dysfunction, ultimately fostering an environment of enhanced oxidative stress through the retention of malfunctioning mitochondria. The cardiac tissue exhibits vulnerability to oxidative damage, with sustained exposure to ROS contributing to multiple pathological conditions, including enhanced susceptibility to I/R injury, cardiac dysfunction, and degenerative process progression [143]. Notably, ROS serve as crucial signals for initiating mitochondrial autophagy, representing a protective mechanism for eliminating compromised organelles [144]. Research has demonstrated that dysregulating this mitochondrial quality control pathway correlates with various cardiac pathologies [1], emphasizing the critical role of efficient mitophagy in preserving cardiomyocyte function and survival.

### 2.7 Apoptosis

Mitochondria, essential for cellular energy production and metabolism, have evolved sophisticated mechanisms to eliminate dysfunctional cells through various effectors that trigger cell death when vital functions are compromised. One such mechanism is apoptosis, a tightly regulated process crucial for both development and adult tissue homeostasis [145]. Apoptosis is characterized by distinct morphological changes and proceeds through a series of sequential steps [2]. Two primary signaling pathways govern apoptosis: the extrinsic and intrinsic pathways. The intrinsic pathway, often termed the mitochondrial pathway, highlights mitochondrial critical role in integrating pro‐ and antiapoptotic signals to initiate cell death [146, 147]. In this regulated process, mitochondria release specific proteins that activate intrinsic cell death programs, effectively orchestrating final moments of the cells [148, 149]. This mitochondrial involvement underscores the significance of mitochondria beyond energy production, emphasizing its role in cellular life and death decisions.

The intrinsic mitochondrial pathway of apoptosis is mainly controlled by the B‐cell lymphoma 2 (Bcl‐2) protein family, which includes both antiapoptotic proteins like Bcl‐2 and Bcl‐xL, and proapoptotic proteins like Bcl‐2 associated X protein (Bax) and Bcl‐2 homologous antagonist/killer (Bak) [150]. The delicate balance between these proteins determines cell fate. This pathway is triggered by Bcl‐2 homology region 3 (BH3)‐only proteins, which are Bcl‐2 family members [151]. When apoptotic signals are received, BH3‐only proteins are activated, derepressing Bax and Bak, which are crucial for inducing mitochondrial outer membrane permeabilization (MOMP), a key event in the apoptotic process. MOMP results in the release of various proapoptotic factors, most notably Cyt *c*, from the mitochondrial intermembrane space. Once in the cytosol, Cyt *c* interacts with apoptotic peptidase‐activating factor 1 and procaspase‐9 to form the apoptosome [152, 153, 154]. This complex activates caspase‐9, which in turn activates the executioner caspases 3, 6, and 7 [145]. These caspases proteolytically break down various cellular substrates, causing the typical morphological and biochemical changes of apoptosis [145].

Another important player in mitochondrial‐mediated cell death is MPTP, a multiprotein complex spanning both mitochondrial membranes, composed of various components including the adenine nucleotide translocase, VDAC, cyclophilin D, and potentially the ATP synthase [155, 156, 157]. MPTP opening can lead to mitochondrial permeability transition (MPT), resulting in mitochondrial swelling, rupture, and proapoptotic factor release [158]. MPT can be triggered by various cellular stressors, including calcium overload and oxidative stress [158]. High Ca^2+^ influx activates the mitochondrial MPTP, creating a feed‐forward cycle that leads to MPT, amplifies mitochondrial dysfunction, and ultimately leads to cell death [78, 79]. Unlike MOMP‐induced apoptosis, which is typically caspase‐dependent, MPT‐induced cell death can occur independently of caspase activation [159]. Recent research has implicated the ATP synthase as a potential component of the MPTP [155, 156, 157], suggesting its dual role in both energy production and cell death regulation. This finding highlights the intricate connections between cellular metabolism and apoptosis.

Apoptosis serves as a fundamental homeostatic mechanism essential for cellular turnover throughout the lifespan of multicellular organisms. However, its dysregulation can precipitate or result from various pathological conditions, including neurodegenerative disorders [160]. In neuronal cells, the apoptotic machinery extends beyond cellular death, playing a crucial role in axonal degeneration. This process can be triggered by multiple factors, such as neurotrophic support withdrawal, excitotoxicity, or the progression of neurodegenerative disorders like PD [161, 162]. Understanding these mechanisms is vital for developing targeted therapies for neurological disorders.

The precise regulation of apoptosis is crucial for maintaining cellular homeostasis. However, any disruption to this delicate equilibrium can precipitate various pathological conditions. On one hand, insufficient apoptotic activity may facilitate cancer development and progression [163]. On the other hand, excess apoptotic processes are neurodegenerative disorders, including PD, AD, and HD [164]. Furthermore, aberrant apoptotic regulation is a significant factor in the development of autoimmune disorders and certain infectious diseases [165, 166]. Investigations have uncovered the pivotal function of cellular apoptotic mechanisms in ischemic heart disease. Particularly, programmed cell death mechanisms play a fundamental role in cardiomyocyte destruction during acute myocardial infarction (AMI), with the most pronounced effects manifesting in regions bordering the infarcted tissue [167, 168]. Moreover, clinical data have revealed that individuals who experience early‐onset symptomatic ventricular dysfunction following AMI demonstrate notably elevated rates of apoptotic activity [169].

## 3 The Role of Mitochondrial Transplantation in Disorders

**FIGURE 1:**
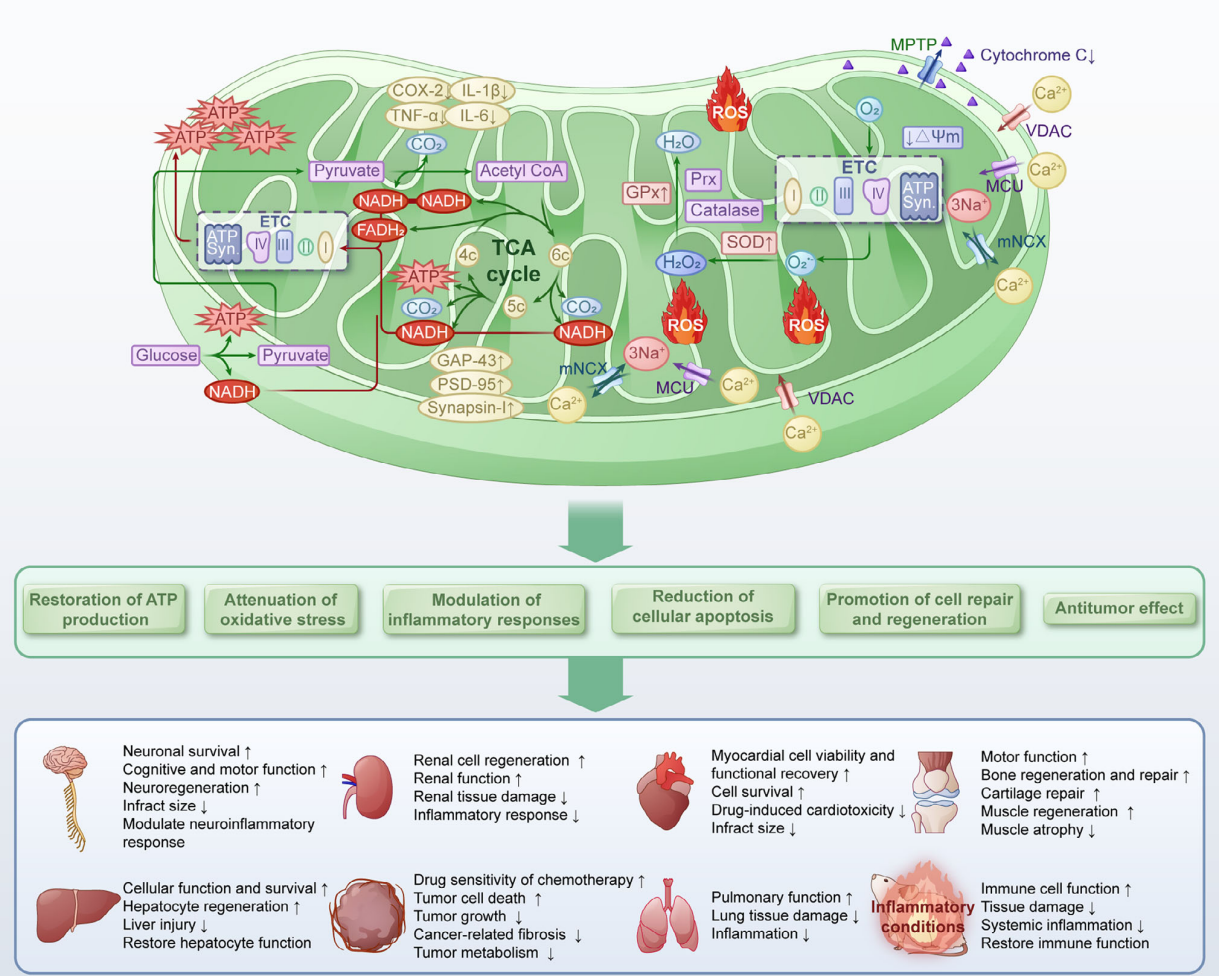
Roles of mitochondrial transplantation in disorders.

Mitochondrial transplantation is an emerging therapeutic strategy that involves transplanting exogenous healthy mitochondria into diseased or injured areas through various methods. This approach aims to improve and restore recipient cell dysfunction, enhance cell survival, and thereby assist in the self‐repair of cells, tissues, and organs, ultimately ameliorating or treating diseases [170]. Mitochondrial transplantation is based on the ability of exogenous mitochondria to be internalized by recipient cells and integrate into the existing mitochondrial network. This process improves mitochondrial function, increases ATP production, reduces oxidative stress, and enhances cellular survival [170, 171]. Here, we summarize the roles and mechanisms of mitochondrial transplantation in addressing disorders and explores its potential clinical applications (Figure 1).

### 3.1 Restoration of ATP Production

Mitochondrial transplantation can significantly enhance the ATP production capacity of damaged tissues. When healthy mitochondria are transplanted into damaged tissue, they can rapidly integrate into the recipient cell's mitochondrial network. This integration not only increases the number of functional mitochondria within the cell, but also enhances overall mitochondrial function [171]. The integration process involves mitochondrial fusion with the recipient cell and mtDNA and protein exchange, thereby boosting the cellular energy metabolism capacity [170].

Transplanted mitochondria increase ATP production through OXPHOS, which is the primary source of energy metabolism in cells [3, 172, 173]. OXPHOS occurs in IMM, where the energy from nutrients is converted into ATP via ETC. The increased ATP production provides the necessary energy support for various cellular repair processes, including protein synthesis, cell membrane repair, and ion pump function [174]. Incubating the culture medium of human neuroblastoma SH‐SY5Y cells with exogenous mitochondria for 24 h concentration‐ and time‐dependently increase cell viability as well as significantly increase cellular ATP content for at least 3 days [175]. These findings suggest that supplementation with functional exogenous mitochondria enhances cell growth and proliferation, potentially through increased ATP production. Furthermore, intravenous mitochondrial injection into mice significantly increase ATP levels in the brain, heart, liver, muscle, and kidney for at least 3 days [175].

Restoring ATP levels is crucial for maintaining normal neuronal function after neural injury. Adequate ATP supply supports neurotransmitter release and reuptake, maintains membrane potential, and supports axonal transport [176]. Wang et al. [177] first demonstrated that mitochondrial transplantation restores ATP production dysfunction in a lipopolysaccharide (LPS)‐induced mouse model of depression, significantly increasing ATP levels in the hippocampus. These processes are essential for neuron survival and functional recovery. ATP levels in damaged neural tissue can significantly increase within a short period after mitochondrial transplantation, sometimes even returning to near‐normal levels [178]. This rapid improvement in energy status can prevent further cell death and create favorable conditions for subsequent repair processes [179]. Traumatic brain injury (TBI) leads to rapid mitochondrial dysfunction and significant ATP level reduction in the damaged cortex of mice. Mitochondrial transplantation effectively restores ATP levels and mitochondrial complex activity in the injured cortex [180]. Related studies have shown significantly reduced ATP content in the ischemic penumbra of a middle cerebral artery occlusion model in rats [181]. However, ATP levels in the ischemic penumbra rapidly increase after mitochondrial transplantation [181]. Supplementation with exogenous mitochondria can provide additional energy for the early repair of ischemia‐damaged brain tissue. Further studies have corroborated that healthy mitochondria transplantation restores mitochondrial respiration, ATP levels, and mitochondrial membrane potential in ischemic brain tissue.

In addition, multiple studies have shown that mitochondrial transplantation therapy can be employed to restore ATP production under various pathological conditions. Through innovative mechanisms of mitochondrial transfer, it exhibits significant potential in addressing cellular energy metabolism disorders. In carbon tetrachloride (CCl4)‐induced liver injury models, mitochondrial transplantation has shown remarkable efficacy in ATP restoration. Research revealed that mitochondria enter the hepatocytes via macropinocytosis, recovering cell viability concentration‐dependently and significantly increasing ATP supply [182]. The mechanism involves triggering mitochondrial unfolded protein response, activating respiratory chain enzyme genes, and ultimately rebalancing cellular homeostasis. Acute lung injury models have similarly demonstrated extraordinary ATP restoration capabilities. Studies showed significant mitochondrial transfer to alveolar epithelia, resulting in increased alveolar ATP concentrations and protection against LPS‐induced injury [174]. Exosomes from adipose‐derived mesenchymal stem cells (ASCs) further enhances ATP production in alveolar macrophages [183]. Similarly, in calvarial defect models, mitochondrial transplantation enhances ATP production, promoting bone defect repair [184]. The consistent theme across these studies is mitochondrial transplantation's ability to modulate cellular energy metabolism, suggesting a versatile therapeutic approach for restoring ATP production in various pathological conditions. In summary, mitochondrial transplantation provides critical energy support for cellular repair and functional recovery of damaged tissue by restoring and enhancing ATP production, opening new possibilities for treating diseases and injuries.

### 3.2 Attenuation of Oxidative Stress

Mitochondrial transplantation can effectively reduce oxidative stress in damaged tissue through multiple mechanisms. Healthy transplanted mitochondria can improve the overall mitochondrial function of damaged cells, thereby enhancing the antioxidant capacity [170].

Oxidative stress is a major problem following tissue damage, which can cause further cell damage and death [52, 185]. Transplanted mitochondria can reduce ROS production by improving ETC efficiency [186]. Studies have shown that mitochondrial transplantation can significantly reduce ROS levels in neuronal cells, thereby decreasing oxidative stress [172, 175]. In a mouse model of respiratory chain inhibitor MPTP‐induced PD, mitochondrial transplantation improved motor function and Parkinsonian behavioral symptoms by increasing ETC activity, reducing ROS levels, and preventing cellular apoptosis and necrosis, thereby halting experimental PD progression [175]. Localized mitochondrial injection into the distal segment of injured sciatic nerves in rats, followed by further evaluation, demonstrated that mitochondrial transplantation alleviated oxidative stress in the injured nerves and improved nerve conduction electrophysiology and muscle activity [172]. Notably, mitochondrial transplantation significantly enhanced hepatocyte viability, reduced ROS‐induced damage, and improved liver function in the CCl4‐induced liver injury mouse model. This therapy restored mitochondrial function through the upregulation of antioxidant genes to scavenge free radicals and transcriptional activation of respiratory chain enzymes and mitochondrial‐associated genes, ultimately enhancing cellular resistance to stress [182].

Furthermore, healthy mitochondria can help restore cellular oxidative–reductive balance. They carry their own antioxidant systems, including SOD and GPx, which can directly scavenge free radicals [39, 40, 62, 177]. After transplantation, these additional antioxidant defense mechanisms can enhance the ability of damaged cells to resist oxidative damage. A study on the therapeutic effects of mitochondrial transplantation in rats with traumatic spinal cord injury revealed that it significantly reduces inducible nitric oxide synthase, nitric oxide, and 3‐nitrotyrosine levels at the injury site, indicating oxidative stress reduction and neuronal damage mitigation [187]. Moreover, a study investigating the antidepressant effects of intravenously injected isolated mitochondria in a LPS‐induced mouse model of depression found that mitochondrial transplantation reduces oxidative stress, evidenced by decreased ROS and malondialdehyde levels as well as increased SOD activity [177]. Additionally, in a streptozotocin (STZ)‐induced rat model of diabetic nephropathy, mitochondrial transplantation reduced oxidative stress and promoted cellular repair through mitochondrial SOD2 and antiapoptotic pathway regulation [188].

Mitochondrial transplantation can also indirectly reduce oxidative stress by improving calcium homeostasis. Calcium overload is an important factor leading to mitochondrial dysfunction and increased ROS production [189]. Experimental observations have shown that Ca^2+^ can reduce ROS leakage from mitochondrial respiratory chain complexes I and III under physiological condition; however, Ca^2+^ can enhance ROS production under pathological conditions [190]. Excess Ca^2+^ accumulation in the mitochondrial matrix triggers MPTP formation, leading to the uncontrolled apoptotic factors and ROS release [190]. Healthy transplanted mitochondria can help regulate intracellular calcium levels, thereby reducing calcium imbalance‐induced oxidative stress [191, 192]. Notably, mitochondrial transplantation can not only reduce existing oxidative stress, but also enhance cellular resistance to future oxidative damage through upregulation antioxidant gene expression and extending antioxidant protection [178, 193].

In conclusion, mitochondrial transplantation provides multifaceted protective mechanisms to alleviate oxidative stress in damaged tissue by reducing ROS production, enhancing antioxidant defenses, and improving calcium homeostasis. This comprehensive antioxidant effect is of great significance for promoting cellular survival and functional recovery.

### 3.3 Modulation of Inflammatory Responses

Inflammation is a common feature of tissue damage and diseases, and while moderate inflammation can be beneficial for tissue repair, excess or prolonged inflammation can lead to secondary damage and functional impairment [194]. Mitochondrial transplantation demonstrates significant potential in regulating inflammatory responses across various biological systems.

Mitochondrial transplantation modulates the inflammatory response through multiple mechanisms: First, mitochondrial transplantation suppresses proinflammatory cytokine production. Introducing healthy mitochondria can significantly decrease the expression levels of proinflammatory factors such as interleukin‐6 (IL‐6), tumor necrosis factor‐α (TNF‐α), and cyclooxygenase‐2 (COX‐2) in damaged cells [177, 195]. Research indicates that mitochondrial transplantation significantly attenuates inflammatory responses in a LPS‐induced mouse model of depression, specifically by reducing the expression of proinflammatory cytokines such as IL‐1β, TNF‐α, and COX‐2 in the hippocampus [177]. In a rat model of spinal cord I/R injury characterized by an inflammatory response with IL‐6 and TNF‐α activation and subsequent mitochondrial dysfunction in the spinal cord, exogenous viable mitochondrial transplantation during the reperfusion phase significantly attenuates local proinflammatory responses (IL‐6 and TNF‐α), and the improved mitochondrial function and reduced inflammation collectively contributed to the inhibition of spinal cord cell apoptosis [187]. Emerging evidence indicates that mitochondrial transplantation suppresses proinflammatory cytokine production (e.g., TNF‐α, IL‐1β, IL‐6) in the spinal cord, with inflammation being a critical factor in neuropathic pain development; mitochondrial transplantation significantly alleviates mechanical and thermal hyperalgesia in spinal nerve ligation rats, indicating potent neuroprotective effects [196].

Second, mitochondrial transplantation can reduce oxidative stress‐induced inflammation. Healthy mitochondria can enhance cellular antioxidant capacity and reduce the production of ROS [197]. ROS can trigger and exacerbate inflammatory responses through multiple mechanisms, including the activation of inflammatory signaling pathways and the promotion of proinflammatory cytokine release [198]. ROS are key triggers of inflammation, which in turn exacerbates oxidative stress, creating a vicious cycle that leads to cellular damage and disease progression [198, 199]. Since ROS are important inflammatory triggers, lowering ROS levels can effectively inhibit the initiation of inflammatory cascades.

Another important mechanism is the effect of mitochondrial transplantation on macrophages and glial cells. Administering exosome derived from human ASCs (AdMSC‐Exos) in an acute LPS‐induced lung injury mouse model via tail vein injection shifts macrophages toward an anti‐inflammatory phenotype, characterized by reduced proinflammatory cytokine secretion and enhanced anti‐inflammatory cytokine production [183]. Similarly, in an in vitro diabetic nephropathy model, mitochondrial transplantation promotes lysosome‐autophagy processes for mitochondrial quality control, facilitating the polarization of macrophages to an anti‐inflammatory phenotype. These findings suggest a potential role of mitochondrial transplantation in mitigating inflammation and alleviating tissue damage in diabetic nephropathy [200]. Additionally, in an in vitro model of simulated inflammation, mitochondrial transplantation effectively suppresses the production of proinflammatory cytokines in macrophages and reduces inflammation through inhibition of the nuclear factor kappa‐B (NF‐κB) signaling pathway [201]. Collectively, these studies demonstrate that mitochondrial transplantation holds therapeutic potential for shifting macrophages to an anti‐inflammatory phenotype. Neuroinflammation is an immune response in the central nervous system (CNS) activated by microglia and astrocytes. Astrocytes and microglial cells play a crucial role in neuroinflammation, and their functional state is closely related to mitochondrial health [202, 203]. Mitochondrial transplantation can modulate their inflammatory response, promoting neuroprotection and repair. Mitochondrial transplantation drives microglial cells toward an anti‐inflammatory M2 phenotype, reducing the production of proinflammatory cytokines and providing neuroprotection [202]. Mitochondrial transplantation also inhibits the proliferation of microglia and astrocytes in the hippocampus of with status epilepticus mice models, which represents a significant neuroprotective effect since glial cell activation is associated with neuroinflammation and subsequent neuronal damage [203]. Additionally, mitochondrial transplantation significantly reduces neuroinflammatory responses after ischemic brain injury, as evidenced by decreased expression of glial fibrillary acidic protein and reduced reactive gliosis in the penumbra region [181]. Recent studies have shown that mitochondrial transplantation alleviates neuroinflammation in a TBI rat model by inhibiting the activation of astrocytes and microglia, and thereby improving sensorimotor function in the TBI rat model [204].

In conclusion, mitochondrial transplantation comprehensively modulates the inflammatory response through multiple mechanisms, including reducing proinflammatory factor production, alleviating oxidative stress, and influencing macrophages and glial cell function. This integrated effect can not only mitigate acute inflammatory damage, but also improve chronic inflammatory conditions, providing new strategies for disease treatment.

### 3.4 Reduction of Cellular Apoptosis

Apoptosis is a key pathological process in many diseases and injuries, and mitochondria play a central role in regulating apoptosis [147, 148]. Mitochondrial transplantation reduces apoptosis through multiple mechanisms. First, mitochondrial transplantation can improve energy metabolism and reduce apoptosis induced by ATP deficiency. Healthy mitochondria can enhance the cellular ATP production capacity, meeting the energy needs of the cell and preventing apoptosis due to energy depletion [175, 205]. The energy status of a cell is crucial for maintaining its normal function. A significant decline in intracellular ATP levels triggers a cascade of stress responses, including MPTP opening and Cyt *c* release, both of which are critical in apoptosis [206, 207]. Studies have shown that mitochondrial transplantation significantly improves neuron survival rates in ischemic brain injury models [178]. In the PD model, intravenous mitochondrial administration significantly improves behavioral outcomes, restores mitochondrial function including mitochondrial complex I activity and ATP content, and ameliorates apoptosis in the striatum [175].

Second, mitochondrial transplantation can reduce oxidative stress‐induced apoptosis. Oxidative stress is a major cause of apoptosis in many diseases [208]. By providing functional mitochondria, transplantation can enhance cellular antioxidant capacity, reduce ROS production, and protect cells from oxidative damage [209]. Research has demonstrated that introducing mitochondria into 6‐hydroxydopamine‐induced pheochromocytoma (PC12) cells and PD rat models reduces ROS levels, decreases apoptosis, promotes cell survival under oxidative stress, improves motor function, and mitigates the loss of dopaminergic neurons in the substantia nigra pars compacta [210]. Additionally, mitochondrial transplantation significantly reduces oxidative stress and apoptosis after ischemic brain injury by decreasing malondialdehyde and 8‐hydroxy‐2′‐deoxyguanosine levels, while increasing the activities of SOD and GPx [181].

Additionally, mitochondrial transplantation can regulate the expression and activity of apoptosis‐related proteins. Healthy mitochondria can maintain the balance of Bcl‐2 family proteins, increasing the expression of antiapoptotic proteins, while inhibiting the activity of proapoptotic proteins [150]. Regulating this balance can effectively prevent Cyt *c* release and caspase cascade activation [153, 211]. Platelet‐derived mitochondrial transplantation reduces mitochondrial dysfunction and neuronal apoptosis in an in vitro hypoxia/reoxygenation (H/R) model, as evidenced by decreased mitochondrial Cyt *c* release and reduced expression of the proapoptotic protein Bcl‐2‐interacting mediator of cell death [212]. Exogenous mitochondrial transplantation significantly reduces apoptosis levels in Neuro‐2a (N2a) cells under H/R injury, as evidenced by the downregulation of proapoptotic proteins, including a decreased Bax/Bcl‐2 ratio and reduced caspase‐3 expression [213]. A study on mitochondrial transplantation for treating traumatic spinal cord injury in rats demonstrated that mitochondrial transplantation promotes antiapoptotic proteins like Bcl‐2 and reduces proapoptotic markers like cleaved caspase‐3 and Bax [187]. This suggests that mitochondrial transplantation helps reduce cell death in the injured spinal cord.

Mitochondrial transplantation can also reduce apoptosis by maintaining mitochondrial membrane potential. Introducing healthy mitochondria can help maintain normal membrane potential, preventing the opening of MPTP, thereby stopping the initiation of the apoptotic process [123]. The opening of MPTP depolarizes the mitochondrial membrane potential, releases apoptogenic factors, and disrupts OXPHOS. In certain apoptotic systems, mitochondrial membrane potential loss can be an early event in the apoptosis process [214]. Current evidence suggests that the intranasal administration of exogenous mitochondria restores ATP production and improves mitochondrial membrane potential in the medial prefrontal cortex of mice with ischemic stroke [215]. In studies on mitochondrial transplantation for H/R injury, mitochondrial transplantation significantly improved H/R‐induced reductions in mitochondrial membrane potential, excess ROS generation, and Cyt *c* release, thereby reducing H/R‐induced apoptosis and enhancing cell survival [212]. Injecting healthy mitochondria into the prefrontal cortex of adolescent rats with schizophrenia (SZ) can prevent the dissipation of mitochondrial membrane potential and attention deficits in adulthood [216]. Additionally, in vitro experiments demonstrate that transferring isolated, functionally normal mitochondria into SZ‐derived lymphoblasts leads to long‐term improvements in multiple mitochondrial functions, such as cellular oxygen consumption and mitochondrial membrane potential [216].

Mitochondrial transplantation has represented a novel intervention to mitigate cellular apoptosis and restore tissue function across various pathological conditions. In a rabbit ischemic heart model and a piglet model of right heart failure, mitochondrial transplantation restored ATP synthesis, reduced ROS levels, stabilized the mitochondrial membranes, and suppressed caspase‐3 and caspase‐9 activities [217, 218]. These effects significantly decrease cardiomyocyte apoptosis and preserved myocardial function. Similarly, mitochondrial transplantation restored mitochondrial membrane potential, inhibited Cyt *c* release, enhanced renal tubular regeneration, and consequently reduced apoptosis and improved kidney function in acute kidney injury caused by I/R injury [219]. Furthermore, in a cecal slurry‐induced sepsis model, mitochondrial transplantation restored mitochondrial function in immune cells, reduced ROS production, and attenuated TUNEL‐positive apoptotic signals in the spleen [220]. In sepsis‐associated acute lung injury, mitochondrial dysfunction in pulmonary microvascular endothelial cells exacerbates endothelial barrier dysfunction and pulmonary edema. Mitochondrial transplantation from mesenchymal stem cells (MSCs), mediated via tunneling nanotubes (TNT), effectively restored mitochondrial function by reducing ROS, enhancing ATP production, and suppressing caspase‐dependent apoptosis, thereby alleviating lung injury and preserving vascular integrity [221].

In summary, mitochondrial transplantation reduces apoptosis through multiple mechanisms, including improving energy metabolism, reducing oxidative stress, modulating apoptosis‐related proteins, and maintaining mitochondrial membrane potential. This comprehensive effect not only shields cells from diverse damaging factors but also offers novel potential strategies for treating chronic diseases and acute injuries.

### 3.5 Promotion of Cell Repair and Regeneration

Mitochondrial transplantation has shown remarkable potential in promoting cell repair and regeneration across various pathological conditions. In I/R‐induced kidney injury, mitochondrial transplantation effectively reduced mitochondrial ROS production, protecting renal cells from oxidative damage and fostering tubular cell regeneration [219]. Similarly, in diabetic nephropathy, mitochondrial transplantation restored renal proximal tubular epithelial cells by alleviating oxidative damage and reinstating cellular transport mechanisms [188]. Furthermore, in nephrotoxicity models, MSC‐derived mitochondrial transplantation significantly reduced oxidative stress and apoptosis while promoting tubular cell regeneration [222]. The therapeutic benefits of mitochondrial transplantation extend beyond renal repair, offering significant potential in muscle injuries. Mitochondrial transplantation notably increased ATP production, a critical component of cellular repair processes such as protein synthesis and cytoskeletal reconstruction. In the dexamethasone‐induced muscle atrophy model, mitochondrial transplantation increased the expression of OXPHOS complex enzymes, particularly complex I, thereby enhancing ATP production in the damaged muscle. Additionally, mitochondrial transplantation promoted mitochondrial regeneration and functional recovery by upregulating mitochondrial biogenesis‐related protein PGC‐1α, a key marker of mitochondrial biosynthesis, ultimately facilitating muscle repair and regeneration [223]. It also significantly reduces the deposition of noncontractile tissue and collagen in injured muscles by downregulating the expression of transforming growth factor beta‐1 and collagen types III and IV [224]. This mechanism modulates extracellular matrix composition, alleviates fibrosis, and thereby supports muscle tissue regeneration. In bone defect models, mitochondrial transplantation demonstrated significant regenerative potential by transferring healthy mitochondria to MSCs, which improved their proliferation, migration, and osteogenic differentiation while enhancing OXPHOS and ATP production to accelerate bone defect healing [184, 225].

Axon regeneration is a crucial process for neural repair and essential for restoring neuronal function. However, axon regeneration is often limited following nervous system injury, partly due to an unfavorable microenvironment and decreased intrinsic regenerative capacity of neurons. Mitochondrial transplantation shows significant potential in promoting axon regeneration in neurons. Supplementing damaged neurons with healthy mitochondria can promote neurite regeneration and enhance neuronal viability [226]. Previous studies show that mitochondrial transplantation therapy promotes axonal and neurite regrowth in injured hippocampal neurons [227]. These findings suggest that mitochondrial transplantation holds potential therapeutic prospects for promoting regeneration in the injured CNS. A study on the neuroprotective effects of mitochondrial transplantation in optic nerve injury demonstrated that transplanted active mitochondria could integrate into the retina and significantly improve retinal oxidative metabolism within one day. This improvement was evidenced by an increased spare respiratory capacity in the retinal mitochondria after mitochondrial transplantation, indicating enhanced mitochondrial quality [228]. Mitochondrial transplantation significantly increased the survival rate of retinal ganglion cells and promoted axonal extension 28 days after optic nerve injury, suggesting its potential in promoting axonal regeneration [228]. Mitochondrial transplantation can significantly improve energy supply to neurons. Axon growth is a highly energy‐demanding process, requiring substantial ATP to support cytoskeletal remodeling, membrane synthesis, and protein transport [170]. Studies have shown that transplanting healthy mitochondria can increase ATP production in damaged neurons, providing necessary energy support for axon regeneration [171, 229]. Cerebral ischemia deprives neural cells of their energy supply, often resulting in axonal degeneration and demyelination in the adjacent white matter. In focal ischemic murine models with transplanted mitochondria, higher levels of myelin basic protein and more morphologically intact myelinated axons were observed in the cortex, indicating that exogenous mitochondria promote remyelination and axonal regeneration, suggesting mitochondrial transplantation as a potentially valuable therapeutic approach for ischemic stroke [230]. Excess oxidative stress impairs axon regeneration, while healthy mitochondria can enhance antioxidant defenses, creating a cellular environment conducive to axon growth. Research has found that oxidative damage markers significantly decrease in neurons after mitochondrial transplantation, while axon regeneration capacity increases [172, 231].

Collectively, these findings highlight the critical role of mitochondrial transplantation in restoring mitochondrial function and promoting cell repair and regeneration across diverse pathological conditions. Mitochondrial transplantation supports cell repair and regeneration through multiple mechanisms.

### 3.6 Antitumor Effect

In cancer cells, mitochondria undergo adaptive reprogramming to sustain rapid proliferation in hypoxic and acidic microenvironments [232]. However, these adaptations render mitochondria vulnerable to further dysfunction. Cancer cells often rely on defective mitochondrial pathways, thus increasing ROS production, impairing OXPHOS, and causing Warburg effect, a metabolic shift toward aerobic glycolysis [233]. This metabolic rewiring supports rapid proliferation, evasion of apoptosis, and adaptation to hypoxic environments, while simultaneously conferring resistance to standard chemotherapeutics and radiotherapies. Mitochondrial transplantation offers a strategy to restore mitochondrial function, counteract metabolic vulnerabilities, and resensitize cancer cells to conventional treatments.

The reprogramming of cellular metabolism is a cancer hallmark and mitochondrial function restoration via mitochondrial transplantation provides a means to counteract these adaptations. Studies have shown that introducing healthy mitochondria into metabolically dysregulated cancer cells can reinstate OXPHOS and disrupt glycolytic dependence. Spees et al. [234] have demonstrated that the transfer of mitochondria from fibroblasts to mtDNA‐depleted A549 cells reinstates OXPHOS activity, improves oxygen utilization, and suppresses malignancy. Similarly, Sun et al. [235] highlighted that mitochondrial transfer to glioma cells reduces glycolysis, mitigates the Warburg effect, and enhances radiosensitivity. These findings suggest that mitochondrial transplantation could address the energy imbalance in cancer cells, reducing their proliferative and invasive capabilities.

Beyond metabolic correction, mitochondrial transplantation triggers intrinsic cell death pathways in cancer cells. The introduction of functional mitochondria activates apoptosis‐related proteins and suppresses antiapoptotic mechanisms. Chang et al. [236] reported that mitochondrial transfer to MCF‐7 breast cancer cells promotes the nuclear translocation of apoptosis‐inducing factor (AIF), triggering apoptotic cell death. Similarly, Yu et al. [237] demonstrated that mitochondrial transplantation in melanoma models downregulated Bcl‐2 expression while upregulating autophagy‐related genes, thereby promoting apoptosis and autophagic cell death. Moreover, this approach silenced transcription of proliferation genes through histone methylation, further emphasizing its potential to halt tumor growth.

Chemoresistance remains one of the most significant challenges in oncology, and mitochondrial transplantation offers a unique avenue to overcome it. Cancer cells often exploit dysfunctional mitochondria and ROS‐mediated signaling to develop resistance to chemotherapeutic agents. Elliott et al. [238] demonstrated that mitochondrial transfer to breast cancer cells enhanced their sensitivity to drugs such as doxorubicin, abraxane, and carboplatin, effectively reversing their resistance. Additionally, by restoring redox balance and reducing ROS levels, mitochondrial transplantation attenuates ROS‐induced adaptive pathways that contribute to chemoresistance. Radiosensitization has also been observed in gliomas, where mitochondrial transplantation restored metabolic pathways that rendered tumor cells vulnerable to radiation therapy [235].

Hypoxia is a key factor in tumor progression and therapy resistance by driving glycolysis and promoting an acidic microenvironment conducive to tumor survival. The ability of mitochondrial transplantation to mitigate hypoxia‐induced metabolic stress has profound implications for targeting aggressive and refractory tumors. Research revealed that mitochondrial transfer from fibroblasts to hypoxic A549 cells not only reinstated OXPHOS but also reduced oxygen deprivation and tumor aggressiveness [234]. Additionally, mitochondrial transplantation reduces hypoxia‐inducible factor 1‐alpha (HIF‐1α) activity, a critical regulator of cellular adaptation to hypoxia, by reactivating proline hydroxylase 2, a redox‐sensitive enzyme that targets HIF‐1α for degradation under normoxic conditions. This process interrupts hypoxia‐driven oncogenic signaling, further reducing cancer cell survival and invasiveness [239].

ROS are closely linked to cancer progression due to their role in mitochondrial dysfunction, DNA damage, and activation of signaling pathways driving cellular proliferation [233, 240]. Elevated ROS levels in cancer cells reflect underlying mitochondrial abnormalities, promoting both oxidative stress and tumor growth. Importantly, mitochondrial transplantation offers a mechanism to regulate ROS production and restore cellular redox homeostasis. Transplanted mitochondria reduce ROS generation, thereby mitigating oxidative damage and suppressing redox‐sensitive pathways associated with enhanced proliferation, such as MAP kinase signaling [241]. The relationship between ROS and cancer progression is complex and highly dependent on the tumor type. In some cancers, such as pancreatic cancer, ROS‐driven oxidative stress fuels tumorigenesis and metastasis, suggesting that strategies combining mitochondrial transplantation with antioxidant therapy could be particularly effective [242]. Conversely, cancers such as metastatic lung carcinoma and melanoma are sensitive to oxidative stress, with ROS playing a critical role in suppressing their proliferation and metastatic potential. In these cases, reducing ROS levels through antioxidant therapy could inadvertently promote tumor progression and metastasis [243, 244]. To optimize therapeutic outcomes, mitochondrial transplantation approaches should be tailored to the specific oxidative profile of each cancer type. For ROS‐sensitive tumors, mitochondrial transplantation combined with oxidative stress‐inducing strategies may maximize apoptosis and inhibit tumor growth. In contrast, ROS‐driven cancers might benefit from a synergistic application of mitochondrial transplantation and antioxidant interventions to disrupt the oxidative feedback loop that sustains tumor progression. Therefore, determining the ROS dependency of cancer cells will be a pivotal factor in designing effective redox‐targeted therapies in conjunction with mitochondrial transplantation.

In conclusion, mitochondrial transplantation demonstrates significant antitumor effects by restoring OXPHOS, inducing apoptosis, overcoming chemoresistance, regulating ROS levels, and alleviating hypoxia, offering new possibilities for effective cancer treatment.

### 3.7 Enhancement of Synaptic Plasticity, Neurotrophic Factor Expression, and Neural Circuit Connectivity

**FIGURE 2:**
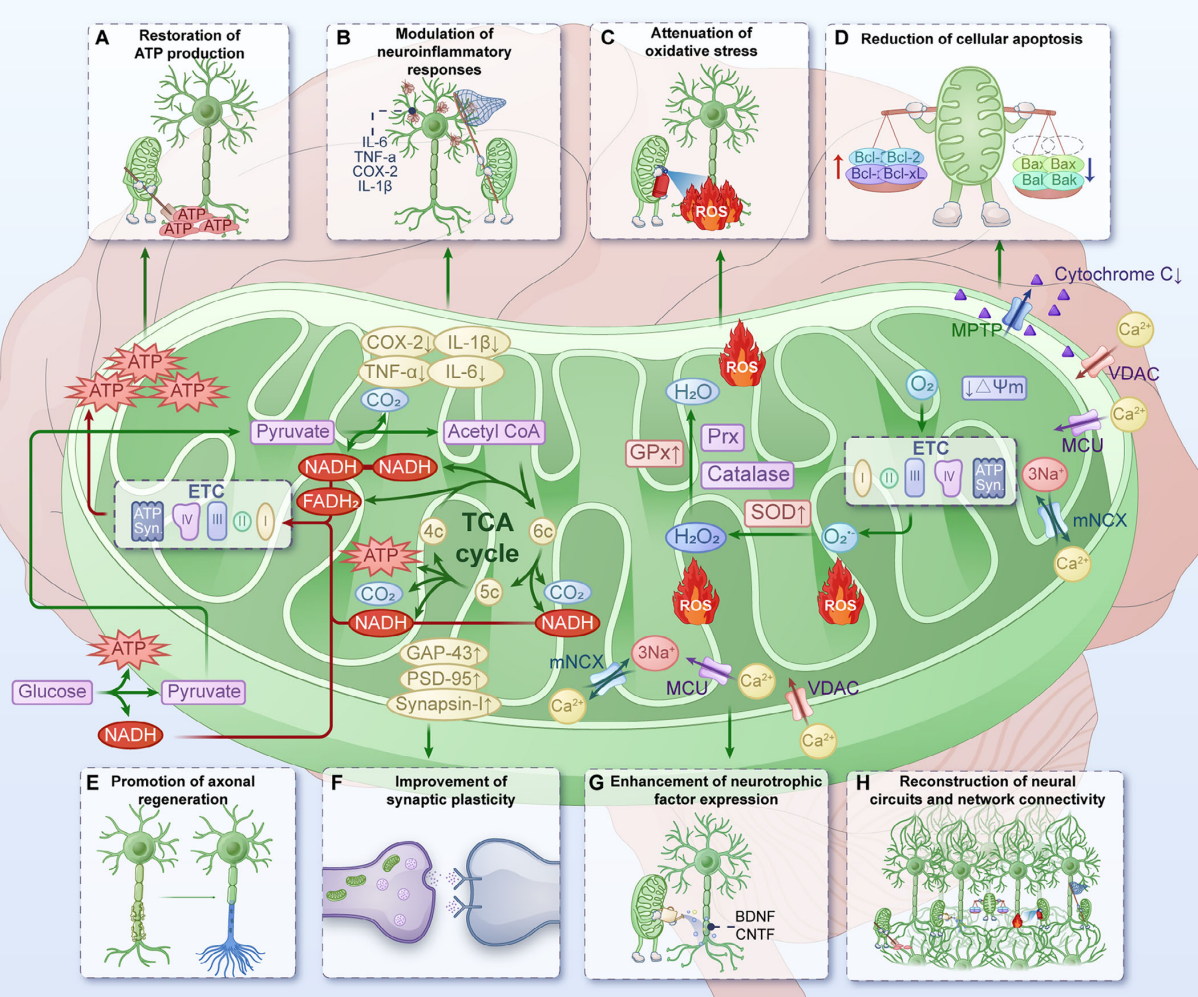
The roles of mitochondrial transplantation in neurological disorders. (A) Restoration of ATP production; (B) modulation of neuroinflammatory responses; (C) attenuation of oxidative stress; (D) reduction of cellular apoptosis; (E) promotion of axonal regeneration; (F) improvement of synaptic plasticity; (G) enhancement of neurotrophic factor expression; and (H) reconstruction of neural circuits and network connectivity.

Notably, mitochondrial transplantation demonstrates additional roles and mechanisms in addressing neurological disorders, including enhancing synaptic plasticity, promoting neurotrophic factor expression, and improving neural circuit reconstruction and network connectivity (Figure 2).

Synaptic plasticity is the foundation of neural adaptability and learning and memory capabilities, crucial for the recovery and maintenance of neurological functions [245, 246]. Mitochondrial transplantation can significantly increase ATP levels in synaptic regions, providing sufficient energy support for synaptic plasticity‐related processes. Healthy mitochondria support synaptic function by ensuring adequate ATP supply, which is necessary for synaptic vesicle cycling and neurotransmitter release, thus enhancing synaptic plasticity and connectivity [247, 248]. Another important mechanism is the effect of mitochondrial transplantation on synaptic mitochondrial dynamics. Synaptic mitochondria distribution and movement are crucial for synaptic plasticity [249]. Studies have shown that mitochondrial transplantation can improve mitochondrial transport dynamics, promoting their rapid localization to high energy consumption areas when needed [210]. Additionally, mitochondrial transplantation can regulate synaptic protein synthesis and transport. Synaptic plasticity depends on the synthesis and localization of new proteins [250]. Healthy mitochondria not only provide energy for protein synthesis, but also participate in the axonal transport process of proteins [251]. Research has found that the expression and localization of synaptic‐related proteins, such as postsynaptic density protein 95, synapsin‐I, and growth‐associated protein 43, significantly improve after mitochondrial transplantation [215, 252].

Neurotrophic factors are essential for neuron survival, growth, and function, playing key roles in neural system development, maintenance, and repair [253]. Mitochondrial function is closely related to neurotrophic factor expression. Studies have shown that mitochondrial dysfunction can decreased neurotrophic factor expression, which can be reversed by introducing healthy mitochondria. For example, in a study on a rat model of sciatic nerve crush injury, mitochondrial transplantation facilitated the entry of exogenous mitochondria into nerve cells and restored mitochondrial function, significantly enhancing the expression levels of brain‐derived neurotrophic factor (BDNF) and ciliary neurotrophic factor (CNTF) [172]. In addition, mitochondrial transplantation significantly increased BDNF expression in mouse models of depression [177] and TBI [180]. The synthesis and secretion of neurotrophic factors are energy‐dependent processes requiring adequate ATP supply [254]. By providing healthy mitochondria, cellular energy metabolism can be optimized, providing the necessary energy support for neurotrophic factor production. Furthermore, mitochondrial transplantation can regulate neurotrophic factor expression by influencing intracellular calcium balance. Calcium ions are vital signaling molecules in controlling neurotrophic factor expression [255]. Mitochondria play a crucial role in maintaining cellular calcium homeostasis [256] and introducing healthy mitochondria can optimize calcium signaling, thereby promoting neurotrophic factor expression [75].

Neural circuits are functional networks composed of interconnected neurons that communicate through synapses to achieve specific physiological functions and behaviors [257]. Although individual neurons are the basic units of the nervous system, they work together in neural circuits with specific synaptic connection patterns, playing critical roles in sensory perception, motor control, memory, emotion, and cognition [257]. First, mitochondrial transplantation can promote neuronal survival. In neural injury or degenerative diseases, neuron survival and neurogenesis are crucial for maintaining and reconstructing neural circuits [175, 180, 210, 258]. Mitochondrial transplantation can significantly improve neuronal survival rates and functional recovery by augmenting energy metabolism, preventing apoptosis, and reducing oxidative stress [175, 178]. Second, mitochondrial transplantation can promote axonal regeneration by providing sufficient energy support. Axonal regeneration is a key step in neural circuit reconstruction and requires a large amount of energy [259, 260]. Studies have shown that the introduction of healthy mitochondria can significantly increase ATP production in neurons, providing the necessary energy support for axonal growth [227, 228, 261]. Third, mitochondrial transplantation can enhance synaptic plasticity, the basis for neural network reorganization and functional recovery [246, 259]. Mitochondrial localization and function at synapses are crucial for synaptic transmission and plasticity. Synaptic mitochondrial function can be improved by providing healthy mitochondria, thereby enhancing synaptic plasticity and the ability of neural networks to reorganize [215, 252]. Furthermore, mitochondrial transplantation can regulate the expression and release of neurotrophic factors, such as BDNF and CNTF, which play important roles in neural circuit reconstruction [262, 263]. Research has found that the introduction of healthy mitochondria can upregulate the expression of these neurotrophic factors, thereby promoting neuronal growth and synapse formation [172, 180]. Another important aspect is that mitochondrial transplantation significantly promotes neural circuit reconstruction by enhancing the proliferation of oligodendrocyte progenitor cells (OPCs), which are indispensable for myelination [264, 265]. Mitochondrial transplantation enhances OPC proliferation, thereby boosting their capacity for myelin repair and promoting neural circuit reconstruction [257, 260]. Finally, mitochondrial transplantation can optimize neural network function by regulating the electrophysiological properties of neurons. Mitochondria play an important role in maintaining neuronal membrane potential and regulating ion channel activity [69]. The introduction of healthy mitochondria can improve the electrophysiological properties of neurons, thereby optimizing the information processing and transmission capabilities of neural networks [69, 266]. In conclusion, mitochondrial transplantation promotes neural circuit reconstruction and enhances network connectivity through multiple mechanisms, including promoting neuronal survival, supporting axonal regeneration, enhancing synaptic plasticity, regulating neurotrophic factor expression, promoting myelination, and regulating neuronal electrophysiological properties.

## 4 Mitochondrial Transplantation in Experimental Studies of Disorders

Exogenous mitochondrial transplantation originated from in vitro studies. In 1982, Clark and Shay [267] first discovered that mitochondria could be transferred between different cells. Subsequently, numerous in vitro experiments confirmed that exogenous mitochondria could stably integrate into recipient cells and restore their vitality. The development of mitochondrial transplantation as a therapeutic approach can be traced back to the early 2000s. In 2006, McCully et al. [268] demonstrated the potential of autologous mitochondrial transplantation in protecting the heart from I/R injury, marking a significant milestone in this field. Since then, mitochondrial transplantation applications in numerous diseases, such as neurodegenerative diseases, ischemic stroke, and spinal cord injury, have been explored [269].

### 4.1 In Vivo Studies of Mitochondrial Transplantation for Treating Disorder Models

**FIGURE 3:**
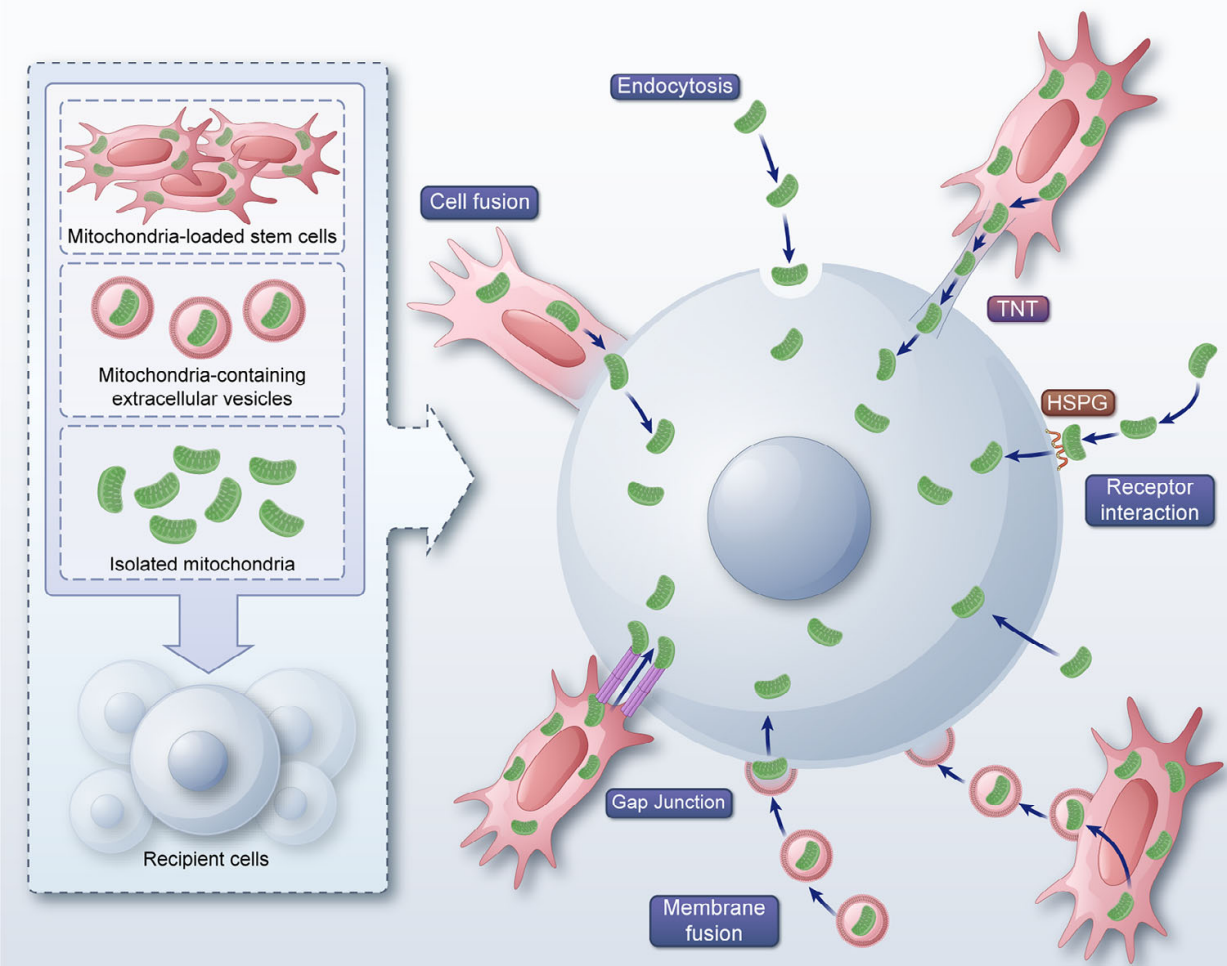
Patterns of mitochondrial transfer from different sources to recipient cells.

Mitochondrial transplantation has recently gained traction as a novel treatment paradigm, showing significant potential in multiple in vivo disease and injury models. Studies have shown that mitochondrial transplantation can effectively improve symptoms and prognosis in neurological disorder, cardiovascular disease, hepatic disease, pulmonary disease, renal disease, musculoskeletal disorder, inflammatory condition, and neoplasm models (Table 1). The routes of mitochondrial transplantation are varied, encompassing local injection, intravenous injection, intracerebroventricular injection, intranasal administration, and others (Table 1). The sources of mitochondria for transplantation are also diverse, including healthy mitochondria isolated from the liver, muscle, and platelets, as well as mitochondria from mitochondria‐containing extracellular vesicles (EVs) and mitochondria‐loaded stem cells (Table 1). The source of mitochondria varies, and the patterns of transferring mitochondria from different sources to cells also differ (Figure 3). Isolated mitochondria can be internalized by cells through endocytosis or via interaction with cellular surface receptors, such as heparan sulfate proteoglycans [270, 271]. Mitochondria‐containing EVs can be absorbed by cells through membrane fusion [270, 272]. Mitochondria‐loaded stem cells transfer mitochondria to cells via TNT, EVs, cell fusion, and gap junctions [273, 274, 275, 276].

**TABLE 1:** In vivo studies of mitochondrial transplantation for treating disorder models.

Disease	Animal model of disease	Source of mitochondria	Route of transplantation	Recipient	Outcome	References
Neurological disorders	6‐OHDA‐induced rat model of PD	PC12 cells and human osteosarcoma cybrids harboring wild‐type mitochondrial DNA derived from human skin fibroblasts	Local injection into the medial forebrain bundle	Brain neurons (SN neurons)	Improvement in motor activity; reduced dopaminergic neuron loss; recovery of mitochondrial dynamics; reduction in oxidative DNA damage	[210]
	MPTP‐induced mouse model of PD	Human hepatoma cells	Intravenous injection	Various tissues of the MPTP‐induced mouse model of PD, including the brain, liver, kidneys, muscles, and heart	Enhanced behavioral performance and biochemical parameters in the MPTP‐induced PD mouse model through improved electron transport chain activity, reduced ROS levels, and apoptosis and necrosis inhibition	[175]
	6‐OHDA‐induced rat model of PD	Rat liver	Intranasal delivery	The SN and striatum regions of the brain	Improved rotational and locomotor behaviors in PD rats, with >60% dopaminergic neuron survival in the SN and striatum; oxidative damage in the lesioned SN was attenuated.	[277]
	Intracerebroventricular amyloid‐β‐injected AD model mice	HeLa cells	Tail intravenous injection	Brain cells	Enhanced cognitive performance, reduced neuronal loss and gliosis in the hippocampus	[278]
	5XFAD transgenic mice model of AD	HeLa cells	Intravenous injection	Brain and liver	Improved cognitive function with reduced neuronal damage and amyloid burden; increased mitochondrial enzyme activities in the brain and liver; proteomic alterations in the hippocampus revealed changes in mitochondrial factors	[279]
	Aged mouse model of age‐associated cognitive decline and mitochondrial dysfunction	Liver of young mice (2‐month‐old mice)	Inject the mitochondrial suspension into the hippocampus of aged mice using stereotactic techniques	Hippocampus of aged mice	Elevated ATP levels; enhanced mitochondrial complex I, II, and IV activities, and reduced Tom20 expression; improvement in novel object recognition and spatial memory; stimulated neurogenesis and progenitor cell proliferation	[280]
	DACI mouse model	Platelets of healthy rats	Intracerebroventricular injection	Hippocampal neurons of mice	Alleviated DACI, increased mitochondrial number, restored mitochondrial function, attenuated oxidative stress and neuronal apoptosis, and decreased Aβ and Tau accumulation in the hippocampus	[281]
	Aged rat model of chronic mild stress‐induced depression	Brain tissue of young rats	Intracerebroventricular injection	Prefrontal cortex of aged rats	Reduced immobility time in forced swimming test, increased open arm time and entries in elevated plus maze, improved activity levels in open field test; restored mitochondrial membrane potential and ATP levels, decreased IDO and kynurenine levels; increased dendritic length and spine density of neurons in the prefrontal cortex	[282]
	Aged mice model of aging‐related cognitive and motor function decline	Liver of young mice	Intravenous injection	Multiple tissues of aged mice, including brain, skeletal muscle, liver, kidney, lung, and heart	Increased ATP content and decreased ROS levels in tissues. Improved cognitive function: better performance in water maze test; enhanced motor function: better performance in forced swimming and rotarod tests; increased phagocytic activity of macrophages	[283]
	Mouse model of transient focal cerebral I/R injury	Cerebral cortex of mice	Intraventricular injection	Brain (lateral ventricles)	Treatment with O‐GlcNAc‐modified mitochondria reduced neuronal injury and improved neurological deficits	[17]
	Mouse model of TBI	Allogeneic mouse liver and autologous mouse muscle	Injected into the cerebral cortex	Brain (injured cortex)	Reduced neuronal apoptosis, induced significantly upregulated BDNF in reactive astrocytes, improved spatial memory, enhanced motor function, and alleviated anxiety in mice	[180]
	Ischemic stroke mouse model	Mouse bone marrow MSCs	Intranasal administration	Brain (medial prefrontal cortex)	Improved ischemia‐induced memory impairment; reduced ROS levels, restored ATP production; increased synaptic marker expression in the ischemic area	[215]
	Rat model of schizophrenia	Human lymphocyte and rat brain	Bilateral injection of into medial prefrontal cortex	Brain (prefrontal cortex)	Prevented dissipation in mitochondrial membrane potential and emergence of schizophrenia‐like attention deficit in adulthood	[216]
	LPS‐induced mouse model of depression	Hippocampus of normal mice	Intravenous injection	Brain (hippocampus)	Improved depression‐like behaviors in mice; reduced activation of astrocytes and microglia, and decreased neuroinflammation; increased BDNF expression and enhanced neurogenesis; restored ATP production in the hippocampus and reduced oxidative stress	[177]
	Rat model of SCI	PC12 cells and rat soleus muscle	Injected into spinal cord	Spinal cord	Significantly maintained the short‐term bioenergetic state, particularly in terms of oxygen consumption rate	[284]
	Rat model of spinal cord ischemia	Rat soleus muscle	Delivered via the internal jugular vein	Spinal cord (ischemic area)	Enhanced lower‐limb locomotor function, reduced inflammation and cell death in the affected region, and increased Nissl body density in ventral horn neurons of the ischemic spinal cord	[195]
	Rat model of SCI	Rat soleus muscle	Injected into spinal cord	Spinal cord (injured area)	Restored motor and sensory functions; reduced the degree of demyelination; reduced mitochondrial fragmentation, neuroapoptosis, neuroinflammation, and oxidative stress	[187]
	Rat model of optic nerve crush	Rat liver	Intravitreal injections	Retina	Improved retinal oxidative metabolism and electrophysiological activity; increased the survival rate of retinal ganglion cells and the number of axons	[228]
	Rat model of cerebral I/R injury	Neuro‐2a cells and mouse NSCs	Internal carotid artery injection	Brain (ischemic area)	Improved neurological deficits, reduced cerebral infarct size, and transcriptomic analysis indicating therapeutic effects mediated via metabolism‐related pathways, particularly those involving lipid metabolism	[213]
	AIS rat model	Human umbilical cord‐derived MSCs	Intracerebroventricular injection	Brain (ischemic area)	Mitigated I/R‐induced injury; reduced serum creatine phosphokinase levels; decreased cell apoptosis; reduced activation of astrocytes and microglia; decreased the infarct size in the brain; improved motor function and coordination in rats	[285]
	AIS rat model	Pectoralis major muscle of rat	Intracerebroventricular injection	Brain (neurons around the ischemic penumbra)	Mitigated I/R‐induced injury; reduced brain infarct volume; decreased cell apoptosis and cellular oxidative stress; reduced activation of astrocytes; promoted neurogenesis; improved motor function and coordination in rats	[181]
	Rat brain ischemic stroke model induced by MCAO	BHK‐21 cells	(1) Intracerebral injection (2) Femoral artery injection	Brain (ischemic area)	Restored motor functions; reduced the number of apoptotic cells, the intensity of apoptotic signals, and the lesion area in ischemic brain tissues	[186]
	Mouse model of transient focal cerebral I/R	Cryopreserved mouse placenta	Intravenous injection	Brain, lung, liver, kidney, and heart	Reduced infarct area in mice	[286]
	Focal cerebral ischemia mouse model	Allogeneic mouse liver	Intracerebral injection	Brain (ischemic area)	Enhanced mitochondrial complex activity and ATP levels in the ischemic cortex, reduced apoptosis, and promoted proliferation of oligodendrocyte progenitor cells; increased myelin basic protein expression, decreased demyelinated axons, and improved locomotor recovery, including forelimb activity and rotarod performance	[230]
	Mouse model of TBI	Mouse brain tissue	Intracerebral injection	Brain (injured area)	Enhanced cellular respiration and increased synaptic plasticity‐related protein expression; increased neovascularization in the injured area; reduced blood–brain barrier damage and brain edema	[252]
	Mouse model of sepsis	Pectoralis major muscle of mouse	Intracerebroventricular injection	Brain	Microglia in mice shifted from the M1 phenotype to the M2 phenotype, reducing the release of proinflammatory cytokines; improved cognitive function in septic mice	[202]
	Rat model of sciatic nerve crush injury model	BHK‐21 cells	Injected into sciatic nerve	Injured sciatic nerve	Reduced oxidative stress, increased neurotrophic factor expression, restored muscular integrity, increased muscle weight, and improved animal neurobehavior and electrophysiological function	[172]
	Rat model of SCI	Primary rat bone marrow MSCs	Injected into spinal cord	Spinal cord (injured area)	Reduced neuronal apoptosis at the spinal cord injury site; increased the expression of neurotrophic factors, promoting nerve regeneration and remyelination	[287]
	Rat model of cerebral I/R injury	Primary rat bone marrow MSCs	Common carotid artery injection	Brain (ischemic area)	Reduced the infarct volume; increased the density of newly formed microvessels and improved the mitochondrial function of the cerebral microvascular system	[173]
	Rat brain ischemic stroke model induced by MCAO	MMSCs	Mitochondrial transfer was achieved through intravenous injection of MMSCs	Brain (ischemic area)	Reduced the infarct volume; improved neurological function	[288]
	Rat brain ischemic stroke model induced by MCAO	Livers of rats	Intracerebroventricular injection	Brain (ischemic area)	Reduced brain infarct area; improved neurological function; reduced inflammatory response; decreased oxidative stress; inhibited cell apoptosis	[289]
	Mouse model of focal cerebral ischemia	Mouse cortical astrocytes	Injected into the peri‐infarct cortex	Brain (peri‐infarct cortex)	Neurons receiving mitochondria upregulated cell survival‐related proteins; amplified neuronal survival signals; reduced extracellular mitochondria transfer; promoted neuroplasticity	[178]
	Olfactory bulbectomized mice with Alzheimer's type degeneration	Mouse brain	Intranasal delivery	Brain (neocortex and hippocampus)	Improved spatial memory in mice	[290]
	Mouse model of cisplatin‐induced cognitive deficits	Human MSCs	Intranasal delivery	Brain meninges and parenchyma (various other brain regions including the ventricles, choroid plexus, hippocampus, olfactory bulb)	Restoration of executive function, spatial memory, and working memory; repair of white matter integrity; reversal of synaptic loss; improvement of abnormal synaptic mitochondrial structure; regulation of the hippocampal transcriptome	[291]
	Mouse model of cisplatin‐induced neurotoxicity	MSCs derived from mice	Intranasal delivery of MSCs	Neural progenitor cells in the dentate gyrus of the hippocampus and the subventricular zone	Reduction of cisplatin‐induced loss of DCX^+^ neural progenitors	[292]
	Mouse model of myelin oligodendrocyte glycoprotein‐induced EAE	NSCs from mice	Intraventricular injection	Mononuclear phagocytes, astrocytes, neurons, oligodendrocytes, and T cells	Ameliorated the clinical deficits in EAE mice	[293]
	Mouse model of ischemic stroke	Human cerebral microvascular endothelial cell line	Intravenous delivery of mitochondria‐containing extracellular vesicles	Brain (ischemic area)	Increased ATP levels; improved mitochondrial function; reduced brain infarct size	[294]
Cardiovascular diseases	Rabbit model of focal cardiac ischemia	Rabbit heart tissue unaffected by ischemia	Directly injected into the ischemic area	Heart (ischemic area)	Improved postischemic function and cell viability	[268]
	Rabbit model of focal cardiac ischemia	Rabbit pectoralis major tissue	Directly injected into the ischemic area	Cardiomyocytes	Enhanced myocardial oxygen utilization; increased synthesis of high‐energy phosphates; upregulated cytoprotective cytokine pathways; activated cardioprotective proteomic cascades; preserved cardiac energetic function.	[218]
	Rabbit model of focal or regional cardiac ischemia	Human cardiac fibroblasts; rabbit liver	Intracoronary injection	Heart	Reduced infarct size and improved myocardial function	[295]
	Pig model of focal cardiac ischemia	Pig pectoralis major tissue	Directly injected into the ischemic area	Cardiomyocytes	Reduced cardiac injury markers; decreased myocardial infarct size; enhance myocardial cell viability	[296]
	Swine model of focal cardiac ischemia	Swine pectoralis major tissue	Intracoronary injection	Heart	Decreased myocardial infarct size; enhanced postischemic myocardial function; improved coronary blood flow	[297]
	Pig model of regional cardiac ischemia	Pig pectoralis major tissue; swine cardiac fibroblast cell	Intracoronary injection	Heart	Preserved myocardial function and oxygen consumption	[298]
	Pig model of focal cardiac ischemia	Pig pectoralis major tissue	Direct injection into the left coronary ostium	Heart	Reduced myocardial infarct size and enhanced myocardial function	[299]
	Piglet model of right heart failure	Piglet gastrocnemius muscle	Intramyocardial injection	Cardiomyocytes	Prolonged physiological adaptation; reduced cardiomyocyte apoptosis; preserved right ventricular function	[217]
	Mouse model of heterotopic heart transplantation	Mouse gastrocnemius muscle	Intracoronary injection	Heart	Reduction in neutrophil infiltration; prevention of contraction band formation; enhancement in heart graft function; reduction in heart graft tissue injury	[300]
	Rat model of regional cardiac ischemia	Rat pectoralis major tissue	Intracoronary injection	Heart	Improved cardiac functional recovery; reduced infarct size; decreased left ventricular end‐diastolic pressure	[301]
	Mouse model of anthracycline‐induced cardiomyopathy	iPSC‐MSCs	Intramyocardial injection	Heart	Rescue anthracycline‐induced cardiomyocyte damage	[302]
	Mouse model of myocardial infarction	EVs from human iPSC‐derived cardiomyocytes	Intramyocardial injection	Cardiomyocytes	Activated mitochondrial biogenesis; enhanced postmyocardial infarction cardiac function; facilitated immediate mitochondrial and nonmitochondrial cargo transfer	[303]
Hepatic diseases	APAP‐induced liver injury mouse model	HepG2	Intravenous injection	Multiple organs including the liver, lungs, brain, and kidneys	Enhanced hepatocyte energy supply, reduced oxidative stress, and improved tissue injury	[304]
	APAP‐induced liver injury rat model	Rat MSCs	Injected into the subcapsular region of the spleen	Liver	Reduced plasma ALT levels; decreased apoptotic cells; lowered total oxidant levels; enhanced liver histological structure	[305]
	Carbon tetrachloride‐induced liver injury mouse model	Liver mitochondria from healthy mice	Intravenous injection	Liver, lung and kidney, and a small amount in heart	Restored cell viability; prevented tissue fibrogenesis; rehabilitated mitochondrial function; enhanced cellular homeostasis	[182]
	Fatty liver mouse model induced by a high‐fat diet	HepG2	Intravenous injection	Liver, lung, brain, muscle, and kidney	Reduced serum aminotransferase activity; decreased cholesterol levels; reduced lipid accumulation; decreased oxidative injury in fatty liver; improved energy production; restored hepatocyte function	[306]
	Partial liver I/R mouse model	Rat liver tissue	Splenic injection	Liver parenchyma	Reduction in serum ALT levels; reduction in liver tissue injury; decreased apoptosis; reduced cytochrome *C* release and caspase 9 expression; reduction in oxidative stress	[307]
	Liver I/R mouse model	Human umbilical cord‐derived MSC‐EVs	Intravenous injection	Intrahepatic neutrophils	Inhibition of neutrophil extracellular traps formation; repair of mitochondrial function in intrahepatic neutrophils	[308]
Pulmonary diseases	Porcine I/R model in lung transplantation	Porcine heart tissue	Lung perfusion	Lung	Reduction in lung tissue damage; reduced inflammatory response; decreased oxidative stress; improved cell viability	[309]
	I/R injury‐induced mouse model in acute lung injury	Mouse gastrocnemius muscle	Pulmonary artery injection or nebulization	Lung (lung alveoli and connective tissue)	Improved lung mechanics; reduced lung tissue injury; decreased neutrophil infiltration; decreased interstitial edema; reduced apoptosis	[310]
	Bleomycin‐induced pulmonary fibrosis mouse model	Human MSCs	Tail vein injection	Lung	Enhanced intercellular mitochondrial transfer	[311]
	LPS‐induced mouse model in acute lung injury	Mouse bone marrow‐derived stromal cells	Intranasal delivery	Alveolar epithelium	Increased alveolar ATP; protection against LPS‐induced acute lung injury; restoration of alveolar bioenergetics	[174]
	LPS‐induced mouse model in acute lung injury	Exosomes from human adipose‐derived MSCs	Tail vein injection	Alveolar macrophages	Increased mitochondrial integrity in macrophages; enhanced ATP production; reduced ROS stress; shift of macrophages to anti‐inflammatory phenotype; decreased secretion of proinflammatory cytokines; increased production of anti‐inflammatory cytokines	[183]
	LPS‐induced mouse model in acute lung injury	Mouse bone marrow MSCs	Intratracheal injection	Pulmonary microvascular endothelial cells	Improved endothelial barrier integrity; increased ATP generation and mitochondrial membrane potential; reduced cell apoptosis; alleviated pulmonary edema	[221]
Renal diseases	Streptozotocin‐induced rat model in diabetic nephropathy	Rat bone marrow‐derived stromal cells	Direct injection under the renal capsule	Proximal tubular epithelial cells	Improvement in cellular morphology of proximal tubular epithelial cells; restoration of tubular basement membrane and brush border structure	[188]
	Streptozotocin‐induced mouse model in diabetic nephropathy	Mouse bone marrow MSCs	Tail vein injection	Macrophages	Suppressed inflammatory response; ameliorated kidney injury	[200]
	Doxorubicin‐induced nephrotoxicity rat model	Rat MSCs	Direct injection into the renal cortex	Renal cortex	Decreased oxidative stress in renal cells; promotion of tubular cell regeneration; reduced protein accumulation in tubular cells; reversal of renal deficits; increased Bcl‐2 levels; decreased caspase‐3 levels in injured renal cells	[222]
	Ex vivo porcine model of donation after cardiac death renal transplantation mimicking I/R injury	Pig psoas muscle tissue	Renal artery injection	Kidney	Induced damage to kidneys, improved metabolic function, enhanced tissue stability, and modulated critical cellular signaling pathways	[312]
	I/R injury‐induced pig model in acute kidney injury	Pig sternocleidomastoid muscle	Renal artery injection	Kidney	Improved renal function; reduced kidney damage; mitigation of necrosis; lowered tubular injury; decreased inflammatory response; reduced IL‐6 expression	[313]
	I/R injury‐induced rat model in acute kidney injury	Rat pectoralis major muscle	Renal artery injection	Kidney	Prevented renal tubular cell death and effectively restored renal function; amelioration of kidney damage; enhanced tubular regeneration; reduction in apoptosis	[219]
	I/R injury‐induced mouse model in acute kidney injury	EVs from bone marrow‐derived MSCs	Tail vein injection	Liver, kidney, spleen, and lung	Attenuation in mitochondrial DNA damage; reduced inflammatory response; decreased renal lesion formation; attenuated mitochondrial damage	[314]
	I/R injury‐induced mouse model in acute kidney injury	EVs from human placenta‐derived MSCs	Intravenous injection	Renal proximal tubular epithelia cells	Activation of the Keap1–Nrf2 signaling axis; enhancement of tubular epithelial cell mitochondrial function	[315]
Musculoskeletal disorders	Collagenase injection‐induced rat model of tendinopathy	L6 rat myoblast cell	Tendon injection	Tendon	Reduced inflammatory and fission marker levels; restored collagen production	[316]
	Hindlimb I/R injury‐induced mouse model in acute limb ischemia	Mouse muscle	Direct injection into hindlimb muscles	Hindlimb muscles	Reduced muscle infarct area and cell apoptosis were significantly; improved limb function recovery	[317]
	BaCl_2_‐induced muscle injury mouse model	Mouse liver	Tail vein injection	Injured skeletal muscle	Accelerated muscle regeneration and functional recovery	[224]
	BaCl_2_‐induced muscle injury mouse model	mitochondria transferred C2C12 myoblasts	Direct injection into gastrocnemius muscles	Gastrocnemius muscle	Improved muscle regeneration and function	[318]
	Dexamethasone‐induced muscle atrophy rat model of	Human umbilical cord‐derived MSCs	Direct injection into soleus muscles	Soleus muscle	Significant muscle mass increase; marked lactate concentration reduction; elevation of desmin protein expression; substantial decrease in muscle‐specific ubiquitin E3 ligases	[223]
	Monosodium iodoacetate‐induced osteoarthritis rat model	L6 myoblast cell line	Direct injection into knee joint	Chondrocytes	Ameliorated pain; reduced cartilage destruction; improved bone loss; decreased inflammatory cytokine transcript levels; enhanced mitochondrial function in chondrocytes	[319]
	Calvarial defect rat model	Rat bone marrow MSCs	Transplantation into rat cranial bone defect sites	Cranial bone	Enhanced bone marrow MSC proliferation, osteogenesis, migration; increased ATP production; enhanced therapeutic effects on in situ bone defect repair	[184]
	Calvarial defect mouse model	Mitochondria and EVs secreted from mature osteoblasts	Collagen sponge containing mitochondria and EVs for filling calvarial defects	Cranial bone	Promoted differentiation of osteoprogenitors; enhanced bone regeneration in vivo	[225]
Inflammatory conditions	Cecal ligation and puncture‐induced sepsis rat model	Rat pectoralis major tissue	Tail intravenous injection	Heart	Enhanced mitochondrial function; restored mitochondrial biogenesis; reduced inflammatory cytokine levels	[16]
	LPS‐induced sepsis mouse model	Human umbilical cord MSCs	Intravenous injection	Multiple organs	Improved survival rates	[201]
	Cecal ligation and puncture‐induced sepsis mouse model	Mouse pectoralis major tissue	Tail intravenous injection	Multiple organs including the lungs, liver, kidneys, and brain	Reduced systemic inflammation and organ injury, enhanced bacterial clearance, and improved the survival rate	[320]
	Cecal ligation and puncture‐induced sepsis mouse model	Mesenchymal stromal cells	Intravenous injection	Multiple organs including the lungs, liver, and kidneys	Reduced multiple organs injury	[321]
	Cecal slurry model in rat	Rat muscle cell line and human umbilical cord MSCs	Tail intravenous injection	Spleen and muscle tissues	Improved survival rate; enhanced bacterial clearance; alleviated mitochondrial dysfunction; reduced spleen apoptosis; attenuated hyperinflammation; mitigated immune paralysis	[220]
	Polymicrobial fecal slurry peritonitis model in rat	L6 muscle cells, C9 hepatocytes, and human umbilical cord MSCs	Tail intravenous injection	Blood and organ tissues	Improved mitochondrial function; enhanced oxygen consumption; reduced hyperinflammation in the acute phase; strengthened immune response during immune suppression; improved survival	[322]
	Graft‐versus‐host disease mouse model	Human umbilical cord MSCs	Intraperitoneal injection	Mesenteric lymph node and spleen cells	Improved survival rate; decreased tissue damage; reduced inflammatory response; decreased infiltration of organ T CD4^+^, CD8^+^, and IFN‐γ expressing cells	[323]
Neoplasms	Glioma xenograft nude mouse model	Normal human astrocytes	Intratumor injection	Xenograft tumors (human glioma cells)	Inhibited xenograft tumor growth; enhanced glioma radiosensitivity	[235]
	Prostate and ovarian cancer xenograft nude mouse model	Human cardiac fibroblast cells	Not mentioned	Subcutaneous tumors	Enhanced chemotherapeutic sensitivity	[15]
	Mouse model of triple‐negative breast cancer	Mouse liver	Intratumor injection	Breast tumors	Attenuated oxidative stress and diminished cancer‐associated fibroblast numbers; mitochondrial fusion promotion in non‐necrotic regions; increased mitochondrial fusion proteins and Parkin expression	[324]
	Mouse model of triple‐negative breast cancer	Mouse liver	Intratumor injection	Breast tumors	Enhanced doxorubicin sensitivity via mitochondrial fusion and mitophagy; induced cell death; inhibited nuclear factor kappa‐B activation	[325]
	Mouse model of subcutaneous melanoma; mouse model of metastatic melanoma in lungs	Mouse liver	Intravenous injection	Melanoma tissues	Reduced tumor volume and weight; decreased levels of pyruvate kinase, lactate, pyruvate and ATP; increased cell apoptosis	[237]
	Mouse model of metastatic melanoma in lungs	Mouse liver	Intravenous injection	Melanoma tissues	Inhibited tumor growth; increased survival time; reduced glycolysis and glutaminolysis in tumor cells; increased ROS levels and oxidative stress; decreased ATP production; increased tumor protein 53 levels; induced tumor cell death through apoptosis and necrosis	[326]
	Subcutaneous tumor model of hepatocellular carcinoma in mouse	Mouse liver	Intravenous injection	Hepatocellular carcinoma tissues	Inhibition of hepatocellular carcinoma cell proliferation; induction of cell cycle arrest and apoptosis; decrease in aerobic glycolysis; downregulation of cell cycle proteins; upregulation of apoptosis‐related proteins	[239]

*Abbreviations*: 5XFAD, 5x familial Alzheimer's disease; 6‐OHDA, 6‐hydroxydopamine; AD, Alzheimer's disease; AIF, apoptosis‐inducing factor; AIS, acute ischemic stroke; ALT, alanine aminotransferase; APAP, acetaminophen; ATP, adenosine triphosphate; Aβ, amyloid‐beta; Bcl‐2, B‐cell lymphoma 2; BDNF, brain‐derived neurotrophic factor; BHK, baby hamster kidney fibroblasts; DACI, diabetes‐associated cognitive impairment; DCX^+^, doublecortin positive; DNA, deoxyribonucleic acid; EAE, experimental autoimmune encephalomyelitis; EVs, extracellular vesicles; HepG2, human hepatoma cell line; I/R, ischemia/reperfusion; IDO, indoleamine 2, 3‐dioxygenase; IL‐6, interleukin‐6; iPSCs, induced pluripotent stem cells; LPS, lipopolysaccharide; MCAO, middle central artery occlusion; MMSCs, mesenchymal multipotent stromal cells; MPTP, 1‐methyl‐4‐phenyl‐1,2,3,6‐tetrahydropyridine; MSCs, mesenchymal stem cells; NSCs, neural stem cells; PC12, pheochromocytoma cell line; PD, Parkinson's disease; ROS, reactive oxygen species; SCI, spinal cord injury; SN, substantia nigra; Tau, microtubule‐associated protein tau; TBI, traumatic brain injury; Tom20, translocase of outer mitochondrial membrane 20.

In PD models, mitochondrial transplantation significantly improved motor activity, reduced dopaminergic neurons loss, restored mitochondrial complex I protein and mitochondrial dynamics, and reduced oxidative DNA damage [210]. Intravenous mitochondria injection increased ETC activity, reduced ROS levels, and prevented cell apoptosis and necrosis [175]. Intranasal delivery also significantly improve rotational and locomotor behaviors in PD rats, promoting dopaminergic neuron survival and recovery [277]. In AD models, mitochondrial transplantation significantly improved cognitive performance, reduced neuronal loss and gliosis, and ameliorated mitochondrial dysfunction in the brain [278]. In 5x familial AD (5XFAD) transgenic mouse model, mitochondrial transplantation not only improved cognitive function, but also reduced neuronal damage and amyloid burden, while affecting hippocampal proteome and liver and serum metabolome [279]. In aged mouse models, mitochondrial transplantation elicited canonical Wnt signaling activation in hippocampal neural progenitor cells, significantly stimulating neurogenesis and neural progenitors proliferation [280]. Another study showed that mitochondrial transplantation improved cognitive and motor performance in aged mice, increased ATP content in tissues, decreased ROS levels, and increased phagocytic activity of macrophages [283]. In aged rats subjected to chronic mild stress modeling depression, mitochondrial transplantation ameliorated anxiety‐depressive phenotypes, restored mitochondrial membrane potential and ATP levels, and increased dendritic length and spine density of neurons in the prefrontal cortex [282]. In LPS‐induced mouse model of depression, mitochondrial transplantation improved depression‐like behaviors, reduced astrocytes and microglia activation, decreased neuroinflammation, increased BDNF expression, and enhanced neurogenesis [177]. In rat model of SZ, mitochondrial transplantation prevented dissipation in mitochondrial membrane potential [216]. In cerebral I/R injury models, mitochondrial transplantation significantly improved neurological deficits and reduced cerebral infarct size, possible by influencing metabolism‐related pathways, particularly lipid metabolism‐related molecules and pathways [213]. In acute ischemic stroke models, mitochondrial transplantation mitigated I/R‐induced injury, reduced serum creatine phosphokinase levels, decreased cell apoptosis, reduced astrocytes and microglia activation, decreased the infarct size in the brain, and improved motor function and coordination [181, 285]. Moreover, placental mitochondria therapy reduced infarct area in mice [286]. Xenogenic mitochondrial transfer restore motor functions, reduce brain infarct areas, and decrease neuronal cell death in ischemic brain tissues [186]. In mouse model of transient focal cerebral I/R injury, treatment with O‐GlcNAc‐modified mitochondria reduced neuronal injury and improved neurological deficits [17]. In ischemic stroke mouse model, intranasal administration of mitochondria improved ischemia‐induced memory deficits and increased synaptic marker expression in the ischemic area [215]. In TBI mouse model, mitochondrial transplantation reduced neuronal apoptosis, induced significant BDNF upregulation in reactive astrocytes, improved spatial memory, enhanced motor function, and alleviated anxiety in mice [180]. In spinal cord injury models, mitochondrial transplantation maintained the short‐term bioenergetic state, particularly in terms of oxygen consumption rate [284]. It also enhanced lower‐limb locomotor function, suppressed inflammation and cell death, and increased the count of Nissl bodies of the lesioned cord [195]. Moreover, mitochondrial transplantation restored motor and sensory functions as well as reduced demyelination degree in the injured spinal cord, mitochondrial fragmentation, neuroapoptosis, neuroinflammation, and oxidative stress generation [187]. In rat model of optic nerve crush, mitochondrial transplantation improved retinal oxidative metabolism and electrophysiological activity, increased retinal ganglion cell survival rate and the number of axons [228]. These research findings demonstrate that mitochondrial transplantation can significantly improve neuronal survival, reduce cell apoptosis, enhance mitochondrial function, alleviate oxidative stress, promote neuroregeneration and remyelination, and improve cognitive and motor functions. Furthermore, mitochondrial transplantation can modulate neuroinflammatory responses, promote neuroplasticity, and improve neurological function. These discoveries provide important experimental evidence and theoretical basis for developing new therapeutic strategies for neurological diseases, while also paving the way for future clinical applications.

Recent studies have demonstrated significant progress in mitochondrial transplantation as a therapeutic strategy for various cardiovascular disorders, particularly in treating ischemic heart conditions. Multiple animal models, including rabbits, pigs, mice, and rats, have been employed to evaluate the efficacy and mechanisms of mitochondrial transplantation. Initial studies in rabbit models of focal cardiac ischemia showed that direct injection of autologous mitochondria into the ischemic area significantly enhanced postischemic functional recovery and cellular viability. In a pioneering study [268], researchers demonstrated that transplantation of functional mitochondria isolated from healthy cardiac tissue into pre‐reperfusion ischemic myocardium restored key measures of heart function. Another study using rabbit pectoralis major tissue showed that cardiomyocytes internalized transplanted mitochondria within 2–8 h, leading to cytoprotective pathways induction [218]. The therapeutic potential was also validated using human cardiac fibroblasts and rabbit liver mitochondria delivered through intracoronary injection, thus decreasing infarct size and improving postischemic myocardial function [295]. The therapeutic approach was successfully scaled to larger animal models. In pig models of focal cardiac ischemia, direct injection of autologous mitochondria derived from pectoralis major tissue reduced cardiac injury marker levels, decreased myocardial infarct size, and enhanced myocardial cell viability [296]. Intracoronary injection was equally effective, demonstrating reduced myocardial infarct size, enhanced postischemic myocardial function, and improved coronary blood flow [297]. Research using diabetic models demonstrated that mitochondrial transplantation could significantly enhance postischemic myocardial functional recovery in diabetic hearts [301]. In summary, mitochondrial transplantation in cardiovascular diseases through direct injection or intracoronary delivery has shown substantial potential in improving heart function, reducing myocardial injury, and enhancing cell viability in both acute and chronic heart disease models.

Mitochondrial transplantation also holds therapeutic potential in inflammation and immune‐mediated disease models. A rat model of cecal ligation and puncture (CLP)‐induced sepsis demonstrated that mitochondrial transplantation significantly improved survival rates and cardiac function. The treatment enhanced mitochondrial function, restored mitochondrial biogenesis, and reduced inflammatory cytokine levels. Notably, repetitive injections 1‐ and 7‐h post‐CLP provided superior protection than single injection protocols [16]. Similarly, muscle‐derived mitochondrial transplantation showed broad therapeutic effects in a CLP‐induced sepsis mouse model. The transplanted mitochondria were successfully distributed to multiple organs, including the lungs, liver, kidneys, and brain. This intervention enhanced bacterial clearance, reduced systemic inflammation, and improved survival rates [320]. MSC‐derived mitochondria have also shown particular promise. A study using MSC‐derived mitochondria‐rich fraction showed reduced lung, kidney, and liver injury in CLP‐induced sepsis. The treatment improved lung mechanics and histology while decreasing inflammatory markers [321]. In more complex models, such as the cecal slurry and polymicrobial fecal slurry peritonitis models, mitochondrial transplantation from various sources (including rat muscle cells, hepatocytes, and human umbilical cord MSCs) showed multiple beneficial effects, including enhanced bacterial clearance, improved mitochondrial function, and reduced hyperinflammation [220, 322]. In the context of graft‐versus‐host disease, mitochondrial transfer from MSCs to T cells showed remarkable therapeutic potential by increasing the expression of regulatory T‐cell differentiation markers and improving survival rates, while reducing tissue damage and inflammatory responses. This mechanism revealed a unique CD4^+^ T‐cell reprogramming pathway that could be exploited for immune disease treatment [323]. In conclusion, these studies demonstrate that mitochondrial transplantation represents a therapeutic strategy with broad application prospects in inflammatory and immune‐mediated diseases. The evidence consistently indicates that this approach can effectively modulate inflammatory responses and immune function while improving organ function and survival rates across different disease models.

In acetaminophen‐induced liver injury models, researchers have shown promising results using different sources of mitochondria. One study utilized mitochondria isolated from human hepatoma cells (HepG2) administered through intravenous injection, which resulted in widespread distribution across multiple organs, enhancing hepatocyte energy supply while reducing oxidative stress and tissue injury [304]. Another study in rat models employed mitochondria from rat MSCs, delivered via subcapsular splenic injection to reach the liver through portal circulation, which effectively reduced plasma alanine aminotransferase (ALT) levels, decreased apoptotic cell counts, lowered total oxidant levels, and enhanced liver histological structure [305]. Mitochondrial transplantation has also successfully treated CCl4‐induced liver injury by restoring cell viability, boosting ATP production, reducing oxidative damage, preventing fibrosis, and enhancing mitochondrial function through respiratory chain enzyme and mitophagy gene activation, ultimately promoting cellular homeostasis [182]. In high‐fat diet‐induced fatty liver disease models, where mitochondrial dysfunction is a major mechanism in developing nonalcoholic fatty liver disease, intravenous administration HepG2‐derived mitochondria demonstrated multiple beneficial effects. The treatment dose‐dependently reduced serum aminotransferase activity, decreased cholesterol levels, reduced lipid accumulation, decreased oxidative injury, improved energy production, and restored hepatocyte function [306]. Liver I/R injury has been addressed through different mitochondrial transplantation approaches. One study using rat liver tissue‐derived mitochondria delivered via splenic injection, which maintained high membrane potential of the isolated mitochondria and distribution in the liver parenchyma, reducing serum ALT levels and apoptosis [307]. Another innovative study utilized human umbilical cord‐derived MSC‐EVs administered intravenously, which contained functional mitochondria transferred to intrahepatic neutrophils. This transfer triggered mitochondrial fusion, specifically targeting intrahepatic neutrophils, inhibiting neutrophil extracellular trap formation, and repairing mitochondrial function in intrahepatic neutrophils, highlighting the therapeutic value of mitochondrial transplantation for liver I/R injury [308]. In summary, these studies collectively demonstrate the remarkable therapeutic potential of mitochondrial transplantation across diverse liver disease models. The transplanted mitochondria show consistent therapeutic effects across different liver pathologies such as reducing oxidative stress, improving energy metabolism, enhancing cell viability, and decreasing tissue damage.

In a porcine I/R model of lung transplantation, researchers utilized porcine heart tissue‐derived mitochondria delivered through lung perfusion, resulting in reduced tissue damage, decreased inflammatory response, and improved cell viability. The study revealed that transplanted mitochondria induced autophagy, which may be crucial for mediating anti‐inflammatory and antioxidant benefits [309]. Similarly, in an I/R injury‐induced acute lung injury mouse model, mitochondria from mouse gastrocnemius muscle administered via pulmonary artery injection or nebulization showed significant improvements in lung mechanics and reduced tissue injury [310]. Studies exploring innovative delivery methods have shown promising results, including iron oxide nanoparticle use to enhance mitochondrial transfer efficiency in a bleomycin‐induced pulmonary fibrosis mouse model [311]. Additionally, in LPS‐induced acute lung injury models, research showed that bone marrow stromal cells successfully transferred mitochondria via connexin 43 gap junctions [174]. Recent studies have also shown the effectiveness of exosome‐mediated mitochondrial transfer and mitochondrial transcription factor A (TFAM)‐mediated mitochondrial transfer from MSCs in improving alveolar macrophage function and endothelial barrier integrity [183, 221].

In a STZ‐induced diabetic nephropathy rat model, bone marrow‐derived stromal cells demonstrated improved proximal tubular epithelial cellular morphology and tubular structure restoration [188]. Studies using mouse models showed that mitochondrial transfer from MSCs to macrophages could suppress inflammatory responses and ameliorate kidney injury through PGC‐1α activation [200]. Research has shown that direct renal cortex injection of MSC‐derived mitochondria effectively mitigates doxorubicin‐induced nephrotoxicity by reducing oxidative stress, promoting tubular regeneration, and improving kidney function [222]. Similarly, intra‐arterial mitochondrial transplantation in porcine and rat models of I/R injury showed improved renal function and reduced inflammatory responses [219, 313]. Recent studies utilizing both bone marrow‐ and placenta‐derived MSC‐EVs demonstrated effectiveness in protecting against kidney injury through various mechanisms, including mtDNA stabilization and Keap1–Nrf2 signaling pathway activation [314, 315].

L6 rat myoblast cell‐derived mitochondrial transplantation in a rat model of tendinopathy reduced inflammatory markers and restored collagen production [316]. Studies on hindlimb I/R injury models showed that direct mitochondrial injection significantly reduced muscle infarct area and improved limb function recovery [317]. Research in muscle injury and atrophy models has shown promising results. Studies using BaCl_2_‐induced muscle injury models demonstrated enhanced muscle regeneration through both direct mitochondrial transplantation and mitochondria‐transferred myoblasts [224, 318]. In dexamethasone‐induced muscle atrophy, human umbilical cord‐derived MSC‐mitochondria significantly increased muscle mass and reduced atrophy markers [223]. The application of mitochondrial transplantation has extended to osteoarthritis and bone regeneration. In a monosodium iodoacetate‐induced osteoarthritis model, mitochondrial transplantation ameliorated pain and reduced cartilage destruction [319]. Studies in calvarial defect models demonstrated enhanced bone regeneration through both MSC‐derived mitochondria and osteoblast‐derived mitochondrial EVs [184, 225].

In glioma research, mitochondrial transplantation from normal human astrocytes via intratumor injection effectively inhibited xenograft tumor growth and enhanced radiosensitivity in nude mouse models [235]. Similarly, human cardiac fibroblast cell‐derived mitochondria showed enhanced chemotherapeutic sensitivity in prostate and ovarian cancer xenograft models [15]. In breast cancer models, particularly triple‐negative breast cancer, mitochondrial transplantation has shown significant therapeutic effects. Studies using membrane‐fused mitochondria with Pep‐1 conjugation (P‐Mito) demonstrated reduced tumor weight and marked Ki67 staining and angiogenesis inhibition. The treatment attenuated oxidative stress, decreased cancer‐associated fibroblast numbers, and promoted immune infiltration [324]. Further research revealed that P‐Mito increased doxorubicin sensitivity by promoting mitochondrial fusion and mitophagy, leading to p53‐mediated cell death [325]. In melanoma models, the intravenous injection of mouse liver‐derived mitochondria proved effective in both subcutaneous and metastatic lung models by reducing tumor volume and weight, accompanied by decreased pyruvate and ATP levels, leading to increased cell apoptosis [237]. Additional studies showed reduced glycolysis and glutaminolysis in tumor cells, increased ROS levels and oxidative stress, and enhanced tumor protein 53 levels, ultimately inducing tumor cell death through both apoptosis and necrosis [326]. Research on hepatocellular carcinoma demonstrated that intravenous mitochondrial transplantation effectively suppressed tumor proliferation, triggered apoptosis, and decreased aerobic glycolysis. The mechanism involved cell cycle protein downregulation and apoptosis‐related protein upregulation [239]. In conclusion, the consistency of positive outcomes across different cancer models, including glioma, breast cancer, melanoma, and hepatocellular carcinoma, suggests broad applicability of mitochondrial transplantation. The studies also reveal that transplanted mitochondria can influence both direct tumor cell death and the tumor microenvironment, including effects on cancer‐associated fibroblasts and immune cell infiltration, pointing to a multifaceted therapeutic mechanism.

### 4.2 In Vitro Studies of Mitochondrial Transplantation for Treating Disorder or Injury Models

The successful transplantation of mitochondria from external sources into various recipient cells has been demonstrated in numerous in vitro disease and injury models (Table 2). Techniques utilized for transplanting exogenous mitochondria include direct microinjection [327], coculture [328], mitoception [329], magnetomitotransfer [330], cell‐penetrating peptide delivery [331], dextran‐triphenylphosphonium (TPP) delivery [332], photothermal nanoblade [333], MitoPunch [334], mitochondria‐containing EV delivery, and fluidic force microscope (FluidFM) use [335, 336].

**TABLE 2:** In vitro studies of mitochondrial transplantation for treating disorder or injury models.

In vitro model of disease or injury	Source of mitochondria	Route of transplantation	Recipient	Outcome	References
In vitro cell models to simulate mitochondrial dysfunction	Synaptosomes extracted from rat cerebral cortex	Cocultivation	LAN5 cells treated with CCCP or rotenone	Restored the mitochondrial membrane potential; Increased the expression of mitochondria‐specific proteins; detected the presence of rat mitochondrial DNA	[337]
In vitro oxidative injury model to simulate cerebral ischemia	Human MSCs	Cocultivation	Primary cortical neurons from mice	Restored mitochondrial membrane potential; increased expression of mitochondria‐specific proteins; detection of rat mitochondrial DNA presence	[338]
MPP+‐treated SH‐SY5Y cells (a model for PD)	HepG2	Cocultivation	SH‐SY5Y cells	Significantly increased cell viability, respiratory complex I activity, ATP content, and glutathione level, while decreasing ROS level, apoptosis, and necrosis	[175]
In vitro H/R model to simulate I/R	Platelets of the human whole blood	Cocultivation	SH‐SY5Y cells	Prevented H/R‐induced mitochondrial malfunction and mitochondria‐mediated apoptosis; reduced mitochondrial ROS production; decreased cytochrome *C* leakage and enhanced cell viability; inhibited the H/R‐induced mitochondrial apoptotic pathway	[212]
Primary cortical neuron OGD model	Rat cortical astrocytes	Cocultivation	Primary cortical neurons from rats	Increased intracellular ATP levels in neurons; improved neuronal survival rate	[178]
OGD/reoxygenation‐induced injury model of neuronal cells	Rat astrocytes	Cocultivation	Primary cortical neurons from rats	Increased intracellular ATP levels in neurons; enhanced cell viability; reduced neuronal death rate	[339]
N2a cells H/R model	Mitochondria isolated from N2a cells	Cocultivation	N2a cells	Promoted mitochondrial fusion; reduced oxidative stress and apoptosis; increased cell viability	[213]
Primary cortical neuron OGD model	BHK‐21 cells	Cocultivation	Primary cortical neurons from rats	Improved neuronal survival; reduced neuronal injury	[186]
Mechanical injury model of rat hippocampal neurons	Healthy rat cortical neurons	Cocultivation	Injured hippocampal neurons	Neurite regeneration enhanced; membrane potential restored	[268]
Rotenone‐induced PD in vitro model	Human iPSC‐derived astrocytes	Cocultivation	Injured dopaminergic neurons derived from human iPSCs after exposure to rotenone	Significantly reversed dopaminergic neuronal degeneration and axonal pruning, restoring neuronal ATP levels and oxygen consumption rates	[340]
In vitro model of cisplatin‐induced neurotoxicity	MSCs derived from mice	Cocultivation	Mouse cortical NSCs	Restoration of mitochondrial membrane potential in NSCs; increased survival of NSCs; improvement of mitochondrial function in NSCs	[292]
In vitro model of cisplatin‐induced neurotoxicity	Rat astrocytes	Cocultivation	Primary cortical neurons from rats	Significantly increased the survival rate of cisplatin‐treated neurons; restored mitochondrial membrane potential; normalized calcium dynamics	[341]
In vitro model of scratch injury simulating mechanical injury caused by controlled cortical impact	Allogeneic mouse liver and autologous mouse muscle	Cocultivation	Primary cultured neurons, astrocytes, microglia, and oligodendrocytes in mice	Reduced neuronal apoptosis; TUNEL‐positive cells decreased from 70 to 50%	[180]
Schizophrenia‐derived iPSCs	Schizophrenia‐derived lymphoblasts and differentiating iPSCs	Cocultivation	Schizophrenia‐derived lymphoblasts and differentiating iPSCs	Long‐lasting enhancement in mitochondrial functions; improved differentiation efficiency of iPSCs into neurons	[216]
In vitro model of inflammatory response induced by sepsis	Pectoralis major muscle of mouse	Cocultivation	BV2 murine microglial cells and HT22 hippocampal cells	Mitochondrial transplantation enhanced mitochondrial content and function in microglia, promoted the conversion of microglia from M1 to M2 phenotype, and increased the survival rate of HT22 hippocampal cells.	[202]
Sciatic nerve crush injury model	BHK‐21 cells	Cocultivation	Rat sciatic nerve cultured in vitro	Reduced cytoskeletal loss and oxidative stress in isolated nerve explants, enhancing the potential for nerve regeneration	[172]
In vitro OGD injury model	Primary rat bone marrow MSCs	Cocultivation	Ventral spinal cord motor neurons or primary cortical neurons from rat embryos	Improved the bioenergetics profile of neurons; increased ATP content, reduced lactate dehydrogenase leakage and apoptosis, and promoted neuron survival; increased neurotrophic factor expression; restored the integrity of nerves and muscles	[287]
H_2_O_2_ or doxorubicin induced oxidative stress model	Cardiomyocytes and endothelial cells	Cocultivation	MSCs	Enhanced the ability of injured cells to counteract oxidative stress injury; enhanced MSC capacity to donate their own mitochondria to injured cells	[342]
In vitro model of simulated I/R	Bone marrow‐derived MSCs	Cocultivation	H9c2 cardiomyocytes	Increased resistance to I/R‐induced apoptosis	[343]
In vitro cell models to simulate pulmonary fibrosis	Human MSCs	Cocultivation	Mouse alveolar epithelial cells	Restoration of cell mitochondrial function; reduced oxidative stress; increased cell viability	[311]​
In vitro wound healing model	Human platelets	Cocultivation	Human dermal fibroblasts	Enhanced wound healing through increased fibroblast viability and reduced intracellular and mitochondrial ROS levels	[344]
H_2_O_2_‐induced oxidative stress model	Human platelets	Cocultivation	HUVECs	Reduced apoptosis	[345]
In vitro cell models to simulate inflammation	Human umbilical cord‐derived MSCs	Cocultivation	Human macrophages	Inhibited the production of proinflammatory cytokines and reduced inflammation	[201]
In vitro cell models to simulate immune disease	Human umbilical cord MSCs	Mitoception	Human peripheral blood mononuclear cells	Increased the expression of mRNA transcripts involved in T‐cell activation and T regulatory cell differentiation; expanded CD25^+^ FoxP3^+^ suppressive population	[323]
Sepsis/endotoxemia model induced by LPS stimulation	Rat liver	Direct exposure of purified mitochondria	Rat polymorphonuclear leukocytes (mainly neutrophils)	Purified mitochondria directly activate polymorphonuclear leukocytes, as shown by interleukin‐1β induction, a proinflammatory cytokine	[346]
In vitro tendinopathy model	Human umbilical cord MSCs	Centrifugation	Human tenocytes	Restoration of Tenomodulin and collagen I expression in injured tenocytes; attenuation of NF‐κB and matrix metalloproteinase 1 levels; dose‐dependent reduction in mitochondrial fission markers; recovery of apoptotic signaling proteins to baseline levels	[316]
In vitro muscle injury model	C2C12 myoblasts	Droplet microfluidics‐based mitochondrial transfer technique	C2C12 myoblasts	Enhanced myogenic differentiation; increased ATP production capacity	[318]
In vitro osteoarthritis model	L6 myoblast cell line	Cocultivation	Human osteoarthritis chondrocytes	Improved mitochondrial function; decreased inflammatory cell death	[319]
In vitro cell models to simulate diabetic nephropathy	Rat bone marrow‐derived stromal cells	Cocultivation	Proximal tubular epithelial cells	Suppressed apoptosis of impaired proximal tubular epithelial cells; increased expression of mitochondrial superoxide dismutase 2 and Bcl‐2; inhibited ROS production	[188]
In vitro cell models to simulate diabetic nephropathy	Mouse bone marrow MSCs	Cocultivation	Mouse macrophages	Enhanced mitochondrial transfer to macrophages; improved mitochondrial function and bioenergy; activation of PGC‐1α‐mediated mitochondrial biogenesis; increased lysosome‐autophagy for mitochondrial quality control; enhanced capacity to combat inflammatory response	[200]
Gentamicin‐induced nephrotoxicity model	Rat bone marrow MSCs	Cocultivation	Rat renal proximal tubular cells	Reduction in cytotoxicity and oxidative stress; decrease in ROS production, mitochondrial membrane potential collapse, lipid peroxidation content, oxidized glutathione levels, and caspase‐3 activity; increase in ATP and glutathione levels	[347]
Favipiravir‐induced nephrotoxicity model	Rat kidneys	Cocultivation	Rat renal proximal tubular cells	Reduction in cytotoxicity; decreased oxidative damage; mitigation of lysosomal damage; lowered apoptotic markers; improved energy homeostasis; enhanced antioxidant defense; increased antiapoptotic factors; elevated Bcl‐2 levels	[348]
Cisplatin‐induced nephrotoxicity	Rat kidneys	Cocultivation	Rat renal proximal tubular cells	Reduction in oxidative stress; decreased mitochondrial damage; mitigation of lysosomal membrane damage; lowered apoptotic activity; improved antioxidant capacity; enhanced protective efficacy in females; potential for nephrotoxicity treatment	[349]
Acute kidney injury in vitro model	Undamaged human conditionally immortalized proximal tubular cells	Cocultivation	Damaged human conditionally immortalized proximal tubular cells	Increased ATP production levels; enhanced the proliferative capacity; reduced cytotoxicity, including lower expression levels of apoptosis markers such as caspase‐3 and SOD; reduced ROS production; restored mitochondrial membrane potential	[312]
In vitro glioma model	Normal human astrocytes	Cocultivation	Human glioma cells	Enhanced tricarboxylic acid cycle gene and protein expression; increased aerobic respiration, reduced glycolysis rate; activated mitochondrial apoptotic pathway; inhibited malignant proliferation of human glioma cells	[235]
In vitro prostate cancer and ovarian cancer model	Human cardiac fibroblast cells	Cocultivation	DU145, PC3 prostate cancer cells and SKOV3 ovarian cancer cells	No effect on cancer cell proliferation; decreased cell migration; altered cell cycle checkpoints; enhanced chemotherapeutic sensitivity	[15]
In vitro breast cancer model	Homeoplasmic 143B osteosarcoma cybrids	Cell‐penetrating peptide delivery	MCF‐7 breast cancer cells	Enhanced cell apoptosis; inhibited cancer cell proliferation; decreased oxidative stress in MCF‐7 cells; enhanced chemosensitivity to doxorubicin and paclitaxel	[236]
In vitro breast cancer model	Mammary epithelial MCF‐12A cells	Cocultivation	MCF‐7 breast cancer cells	Inhibited cancer cell proliferation; enhanced chemosensitivity to doxorubicin, abraxane, and carboplatin	[238]
In vitro melanoma model	Mouse liver	Direct addition of isolated mitochondria to cell culture medium	Murine melanoma B16 cells	Decreased levels of pyruvate, lactate, and ATP; inhibited in vitro tumor cell proliferation through cell cycle arrest and cell apoptosis induction	[237]
In vitro hepatocellular carcinoma model	Mouse liver	Direct addition of isolated mitochondria to cell culture medium	Hepatocellular carcinoma H22 cell line	Reduced tumor proliferation; cell cycle arrest and apoptosis induction; decreased aerobic glycolysis; downregulation of cell cycle proteins; upregulation of apoptosis‐related proteins	[239]

*Abbreviations*: ATP, adenosine triphosphate; Bcl‐2, B‐cell lymphoma 2; BHK, baby hamster kidney fibroblasts; CCCP, carbonyl cyanide 3‐chlorophenylhydrazone; DNA, deoxyribonucleic acid; H/R, hypoxia/reoxygenation; HepG2, human hepatoma cell line; HUVECs, human umbilical vein endothelial cells; I/R, ischemia/reperfusion; iPSCs, induced pluripotent stem cells; LAN5, human neuroblastoma LAN5 cell line; LPS, lipopolysaccharide; MPP+, 1‐methyl‐4‐phenylpyridinium; mRNA, messenger ribonucleic acid; MSCs, mesenchymal stem cells; N2a, Neuro‐2a; NF‐κB, nuclear factor kappa‐B; NSCs, neural stem cells; OGD, oxygen–glucose deprivation; PD, Parkinson's disease; PGC‐1α, peroxisome proliferator‐activated receptor gamma coactivator 1‐alpha; ROS, reactive oxygen species; SH‐SY5Y, human neuroblastoma SH‐SY5Y cell line; SOD, superoxide dismutase.

**FIGURE 4:**
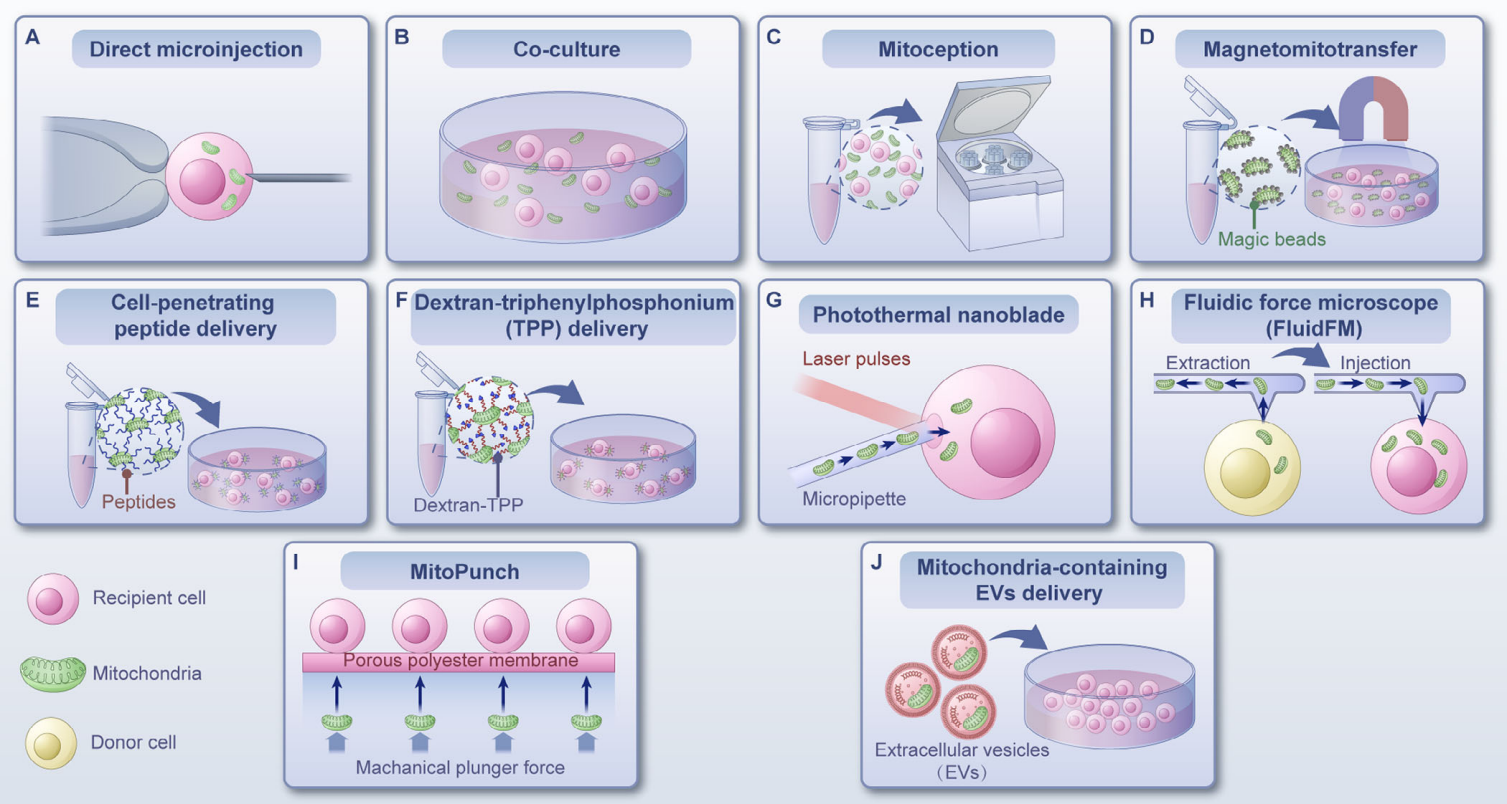
Methods for the transplantation of exogenous mitochondria into recipient cells. (A) Direct microinjection; (B) coculture; (C) mitoception; (D) magnetomitotransfer; (E) cell‐penetrating peptide delivery; (F) dextran‐triphenylphosphonium (TPP) delivery; (G) photothermal nanoblade; (H) fluidic force microscope (FluidFM); (I) MitoPunch; and (J) mitochondria‐containing extracellular vesicles (EVs) delivery.

Direct microinjection involves directly microinjecting isolated mitochondria into recipient cells, offering high accuracy but low efficiency [327] (Figure 4A). Coculture involves coincubating in vitro isolated mitochondria with recipient cells to promote mitochondrial transfer to the recipient cells [328] (Figure 4B). Mitoception involves centrifuging mitochondria with recipient cells, utilizing centrifugal force to transfer mitochondria into the recipient cells, thereby promoting mitochondrial uptake [328, 329, 350] (Figure 4C). Magnetomitotransfer applies an external magnetic field to attract magnetic bead‐bound mitochondria toward recipient cells to enhance mitochondrial uptake efficiency [330]. However, this approach may introduce dysfunctional mitochondria into the recipient cells (Figure 4D). Cell‐penetrating peptide delivery involves conjugating cell‐penetrating peptides with mitochondria, which can enhance mitochondrial uptake by recipient cells [331] (Figure 4E). Dextran‐TPP delivery involves coating mitochondria with dextran‐TPP, effectively protecting their respiratory function and facilitating their uptake by recipient cells [332] (Figure 4F). Photothermal nanoblade transfers isolated mitochondria into cells using a titanium‐coated micropipette [333]. This micropipette utilizes the photothermal effect of laser pulses to temporarily perforate the cell membrane, thereby facilitating mitochondrial entry into the cell (Figure 4G). FluidFM is an emerging technology that efficiently extracts, injects, and transplants mitochondria between single living cells, preserving their structure and function by directly extracting them from donor cells and injecting into recipient cells, thus avoiding complex isolation processes and preventing damage from prolonged exposure to nonphysiological conditions [335, 336] (Figure 4H). MitoPunch is a pressure‐driven mitochondrial transfer device that uses the driving force of a mechanical plunger to transfer mitochondria into recipient cells seeded on a porous polyester membrane [334] (Figure 4I). Mitochondria‐containing EV delivery uses EVs to deliver mitochondria, improving mitochondrial integrity and function in recipient cells [293] (Figure 4J).

Mitochondrial transplantation methods have evolved significantly, with various strategies developed to enhance mitochondrial uptake and function in recipient cells. Direct microinjection and coculture laid the groundwork for mitochondrial transplantation, while new techniques like cell‐penetrating peptides, MitoPunch, and FluidFM offer innovative approaches with varying degrees of efficiency and applicability. EV use presents a promising avenue for in vivo applications, highlighting the potential for therapeutic interventions in mitochondrial disorders. While selecting mitochondrial transplantation strategies, the prioritization of targeting cells is primarily dictated by the technical characteristics, delivery efficiency, and cellular biological properties of each method. Direct microinjection is renowned for its high precision, and although its delivery efficiency is relatively low, it offers significant advantages for cells with complex morphologies and specific functional requirements [327]. Coculture and mitoception provide moderate delivery efficiency, utilizing natural intercellular transfer mechanisms suitable for nonspecific targeting of mixed cell populations [328, 329, 350]. Magnetomitotransfer exhibits high delivery efficiency, achieving potential targeting of specific cell types through magnetic field modulation [330]. Cell‐penetrating peptide delivery leverages the flexibility of designing specific peptide sequences to enhance targeting affinity for cells while maintaining high delivery efficiency [331]. Dextran‐TPP delivery, known for its mitochondrial respiratory function protection and good delivery efficiency, is particularly suitable for cells with high energy demands [332]. Photothermal nanoblades and FluidFM technology offer high‐precision operations, applicable to cellular studies requiring precise manipulation, although their delivery efficiency may be limited by operational complexity [333, 335, 336]. The mechanical properties of MitoPunch enable effective mitochondrial delivery in cells [334]. Mitochondria‐containing EVs are also capable of facilitating effective mitochondrial delivery to cells [293]. Studies have demonstrated that EVs are crucial components of intercellular communication [351, 352].

In conclusion, comprehensive consideration of target cell biological characteristics, experimental objectives, delivery efficiency, and required specificity and precision is crucial in selecting the appropriate delivery method. With the deepening understanding of mitochondrial dynamics and intercellular transfer mechanisms, mitochondrial transplantation is emerging as a promising multifunctional therapeutic strategy. In various in vitro disease and injury models, mitochondrial transplantation has demonstrated significant therapeutic efficacy, providing a solid foundation for developing novel therapeutic approaches (Table 2).

Studies using rat cerebral cortex‐derived synaptosomes, subcellular fractions of isolated synaptic terminals containing mitochondria, as mitochondrial delivery systems, demonstrated that mitochondrial transplantation into CCCP‐ or rotenone‐treated human neuroblastoma LAN5 cells successfully restored mitochondrial membrane potential and increased mitochondria‐specific protein expression [337]. Similarly, MSC‐derived mitochondria cocultivated with primary cortical neurons from mice restored mitochondrial membrane potential and enhanced mitochondrial protein expression [338]. In models of PD, mitochondrial transplantation from HepG2 cells to 1‐methyl‐4‐phenylpyridinium‐treated human neuroblastoma SH‐SY5Y cells improved cell viability, respiratory complex I activity, ATP production, and antioxidant levels, while reducing ROS, apoptosis, and necrosis [175]. Platelets from human whole blood also demonstrated efficacy in reducing mitochondrial dysfunction and apoptosis in SH‐SY5Y cells subjected to H/R, enhancing cellular viability [212]. Primary cortical neurons subjected to oxygen–glucose deprivation (OGD) injury benefited from astrocyte‐derived mitochondria, exhibiting increased ATP levels and survival rates [178]. Similarly, mitochondrial transplantation into reoxygenated neurons in mild hypothermia conditions improved mitochondrial function, cell viability, and survival rates [339]. N2a cells undergoing H/R injury showed reduced oxidative stress and apoptosis following the transplantation of isolated mitochondria from N2a cells, leading to enhanced cell viability [213]. Mitochondria derived from baby hamster kidney fibroblasts cells enhanced survival and reduced neuronal injury in primary cortical neurons exposed to OGD [186]. Injured rat hippocampal neurons exhibited significant recovery in membrane potential and neurite regeneration upon transplantation with healthy rat cortical neuron mitochondria [227]. Furthermore, mitochondrial transplantation also showed therapeutic benefits in cisplatin‐induced neurotoxicity models. MSC‐derived mitochondria restored mitochondrial potential and increased survival in mouse cortical neural stem cells [292]. Similarly, rat astrocyte‐derived mitochondria improved survival and normalized calcium dynamics in cisplatin‐treated primary cortical neurons [341]. In mechanical injury models simulating controlled cortical impact, mitochondrial transplantation significantly reduced neuronal apoptosis [180]. For SZ models, mitochondria from SZ‐derived lymphoblasts enhanced differentiation efficiency into neurons and improved mitochondrial function [216]. Mitochondrial transplantation into isolated sciatic nerves reduced cytoskeletal loss and oxidative stress, promoting nerve regeneration [172]. Last, in OGD‐injured ventral spinal cord motor neurons or cortical neurons, transplantation with primary rat bone marrow MSC‐mitochondria enhanced bioenergetics, reduced oxidative stress, increased neurotrophic factors, and restored structural integrity [287]. In summary, mitochondrial transplantation demonstrates significant therapeutic potential in treating neurological disorders by addressing mitochondrial dysfunction, reducing oxidative stress, and enhancing cellular survival and repair mechanisms. Across diverse in vitro models, including PD, I/R injury, neuroinflammation, mechanical injury, and drug‐induced neurotoxicity, mitochondrial transplantation effectively restores mitochondrial membrane potential, increases ATP production, and reduces neuronal apoptosis. These studies highlight the versatility and efficacy of mitochondrial transplantation in enhancing cellular energy metabolism, promoting neuronal regeneration, and mitigating injury‐induced damage.

In breast cancer models, mitochondrial transplantation from homeoplasmic 143B osteosarcoma cybrids using cell‐penetrating peptide delivery into MCF‐7 breast cancer cells promoted cancer cell apoptosis, inhibited proliferation, reduced oxidative stress, and increased chemosensitivity to doxorubicin and paclitaxel [236]. Similarly, mitochondria derived from mammary epithelial MCF‐12A cells inhibited MCF‐7 breast cancer cell proliferation and enhanced their chemosensitivity to doxorubicin, abraxane, and carboplatin during cocultivation [238]. In an in vitro glioma model, mitochondrial transplantation from normal human astrocytes to human glioma cells activated the mitochondrial apoptotic pathway and inhibited the malignant proliferation of glioma cells [235]. In a prostate and ovarian cancer model, mitochondria from human cardiac fibroblast cells cocultivated with DU145, PC3 prostate cancer cells and SKOV3 ovarian cancer cells had no effect on proliferation but significantly decreased cell migration, altered cell cycle checkpoints, and enhanced chemotherapeutic sensitivity [15]. Mitochondrial transplantation from mouse liver was studied in melanoma and hepatocellular carcinoma models using direct addition of isolated mitochondria to the cell culture medium. In murine melanoma B16 cells, mitochondrial transplantation decreased pyruvate, lactate, and ATP levels, leading to tumor cell cycle arrest and apoptosis induction [237]. Similarly, mitochondrial transplantation reduced tumor proliferation, arrested the cell cycle, induced apoptosis, decreased aerobic glycolysis, downregulated cell cycle proteins, and upregulated apoptosis‐related proteins in hepatocellular carcinoma H22 cells [239]. In brief, mitochondrial transplantation effectively inhibits tumor proliferation, alters cell cycle checkpoints, and restores mitochondrial function in various in vitro cancer models, highlighting its potential to target metabolic vulnerabilities in cancer cells. Furthermore, mitochondrial transplantation potentiates chemotherapy efficacy through enhanced tumor cell sensitivity to agents including doxorubicin, paclitaxel, and carboplatin. This evidence positions mitochondrial transplantation as a complementary therapeutic strategy for cancers, paving the way for further exploration in preclinical and clinical settings.

Studies utilizing H_2_O_2_ or doxorubicin‐induced oxidative stress models demonstrated that mitochondrial transfer from cardiomyocytes and endothelial cells enhanced the therapeutic capacity of MSCs to counteract oxidative damage [342]. This finding was complemented by research showing that bone marrow‐derived MSCs could transfer mitochondria to H9c2 cardiomyocytes, significantly increasing their resistance to I/R‐induced apoptosis [343]. These studies collectively suggest that mitochondrial transfer could be a viable strategy for cardiac protection. In pulmonary fibrosis models, human MSCs restored mitochondrial function in mouse alveolar epithelial cells, leading to reduced oxidative stress and enhanced cell viability [311]. This finding suggests potential therapeutic applications in some respiratory conditions where mitochondrial dysfunction plays a central role.

In the context of trauma and wound healing, mitochondrial transfer has shown remarkable efficacy. Human platelet‐derived mitochondria significantly enhanced wound healing through multiple mechanisms, including increased fibroblast viability and reduced intracellular and mitochondrial ROS levels [344]. Furthermore, studies demonstrated that platelet‐derived mitochondria could protect human umbilical vein endothelial cells from oxidative stress‐induced apoptosis [345], suggesting broad applications in vascular repair and regeneration.

The role of mitochondrial transfer in modulating inflammatory and immune responses has been extensively studied. Human umbilical cord‐derived MSCs inhibit proinflammatory cytokine production through NF‐κB signaling pathway modulation in human macrophages [201]. Additionally, mitochondrial transfer via mitoception promoted T regulatory cell differentiation and expanded the immunosuppressive CD25^+^ FoxP3^+^ cell population [323]. In sepsis models, purified mitochondria from rat liver showed direct activation of polymorphonuclear leukocytes, as evidenced by IL‐1β induction [346], highlighting the complex role of mitochondria in immune regulation. In an in vitro sepsis‐induced inflammation model, mitochondrial transplantation from mouse pectoralis major muscle increased mitochondrial content, shifted microglial phenotypes to an anti‐inflammatory state, and improved hippocampal cell survival [202].

Perhaps the most extensive research has been conducted in renal disease models, with studies spanning various pathological conditions. In diabetic nephropathy models, rat bone marrow‐derived stromal cells effectively suppressed apoptosis in proximal tubular epithelial cells while increasing mitochondrial SOD 2 and Bcl‐2 expression [188]. Mouse bone marrow MSCs showed enhanced mitochondrial transfer to macrophages, improving mitochondrial function through PGC‐1α‐mediated biogenesis and enhanced lysosome‐autophagy for mitochondrial quality control [200]. Studies on nephrotoxicity have been particularly illuminating. In gentamicin‐induced models, mitochondrial transfer reduced cytotoxicity and oxidative stress while improving ATP and glutathione levels [347]. Similar protective effects were observed in favipiravir‐induced nephrotoxicity models, with improvements in energy homeostasis and antioxidant defense [348]. Cisplatin‐induced nephrotoxicity studies revealed gender‐specific responses, with enhanced protective efficacy observed in females [349]. In acute kidney injury models, mitochondrial transplantation demonstrated comprehensive therapeutic effects, including increased ATP production, enhanced proliferative capacity, and reduced ROS production [312].

The collective evidence from these in vitro studies demonstrates that mitochondrial transplantation represents a versatile and promising therapeutic approach across multiple disease models. Mitochondrial transfer can be achieved using diverse donor sources, such as MSCs and platelets, each showing unique advantages in specific therapeutic contexts. However, while these in vitro studies provide a strong foundation for understanding the therapeutic potential of mitochondrial transplantation, further research is needed to optimize delivery methods, determine ideal donor sources for specific conditions, and establish standardized protocols for clinical applications of mitochondrial transplantation.

## 5 Clinical Trials of Mitochondrial Transplantation

The promising outcomes of mitochondrial transplantation in diverse disease models have catalyzed the initiation of several human clinical trials. Researchers have carefully designed clinical trials to assess the safety, feasibility, and potential efficacy of mitochondrial transplantation in treating mitochondrial dysfunction‐associated human diseases.

Emani et al. [353] conducted a groundbreaking clinical trial on mitochondrial transplantation, focusing on pediatric patients suffering from I/R injury while on extracorporeal membrane oxygenation (ECMO). This study addresses a critical issue in cardiovascular medicine, as myocardial I/R injury significantly contributes to adverse outcomes after cardiac ischemia, surgery, or circulatory arrest. The researchers isolated autologous mitochondria from patient skeletal muscle and suspended them in a respiration buffer. These mitochondria were then rapidly administered via direct epicardial injection into the affected myocardium. This novel approach aimed to restore cellular function and mitigate damage in oxygen‐deprived cardiac tissue. The results were encouraging: all patients exhibited improved ventricular function within days posttransplantation. Notably, four out of five of the patients were successfully weaned off ECMO, although long‐term survival was observed in three cases. The absence of systemic inflammatory response syndrome markers before and after the procedure underscored the safety and efficacy of this autologous mitochondrial transplantation technique [353].

A clinical trial sponsored by Minovia Therapeutics Ltd. focuses on evaluating the safety and therapeutic effects of MNV–BM–BLD in pediatric patients with Pearson syndrome (NCT03384420). Pearson syndrome is a rare pediatric disease caused by mutations or deletions in mtDNA, typically presenting as a multisystem disease that can lead to death [354]. This study aims to alleviate symptoms of mitochondrial diseases through transplanting autologous CD34^+^ cells enriched with healthy mitochondria. Specifically, this involves enriching the peripheral hematopoietic stem cells with normal mitochondria derived from donor blood cells, a process known as mitochondrial augmentation therapy (MNV‐BM‐BLD). The primary outcome measures include the incidence of treatment‐related adverse events. Additionally, it evaluates changes in the International Pediatric Mitochondrial Disease Scale (IPMDS). This study could lay the foundation for the application of mitochondrial transplantation technology in Pearson syndrome and other mitochondrial diseases.

A notable human trial in South Korea, sponsored by Paean Biotechnology Inc., is investigating the safety, tolerability, and efficacy of PN‐101 transplantation in patients with refractory polymyositis or dermatomyositis (NCT04976140). This trial explores the use of mitochondria (PN‐101) isolated from allogeneic umbilical cord‐derived MSCs. The clinical trial employs a dose‐escalation design to identify the maximum tolerated dose of PN‐101. The mitochondria are administered intravenously, and the primary outcome measures include dose‐limiting toxicity assessment and improvements in the International Myositis Assessment and Clinical Studies Group‐total improvement score. Secondary outcomes focus on changes in core set activity measures, cutaneous dermatomyositis disease area and severity index, and peak pruritus numeral rating scale. The results of this trial could pave the way for the application of mitochondrial therapies in other inflammatory and degenerative diseases.

A clinical study conducted at Boston Children's Hospital explored the application of autologous mitochondrial transplantation in pediatric patients with cardiogenic shock requiring ECMO support due to I/R injury, aiming to assess its impact on myocardial function recovery [355]. The study compared patients undergoing revascularization followed by mitochondrial transplantation with a control group receiving revascularization alone. Results showed that 80% patients in the mitochondrial transplantation group successfully weaned off ECMO, compared with only 29% in the control group. Additionally, the time to ventricular function recovery was significantly short in the mitochondrial transplantation group (median: 2 days versus 9 days), and the incidence of cardiovascular events was significantly reduced (20 versus 79%). Safety assessments indicated that mitochondrial transplantation did not induce arrhythmias and was not associated with inflammatory or immune responses, with no significant differences in white blood cell count, lactate levels, or renal function indicators before and after treatment. The conclusion suggests that autologous mitochondrial transplantation may facilitate ECMO weaning and enhanced ventricular function [355]. This study provides preliminary evidence for the application of mitochondrial transplantation in myocardial I/R injury, indicating its potential as a therapeutic approach to improve outcomes in pediatric patients with cardiogenic shock.

The University of Washington is spearheading an innovative clinical trial involving autologous mitochondrial transplantation in patients with cerebral ischemia (NCT04998357). This groundbreaking study represents the first instance of mitochondrial transplantation being tested in patients with neurological disorders, marking it as a first‐in‐human brain clinical trial [356]. Researchers plan to infuse healthy autologous mitochondria into ischemic brain tissue via a microcatheter during standard endovascular reperfusion therapy. This study primarily aimed to evaluate the safety and tolerability of mitochondrial transplantation and observe its effects on ischemic brain injury. The findings of this research will provide critical safety data and preliminary efficacy assessments for the application of mitochondrial transplantation in treating cerebral ischemia.

The transition from animal studies to human clinical trials represents a significant milestone in mitochondrial transplantation study, offering hope for innovative treatments to address unmet medical needs. Although clinical trials of mitochondrial transplantation for patients with neurological disorders are still relatively scarce, they provide significant insights into the future application of mitochondrial transplantation in the CNS. By exploring the safety, feasibility, and preliminary efficacy of such interventions, these trials lay the groundwork for developing innovative therapies that could substantially impact the treatment and management of neurological diseases.

## 6 Mitochondrial Transplantation and Other Mitochondria‐Targeted Therapies

Mitochondria are fundamental cellular organelles critical for energy metabolism and cellular homeostasis. Accumulating evidence increasingly demonstrates a profound correlation between mitochondrial dysfunction and molecular mechanisms underlying diverse pathological conditions, including neurodegenerative disorders, metabolic syndromes, cardiovascular diseases, and certain malignancies [357, 358, 359, 360, 361]. The intricate relationship between mitochondrial impairment and disease progression is underpinned by multiple molecular pathways. Mitochondrial dysfunction can compromise cellular energy production, disrupt redox balance, trigger inflammatory responses, and initiate apoptotic cascades, which contribute significantly to cellular stress, tissue damage, and progressive pathological state development. Recognizing the crucial role of mitochondria in cell function and disease pathogenesis, researchers have increasingly viewed these organelles as a promising and sophisticated therapeutic target.

Mitochondria‐targeted antioxidants, including MitoQ and SkQ1, efficiently decrease mitochondrial ROS generation, mitigating excessive immune responses in animal models of sepsis [362]. These compounds are also promising in autoimmune conditions, including multiple sclerosis and TNF receptor‐associated periodic syndrome [363, 364]. Similarly, the restoration of mitochondrial function using antioxidants has been effective in reducing ROS production and improving insulin sensitivity in metabolic disorders such as insulin resistance [365, 366]. Furthermore, therapies targeting mitochondrial dysfunction and oxidative damage have shown potential in addressing hypertension, with mitochondria‐targeted antioxidants lowering blood pressure in preclinical models by mitigating endothelial oxidative stress and preserving nitric oxide bioavailability [367, 368]. Mitochondrial damage and oxidative stress are central to the progression of liver disease from steatosis to cirrhosis. Strategies such as enhancing mitochondrial fat oxidation with selective uncoupling agents or using mitochondria‐targeted antioxidants, including MitoQ and nicotinamide mononucleotide, have shown efficacy in preclinical nonalcoholic fatty liver disease models by reducing oxidative damage [369, 370]. MitoQ and mitochondria‐targeted peptide Bendavia (SS31) have shown protective effects in animal heart failure models, while coenzyme Q10 has improved heart function in clinical trials [371, 372, 373]. To address tissue damage caused by mitochondrial oxidative stress, researchers employ antioxidants like coenzyme Q10, idebenone, mitoquinone mesylate, and methylene blue for treating AD and ALS [374, 375, 376]. To combat mitochondrial injury and cellular homeostasis disruption due to excessive fission, inhibitors such as Mdivi‐1 are used in AD and PD therapy [377, 378]. To mitigate tissue damage resulting from insufficient mitochondrial production, drugs like triheptanoin and dynamin‐related protein antagonist 1 are utilized to enhance mitochondrial biogenesis in AD and HD management [379, 380]. Additionally, mitochondrial protective molecules, specifically the ketogenic compound d‐β‐hydroxybutyrate, are directly applied in treating AD, PD, and ALS [381, 382, 383, 384]. Last, the insulin sensitizer metformin is used to restore mitochondrial function and boost cerebral energy production in AD and HD treatment [385, 386].

These mitochondria‐focused therapeutic approaches open new avenues for treating a wide spectrum of diseases by addressing the underlying mechanisms of mitochondrial dysfunction. While several drugs, such as mitochondria‐targeted antioxidants and inhibitors of excessive mitochondrial fission, have shown promise in enhancing specific mitochondrial functions, their clinical translation faces significant challenges. Their effective delivery is hindered by biological barriers, including the plasma and mitochondrial membranes, which limit their bioavailability and efficacy [387, 388]. Most mitochondria‐targeted drugs require specialized delivery systems or conjugation with targeting moieties to overcome these obstacles and achieve sufficient accumulation within mitochondria [389]. Although these therapies often yield indirect benefits, such as reducing oxidative stress or modulating mitochondrial dynamics, they may not fully restore mitochondrial function in pathological conditions [374–376, 385]. This limitation underscores the need for further optimization of drug design and delivery strategies. Advancing these approaches requires not only a deeper understanding of mitochondrial biology, but also the development of innovative delivery systems to bypass cellular and mitochondrial barriers.

In contrast, mitochondrial transplantation offers an integrative therapeutic approach. By transferring healthy, functional mitochondria directly into damaged cells, this strategy can simultaneously enhance multiple mitochondrial functions, including bioenergetics, calcium homeostasis, and redox balance [170, 171, 191, 192, 230, 258]. Unlike pharmacological interventions that target isolated aspects of mitochondrial dysfunction, mitochondrial transplantation provides a more immediate and comprehensive cellular function restoration. Furthermore, this method holds promise for sustained therapeutic benefits, potentially offering longer‐term efficacy compared with conventional mitochondria‐targeted drugs. Continued research into optimizing mitochondrial delivery methods and ensuring compatibility with host tissues is essential for realizing the full potential of mitochondrial transplantation in clinical settings.

## 7 Challenges in Clinical Application of Mitochondrial Transplantation

### 7.1 Mitochondrial Isolation and Purification

The impact of the isolation process on mitochondrial structure and function should be evaluated when extracting mitochondria from cells or tissues. Studies have shown that this procedure can increase mitochondrial stress levels and stimulate free radical production [390]. Moreover, the isolation method may compromise OMM integrity, resulting in Cyt *c* leakage into the buffer solution, thereby affecting oxygen utilization and ATP synthesis [391]. It is noteworthy that certain mitochondrial components (such as mitochondrial proteins, N‐formyl peptides, mtDNA, or cardiolipin) often elicit significant immune responses after release into the extracellular environment [392, 393]. Consequently, protecting OMM proteins from damage during mitochondrial transplantation is of paramount importance, as they likely serve as ligands mediating interactions between mitochondria and recipient cells [394]. In conclusion, maintaining a high degree of integrity for candidate mitochondria is a crucial prerequisite for the successful implementation of mitochondrial transplantation.

Mitochondrial isolation methods are diverse, with differential centrifugation and filtration being popular due to their rapidity and high yield, albeit with lower purity [18, 395]. While these methods are simple and quick, often used for therapeutic mitochondrial transfer, they may also isolate nonmitochondrial particles, potentially causing adverse effects. Techniques like density gradient centrifugation and magnetic bead affinity purification can further refine mitochondria or separate specific subpopulations [18, 19]. Compared with differential centrifugation, these methods offer higher purity but lower yield [396]. However, time‐consuming isolation processes may reduce mitochondrial viability and transplantation success [171]. Flow cytometry‐based sorting significantly enhances mitochondrial purity and enables precise quantification [397]. Yet, this method requires more time to obtain sufficient mitochondria and specialized equipment. Thus, while suitable for biochemical analysis of pure mitochondria, it is suboptimal for clinical scenarios requiring rapid isolation of fresh mitochondria. Owing to these considerations, future research needs to concentrate on determining an appropriate and swift isolation method for mitochondrial transplantation.

### 7.2 Mitochondrial Storage

While conventional transplantation methods are effective in controlled laboratory settings, they may not be ideal in acute clinical scenarios due to the rapid degradation of mitochondria, likely caused by structural changes in their outer and inner membranes [123, 171]. McCully et al. [171] noted that isolated mitochondria maintain activity for only about 1 h on ice, with storage beyond this time significantly impacting transplantation efficiency. This limited storage duration constrains the clinical applications of mitochondrial transplantation. Improving mitochondrial preservation conditions to create storable preparations could greatly expand the therapeutic applications of mitochondrial transplantation. Cryopreservation of mitochondria might solve some cold storage issues. Earlier research found that mitochondria preserved at −65°C in 10% dimethyl sulfoxide retained complete respiratory function for 18 days [398]. However, subsequent investigations revealed limitations, with −80°C storage in identical cryoprotectant resulting in 20% functional decline within 7 days compared with fresh controls [399]. Although several studies have explored long‐term mitochondrial storage using cryoprotectants such as DMSO [400] and trehalose [401], these methods reduce mitochondrial functionality. Therefore, developing storage methods that maintain mitochondrial stability and functionality is crucial [402].

### 7.3 Rapid Validation of Mitochondrial Integrity and Functionality

Ensuring transplanted mitochondrial functionality and integrity represents a fundamental requirement for clinical translation of mitochondrial therapy. Consequently, rapid validation of mitochondrial integrity and function is crucial [20]. Current methods for assessing mitochondrial viability and function are time consuming and complex. Fluorescent probe staining, typically requiring 10–20 min, is commonly used to identify viable mitochondria [236]. Functional assessment often involves monitoring oxygen consumption rate using Clark‐type electrodes and measuring ATP production through luminescence assays, which are essential for evaluating respiratory efficiency and OXPHOS capacity [403]. Furthermore, additional time‐intensive analyses, including western blotting to detect specific contaminants from various cellular compartments and electron microscopy to examine mitochondrial structure and purity in detail, are necessary to ensure the purity of isolated mitochondria and exclude contamination from cytoplasmic and nuclear debris [403]. Developing rapid and accurate methods for evaluating mitochondrial integrity and functionality remains a significant challenge in the clinical application of mitochondrial transplantation. Overcoming this hurdle is essential for the successful translation of mitochondrial transplantation techniques from laboratory research to clinical practice.

### 7.4 Mitochondrial Targeted Delivery

A critical challenge in mitochondrial transplantation lies in achieving effective and precise delivery of viable mitochondria to diseased tissues, especially when targeting the CNS due to the restrictive blood–brain barrier (BBB). Enhancing the efficiency and specificity of mitochondrial transplantation, particularly in delivering healthy mitochondria to damaged neurons, remains a pressing issue. While current research paradigms in both animal models and clinical studies predominantly employ direct mitochondrial injection into target tissues [180, 186, 216, 230, 284], this approach may not be feasible in most clinical scenarios, particularly for CNS disorders such as ischemic strokes, TBI, and PD, where direct access is limited.

The development of tissue‐specific delivery systems is crucial, as different regions of the nervous system (such as brain, spinal cord, optic nerve) may have varying mitochondrial requirements. Such systems could enhance targeting and uptake in specific tissues. Additionally, determining the optimal source of mitochondria for transplantation into specific cell types is essential. Recent research has revealed intriguing insights into mitochondrial targeting. For instance, adipocyte‐derived mitochondria exhibit selective tropism toward adipose tissue‐resident macrophages [404]. Cellular heparan sulfates are potential receptors mediating the specific extracellular mitochondrial uptake by macrophages [405] and human HepG2 cells [394]. Furthermore, surface molecules/proteins on extracellularly secreted mitochondria may mediate recipient cell recognition, warranting systematic investigation. Characterization of these molecular signatures could enable mitochondrial modification approaches to enhance transplantation specificity while minimizing off‐target effects. Looking ahead, other optimized methods, such as utilizing BBB‐penetrating delivery systems [406] and employing MSCs loaded with mitochondria that can cross the BBB and provide therapeutic support for rescuing cells [292, 407], may offer additional therapeutic potential.

### 7.5 Potential Immune Responses

Mitochondrial transplantation, an emerging therapeutic approach, necessitates thorough evaluation of its efficacy and safety. Current studies presents conflicting evidence regarding the safety profile of this novel treatment modality. Some studies suggest that allogenic mitochondrial transplantation is well tolerated and does not provoke autoimmune responses [218]. Ramirez‐Barbieri et al. [408] examined immunological and damage‐associated molecular pattern (DAMP) responses in murine models following single or multiple intraperitoneal administrations of allogenic mitochondria and revealed no significant elevations in serum levels of IL‐2, interferon‐γ, or immunoglobulin M posttransplantation. Similarly, Kaza et al. [296] observed no notable increase in serum cytokine levels in a porcine model of I/R following a single autologous mitochondrial transplant. McCully et al. [408] demonstrated promising results from allogeneic mitochondrial injections for cardioprotection, with no remarkable posttransplant rise in cytokines, chemokines, or immunoglobulins. However, contradictory evidence has emerged, suggesting that allogeneic mitochondria may induce significant inflammatory responses, potentially linked to allograft dysfunction stemming from mitochondrial DAMP [409]. Zhang et al. [410] demonstrated that extracellular mitochondria can activate neutrophils, exerting immunostimulatory effects. Lin et al. [411] further elucidated that extracellular mitochondria can activate endothelial cells, increasing the production of cytokines and chemokines, key autoimmune activity mediators, thereby elevating alloreactive transplantation rejection risk. These conflicting findings pose a critical challenge to the safety of mitochondrial transplantation, particularly when involving systemic delivery of extracellular mitochondria to target organs.

The potential immunological response could eliminate exogenous mitochondria as the organism strives to maintain homeostasis [411]. To address this challenge, two promising approaches have been proposed. Preclinical evidence suggests autologous mitochondrial transplantation maintains stable immunity profiles [218], while EV‐mediated mitochondrial delivery offers superior biocompatibility over direct mitochondrial administration [412]. These strategies offer potential solutions to mitigate the immunological concerns associated with mitochondrial transplantation.

In conclusion, the immune response caused by transplanted mitochondria remains a significant hurdle. Further research is imperative to elucidate the potential immune responses to allogenic mitochondria and develop innovative solutions to mitigate these effects. Strategies such as immune modulatory approaches, autologous mitochondria utilization, or mitochondrial delivery via EVs warrant extensive investigation [403].

### 7.6 Long‐Term Efficacy and Safety

Mitochondrial transplantation, particularly allogeneic transplantation, faces several challenges impeding its clinical application. One primary concern is the potential impact of introducing foreign mtDNA into recipient cells. The cytoplasmic transmission of transplanted mitochondria carrying their own genetic material raises safety issues. The interaction between foreign mtDNA and the host nuclear genome may lead to mitochondrial genetic drift, potentially resulting in mitochondrial dysfunction [413]. The scientific community remains divided on the extent of this risk, with some researchers predicting adverse effects in human studies [414], while others argue for minimal risk [415]. This lack of consensus underscores the need for further research, which is currently ongoing [416]. The introduction of foreign DNA through mitochondrial transplantation also raises ethical considerations. Manipulating genetic material necessitates adherence to ethical guidelines and thorough ethical review processes. Moreover, allogeneic mitochondria may increase susceptibility to inflammation, oxidative stress, and heteroplasmy risks [17, 417].

A crucial aspect of mitochondrial transplantation is the integration and functionality of the transplanted organelles within the recipient cell. Research has demonstrated that exogenous mitochondria can fuse with endogenous counterparts upon transplantation [418]. This fusion process enables integration of transferred mitochondria into the recipient cellular mitochondrial network, enabling them to form functional connections with other organelles in the recipient cells [336]. However, the long‐term efficacy and safety of mitochondrial transplantation remain unclear. Key questions include how transplanted mitochondria integrate with the existing mitochondrial network in recipient cells and how they restore bioenergetic functions. This involves complex processes such as mitochondrial fusion, fission, and bioenergetic capability recovery.

**FIGURE 5:**
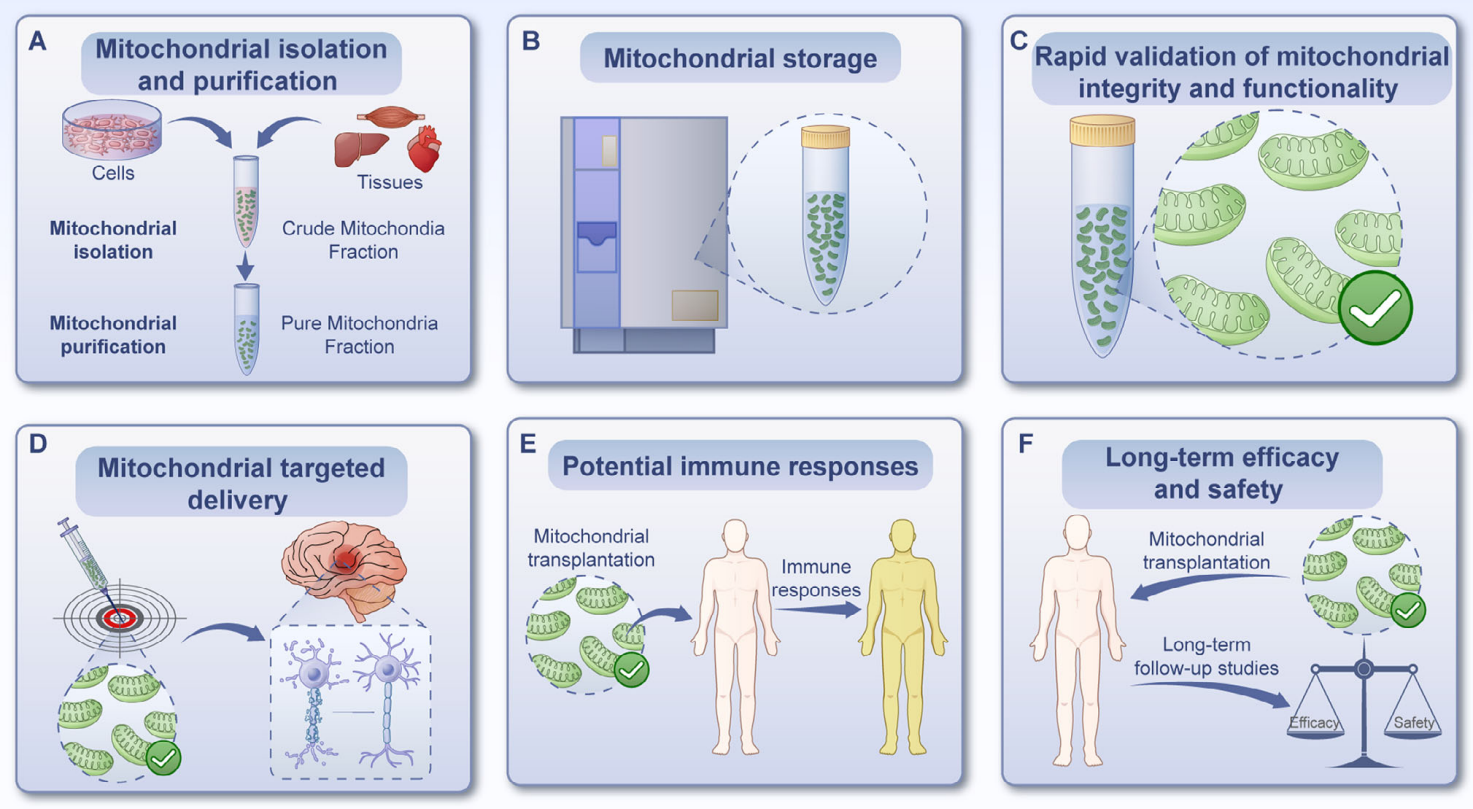
The major challenges faced by mitochondrial transplantation in the clinical application. (A) Mitochondrial isolation and purification; (B) mitochondrial storage; (C) rapid validation of mitochondrial integrity and functionality; (D) mitochondrial targeted delivery; (E) potential immune responses; and (F) long‐term efficacy and safety.

Additionally, the optimization of mitochondrial transplantation protocols remains a critical area for further research. This includes refining various aspects such as transplantation methodologies, optimal intervention timing, administration frequency, and appropriate dosage regimens. These factors require comprehensive investigation to establish standardized protocols for clinical application. Long‐term follow‐up studies are essential to address these uncertainties and evaluate the sustained therapeutic effects and safety profile of mitochondrial transplantation. The major challenges in advancing mitochondrial transplantation for clinical use in neurological disorders have been depicted in Figure 5.

## 8 Conclusion and Prospects

Mitochondrial transplantation has arisen as a hopeful therapeutic approach, demonstrating substantial potential for treating various disorders by restoring ATP production, attenuating oxidative stress, modulating inflammatory responses, reducing cellular apoptosis, promoting cell repair and regeneration, enhancing synaptic plasticity, increasing neurotrophic factor expression, facilitating neural circuit reconstruction, and exhibiting antitumor effects. These unique capabilities highlight its potential to address the complex pathophysiology of diseases involving multiple contributing factors.

Despite the encouraging results achieved in both in vivo and in vitro studies, the clinical translation of mitochondrial transplantation faces considerable challenges. Key hurdles include mitochondrial isolation and purification process optimization, safe storage protocols, targeted delivery systems, immune response mitigation, and long‐term safety and efficacy evaluation. Overcoming these obstacles is essential for translating the application of mitochondrial transplantation from experimental research to clinical practice. Moreover, the ability of mitochondrial transplantation to simultaneously target diverse pathological mechanisms distinguishes it from conventional single‐target therapies. This integrated approach to restoring cellular energetics and function holds significant promise for managing complex and multifactorial diseases. However, the field remains in its infancy, and comprehensive basic research and systematic preclinical studies are needed to elucidate its mechanisms and optimize therapeutic protocols.

In conclusion, mitochondrial transplantation represents a transformative frontier in treating human diseases. While considerable challenges remain, the compelling therapeutic potential of this approach underscores the need for continued rigorous research and technological development. Future efforts should prioritize refining mitochondrial transplantation methodologies, overcoming translational barriers, and conducting well‐designed clinical trials to establish safety and efficacy profiles. With sustained progress in these areas, mitochondrial transplantation is expected to become a valuable addition to the therapeutic armamentarium, offering profound benefits for patients with currently unmet medical needs.

## Author Contributions

Xinglu Miao drafted the manuscript and prepared the figures and tables. Pei Jiang revised the manuscript. Zhaoping Wang and Weihua Kong collected the related references. Lei Feng designed the study. All authors have read and approved the final manuscript.

## Conflicts of Interest

The authors declare no conflicts of interest.

## Ethics Statement

The authors have nothing to report.

## Data Availability

All data relevant to this review are included in the text, references, table, and figures.

## References

[1] D. C. Chan , “Mitochondria: Dynamic Organelles in Disease, Aging, and Development,” Cell 125, no. 7 (2006): 1241–1252. 16814712 10.1016/j.cell.2006.06.010

[2] C. Wang and R. J. Youle , “The Role of Mitochondria in Apoptosis,” Annual Review of Genetics 43 (2009): 95–118. 10.1146/annurev-genet-102108-134850 PMC4762029 19659442

[3] M. P. Bordone , M. M. Salman , H. E. Titus , et al., “The Energetic Brain—A Review From Students to Students,” Journal of Neurochemistry 151, no. 2 (2019): 139–165. 31318452 10.1111/jnc.14829

[4] M. R. Duchen , A. Verkhratsky , and S. Muallem , “Mitochondria and Calcium in Health and Disease,” Cell Calcium 44, no. 1 (2008): 1–5. 18378306 10.1016/j.ceca.2008.02.001

[5] W. F. Graier , M. Frieden , and R. Malli , “Mitochondria and Ca (2+) Signaling: Old Guests, New Functions,” Pflügers Archiv ‐ European Journal of Physiology 455, no. 3 (2007): 375–396. 17611770 10.1007/s00424-007-0296-1 PMC4864527

[6] W. Li , X. Liao , K. Lin , et al., “Earlier Second Polar Body Transfer and Further Mitochondrial Carryover Removal for Potential Mitochondrial Replacement Therapy,” MedComm 4, no. 3 (2023): e217. 37180823 10.1002/mco2.217 PMC10167372

[7] Y. Zong , H. Li , P. Liao , et al., “Mitochondrial Dysfunction: Mechanisms and Advances in Therapy,” Signal Transduction and Targeted Therapy 9, no. 1 (2024): 124. 38744846 10.1038/s41392-024-01839-8 PMC11094169

[8] J. S. Harrington , S. W. Ryter , M. Plataki , et al., “Mitochondria in Health, Disease, and Aging,” Physiological Reviews 103, no. 4 (2023): 2349–2422. 37021870 10.1152/physrev.00058.2021 PMC10393386

[9] A. Beckers and L. Moons , “Dendritic Shrinkage After Injury: A Cellular Killer or a Necessity for Axonal Regeneration?,” Neural Regeneration Research 14, no. 8 (2019): 1313. 30860164 10.4103/1673-5374.253505 PMC6524513

[10] F. Sayehmiri , F. Motamedi , Z. Batool , et al., “Mitochondrial Plasticity and Synaptic Plasticity Crosstalk; in Health and Alzheimer's Disease,” CNS Neuroscience & Therapeutics 30, no. 8 (2024): e14897. 39097920 10.1111/cns.14897 PMC11298206

[11] A. Mandal and C. M. Drerup , “Axonal Transport and Mitochondrial Function in Neurons,” Frontiers in Cellular Neuroscience 13 (2019): 373. 31447650 10.3389/fncel.2019.00373 PMC6696875

[12] K. A. Cojocaru , I. Luchian , A. Goriuc , et al., “Mitochondrial Dysfunction, Oxidative Stress, and Therapeutic Strategies in Diabetes, Obesity, and Cardiovascular Disease,” Antioxidants (Basel) 12, no. 3 (2023): 658. 36978905 10.3390/antiox12030658 PMC10045078

[13] Y. Zhang , L. Zhou , G. Cheng , et al., “Cordyceps sinensis Ameliorates Idiopathic Pulmonary Fibrosis in Mice via Inhibiting Mitochondrion‐mediated Oxidative Stress,” MedComm – Future Medicine 3, no. 3 (2024): e91.

[14] M. M. Khan , H. G. Paez , C. R. Pitzer , and S. E. Alway , “The Therapeutic Potential of Mitochondria Transplantation Therapy in Neurodegenerative and Neurovascular Disorders,” Current Neuropharmacology 21, no. 5 (2023): 1100–1116. 36089791 10.2174/1570159X05666220908100545 PMC10286589

[15] A. Celik , A. Orfany , J. Dearling , et al., “Mitochondrial Transplantation: Effects on Chemotherapy in Prostate and Ovarian Cancer Cells in Vitro and in Vivo,” Biomedicine & Pharmacotherapy 161 (2023): 114524. 36948134 10.1016/j.biopha.2023.114524

[16] B. Mokhtari , M. Hamidi , R. Badalzadeh , and A. Mahmoodpoor , “Mitochondrial Transplantation Protects Against sepsis‐induced Myocardial Dysfunction by Modulating Mitochondrial Biogenesis and Fission/Fusion and Inflammatory Response,” Molecular Biology Reports 50, no. 3 (2023): 2147–2158. 36565415 10.1007/s11033-022-08115-4

[17] J. H. Park , M. Tanaka , T. Nakano , et al., “O‐GlcNAcylation Is Essential for Therapeutic Mitochondrial Transplantation,” Communications Medicine 3, no. 1 (2023): 169. 38007588 10.1038/s43856-023-00402-w PMC10676354

[18] Y. Yin and H. Shen , “Common Methods in Mitochondrial Research (Review),” International Journal of Molecular Medicine 50, no. 4 (2022): 126. 36004457 10.3892/ijmm.2022.5182 PMC9448300

[19] W. B. Hubbard , C. L. Harwood , P. Prajapati , et al., “Fractionated Mitochondrial Magnetic Separation for Isolation of Synaptic Mitochondria From Brain Tissue,” Scientific Reports 9, no. 1 (2019): 9656. 31273236 10.1038/s41598-019-45568-3 PMC6609636

[20] R. Acin‐Perez , C. Benincá , B. Shabane , et al., “Utilization of Human Samples for Assessment of Mitochondrial Bioenergetics: Gold Standards, Limitations, and Future Perspectives,” Life 11, no. 9 (2021): 949. 34575097 10.3390/life11090949 PMC8467772

[21] M. Bonora , S. Patergnani , A. Rimessi , et al., “ATP Synthesis and Storage,” Purinergic Signal 8, no. 3 (2012): 343–357. 22528680 10.1007/s11302-012-9305-8 PMC3360099

[22] M. Rigoulet , C. L. Bouchez , P. Paumard , et al., “Cell Energy Metabolism: An Update,” Biochimica et Biophysica (BBA) ‐ Bioenergetics 1861, no. 11 (2020): 148276. 10.1016/j.bbabio.2020.148276 32717222

[23] S. A. Mookerjee , A. A. Gerencser , D. G. Nicholls , and M. D. Brand , “Quantifying Intracellular Rates of Glycolytic and Oxidative ATP Production and Consumption Using Extracellular Flux Measurements,” Journal of Biological Chemistry 292, no. 17 (2017): 7189–7207. 28270511 10.1074/jbc.M116.774471 PMC5409486

[24] P. J. Magistretti and I. Allaman , “Lactate in the Brain: From Metabolic End‐product to Signalling Molecule,” Nature Reviews Neuroscience 19, no. 4 (2018): 235–249. 29515192 10.1038/nrn.2018.19

[25] L. F. Barros , A. Brown , and R. A. Swanson , “Glia in Brain Energy Metabolism: A Perspective,” Glia 66, no. 6 (2018): 1134–1137. 29476554 10.1002/glia.23316 PMC6009848

[26] K. Tao , N. Matsuki , and R. Koyama , “AMP‐activated Protein Kinase Mediates Activity‐dependent Axon Branching by Recruiting Mitochondria to Axon,” Developmental Neurobiology 74, no. 6 (2014): 557–573. 24218086 10.1002/dneu.22149

[27] G. Ruthel and P. J. Hollenbeck , “Response of Mitochondrial Traffic to Axon Determination and Differential Branch Growth,” Journal of Neuroscience 23, no. 24 (2003): 8618–8624. 13679431 10.1523/JNEUROSCI.23-24-08618.2003 PMC6740379

[28] C. L. Zhang , P. L. Ho , D. B. Kintner , et al., “Activity‐dependent Regulation of Mitochondrial Motility by Calcium and Na/K‐ATPase at Nodes of Ranvier of Myelinated Nerves,” Journal of Neuroscience 30, no. 10 (2010): 3555–3566. 20219989 10.1523/JNEUROSCI.4551-09.2010 PMC3548432

[29] W. Dröge , “Free Radicals in the Physiological Control of Cell Function,” Physiological Reviews 82, no. 1 (2002): 47–95. 11773609 10.1152/physrev.00018.2001

[30] M. P. Murphy and R. C. Hartley , “Mitochondria as a Therapeutic Target for Common Pathologies,” Nat Rev Drug Discovery 17, no. 12 (2018): 865–886. 30393373 10.1038/nrd.2018.174

[31] L. A. J. O'Neill , R. J. Kishton , and J. Rathmell , “A Guide to Immunometabolism for Immunologists,” Nature Reviews Immunology 16, no. 9 (2016): 553–565. 10.1038/nri.2016.70 PMC5001910 27396447

[32] H. Tan , K. Yang , Y. Li , et al., “Integrative Proteomics and Phosphoproteomics Profiling Reveals Dynamic Signaling Networks and Bioenergetics Pathways Underlying T Cell Activation,” Immunity 46, no. 3 (2017): 488–503. 28285833 10.1016/j.immuni.2017.02.010 PMC5466820

[33] W. Y. Lam , A. M. Becker , K. M. Kennerly , et al., “Mitochondrial Pyruvate Import Promotes Long‐Term Survival of Antibody‐Secreting Plasma Cells,” Immunity 45, no. 1 (2016): 60–73. 27396958 10.1016/j.immuni.2016.06.011 PMC4956536

[34] E. Lachmandas , L. Boutens , J. M. Ratter , et al., “Microbial Stimulation of Different Toll‐Like Receptor Signalling Pathways Induces Diverse Metabolic Programmes in human Monocytes,” Nature microbiology 2 (2016): 16246. 10.1038/nmicrobiol.2016.246 27991883

[35] A. V. Kuznetsov , R. Margreiter , M. J. Ausserlechner , and J. Hagenbuchner , “The Complex Interplay Between Mitochondria, ROS and Entire Cellular Metabolism,” Antioxidants 11, no. 10 (2022): 1995. 36290718 10.3390/antiox11101995 PMC9598390

[36] Y. Liu , G. Fiskum , and D. Schubert , “Generation of Reactive Oxygen Species by the Mitochondrial Electron Transport Chain,” Journal of Neurochemistry 80, no. 5 (2002): 780–787. 11948241 10.1046/j.0022-3042.2002.00744.x

[37] Z. Zhao , “Iron and Oxidizing Species in Oxidative Stress and Alzheimer's Disease,” Aging Medicine Milton NSW 2, no. 2 (2019): 82–87. 10.1002/agm2.12074 PMC6880687 31942516

[38] A. Panov , “Perhydroxyl Radical (HO2•) as Inducer of the Isoprostane Lipid Peroxidation in Mitochondria,” Molecular Biology 52, no. 3 (2018): 295–305. 10.7868/S0026898418030011 29989570

[39] J. M. McCord and I. Fridovich , “Superoxide Dismutase. An Enzymic Function for Erythrocuprein (hemocuprein),” Journal of Biological Chemistry 244, no. 22 (1969): 6049–6055. 5389100

[40] R. A. Weisiger and I. Fridovich , “Superoxide Dismutase. Organelle Specificity,” Journal of Biological Chemistry 248, no. 10 (1973): 3582–3592. 4702877

[41] C. C. Winterbourn , “Toxicity of Iron and Hydrogen Peroxide: The Fenton Reaction,” Toxicology Letters 82‐83 (1995): 969–974. 10.1016/0378-4274(95)03532-x 8597169

[42] H. Sies and D. P. Jones , “Reactive Oxygen Species (ROS) as Pleiotropic Physiological Signalling Agents,” Nature Reviews Molecular Cell Biology 21, no. 7 (2020): 363–383. 32231263 10.1038/s41580-020-0230-3

[43] S. G. Rhee , “Cell Signaling. H2O2, a Necessary Evil for Cell Signaling,” Science 312, no. 5782 (2006): 1882–1883. 16809515 10.1126/science.1130481

[44] M. Schieber and N. S. Chandel , “ROS Function in Redox Signaling and Oxidative Stress,” Current Biology 24, no. 10 (2014): R453–R462. 24845678 10.1016/j.cub.2014.03.034 PMC4055301

[45] H. Sies , C. Berndt , and D. P. Jones , “Oxidative Stress,” Annual Review of Biochemistry 86 (2017): 715–748. 10.1146/annurev-biochem-061516-045037 28441057

[46] C. López‐Otín , M. A. Blasco , L. Partridge , et al., “The Hallmarks of Aging,” Cell 153, no. 6 (2013): 1194–1217. 23746838 10.1016/j.cell.2013.05.039 PMC3836174

[47] F. A. Court and M. P. Coleman , “Mitochondria as a central Sensor for Axonal Degenerative Stimuli,” Trends in Neuroscience (Tins) 35, no. 6 (2012): 364–372. 10.1016/j.tins.2012.04.001 22578891

[48] K. Biswas , K. Alexander , and M. M. Francis , “Reactive Oxygen Species: Angels and Demons in the Life of a Neuron,” NeuroScience 3, no. 1 (2022): 130–145. 10.3390/neurosci3010011 PMC11523706 39484669

[49] L. A. Sena and N. S. Chandel , “Physiological Roles of Mitochondrial Reactive Oxygen Species,” Molecular Cell 48, no. 2 (2012): 158–167. 23102266 10.1016/j.molcel.2012.09.025 PMC3484374

[50] A. Hervera , F. De Virgiliis , I. Palmisano , et al., “Reactive Oxygen Species Regulate Axonal Regeneration Through the Release of Exosomal NADPH Oxidase 2 Complexes Into Injured Axons,” Nature Cell Biology 20, no. 3 (2018): 307–319. 29434374 10.1038/s41556-018-0039-x

[51] M. C. Oswald , P. S. Brooks , M. F. Zwart , et al., “Reactive Oxygen Species Regulate Activity‐dependent Neuronal Plasticity in Drosophila,” Elife 7 (2018): e39393. 30540251 10.7554/eLife.39393 PMC6307858

[52] A. W. K. Yeung , N. T. Tzvetkov , M. G. Georgieva , et al., “Reactive Oxygen Species and Their Impact in Neurodegenerative Diseases: Literature Landscape Analysis,” Antioxid Redox Signaling 34, no. 5 (2021): 402–420. 10.1089/ars.2019.7952 32030995

[53] C. Wilson and C. González‐Billault , “Regulation of Cytoskeletal Dynamics by Redox Signaling and Oxidative Stress: Implications for Neuronal Development and Trafficking,” Frontiers in Cellular Neuroscience 9 (2015): 381. 26483635 10.3389/fncel.2015.00381 PMC4588006

[54] A. van der Pol , W. H. van Gilst , A. A. Voors , and P. van der Meer , “Treating Oxidative Stress in Heart Failure: Past, Present and Future,” European Journal of Heart Failure 21, no. 4 (2019): 425–435. 30338885 10.1002/ejhf.1320 PMC6607515

[55] C. E. Pinzón‐Díaz , J. V. Calderón‐Salinas , M. M. Rosas‐Flores , et al., “Eryptosis and Oxidative Damage in Hypertensive and Dyslipidemic Patients,” Molecular and Cellular Biochemistry 440, no. 1‐2 (2018): 105–113. 28822022 10.1007/s11010-017-3159-x

[56] M. A. Sánchez‐Rodríguez and V. M. Mendoza‐Núñez , “Oxidative Stress Indexes for Diagnosis of Health or Disease in Humans,” Oxidative Medicine and Cellular Longevity 2019, no. 1 (2019): 4128152. 31885788 10.1155/2019/4128152 PMC6899293

[57] A. Li , N. Zheng , and X. Ding , “Mitochondrial Abnormalities: A Hub in Metabolic Syndrome‐related Cardiac Dysfunction Caused by Oxidative Stress,” Heart Failure Reviews 27, no. 4 (2022): 1387–1394. 33950478 10.1007/s10741-021-10109-6 PMC9197868

[58] H. Tirichen , H. Yaigoub , W. Xu , et al., “Mitochondrial Reactive Oxygen Species and Their Contribution in Chronic Kidney Disease Progression through Oxidative Stress,” Frontiers in Physiology 12 (2021): 627837. 33967820 10.3389/fphys.2021.627837 PMC8103168

[59] Z. Geto , M. D. Molla , F. Challa , et al., “Mitochondrial Dynamic Dysfunction as a Main Triggering Factor for Inflammation Associated Chronic Non‐Communicable Diseases,” Journal of Inflammation Research 13 (2020): 97–107. 32110085 10.2147/JIR.S232009 PMC7034420

[60] V. Singh and S. Ubaid , “Role of Silent Information Regulator 1 (SIRT1) in Regulating Oxidative Stress and Inflammation,” Inflammation 43, no. 5 (2020): 1589–1598. 32410071 10.1007/s10753-020-01242-9

[61] S. Pitkanen and B. H. Robinson , “Mitochondrial Complex I Deficiency Leads to Increased Production of Superoxide Radicals and Induction of Superoxide Dismutase,” Journal of Clinical Investigation 98, no. 2 (1996): 345–351. 8755643 10.1172/JCI118798 PMC507436

[62] H. M. Ni , J. A. Williams , and W. X. Ding , “Mitochondrial Dynamics and Mitochondrial Quality Control,” Redox Biology 4 (2015): 6–13. 25479550 10.1016/j.redox.2014.11.006 PMC4309858

[63] M. Arai , H. Imai , T. Koumura , et al., “Mitochondrial Phospholipid Hydroperoxide Glutathione Peroxidase Plays a Major Role in Preventing Oxidative Injury to Cells,” Journal of Biological Chemistry 274, no. 8 (1999): 4924–4933. 9988735 10.1074/jbc.274.8.4924

[64] R. Rizzuto , A. W. Simpson , M. Brini , and T. Pozzan , “Rapid Changes of Mitochondrial Ca2+ Revealed by Specifically Targeted Recombinant Aequorin,” Nature 358, no. 6384 (1992): 325–327. 1322496 10.1038/358325a0

[65] E. D. Michelakis , “Mitochondrial Medicine: A New Era in Medicine Opens New Windows and Brings New Challenges,” Circulation 117, no. 19 (2008): 2431–2434. 18474822 10.1161/CIRCULATIONAHA.108.775163

[66] M. Colombini , “The VDAC Channel: Molecular Basis for Selectivity,” Biochimica Et Biophysica Acta 1863, no. 10 (2016): 2498–2502. 26826035 10.1016/j.bbamcr.2016.01.019

[67] D. De Stefani , A. Raffaello , E. Teardo , et al., “A Forty‐kilodalton Protein of the Inner Membrane Is the Mitochondrial Calcium Uniporter,” Nature 476, no. 7360 (2011): 336–340. 21685888 10.1038/nature10230 PMC4141877

[68] J. M. Baughman , F. Perocchi , H. S. Girgis , et al., “Integrative Genomics Identifies MCU as an Essential Component of the Mitochondrial Calcium Uniporter,” Nature 476, no. 7360 (2011): 341–345. 21685886 10.1038/nature10234 PMC3486726

[69] O. Kann and R. Kovacs , “Mitochondria and Neuronal Activity,” American Journal of Physiology‐Cell Physiology 292, no. 2 (2007): C641–C657. 17092996 10.1152/ajpcell.00222.2006

[70] D. Nicholls , “Mitochondria and Calcium Signaling,” Cell Calcium 38, no. 3‐4 (2005): 311–317. 16087232 10.1016/j.ceca.2005.06.011

[71] C. Picton , C. B. Klee , and P. Cohen , “The Regulation of Muscle Phosphorylase Kinase by Calcium Ions, Calmodulin and Troponin‐C,” Cell Calcium 2, no. 4 (1981): 281–294. 7200399 10.1016/0143-4160(81)90021-x

[72] J. G. McCormack , A. P. Halestrap , and R. M. Denton , “Role of Calcium Ions in Regulation of Mammalian Intramitochondrial Metabolism,” Physiological Reviews 70, no. 2 (1990): 391–425. 2157230 10.1152/physrev.1990.70.2.391

[73] M. S. Müller , “Functional Impact of Glycogen Degradation on Astrocytic Signalling,” Biochemical Society Transactions 42, no. 5 (2014): 1311–1315. 25233408 10.1042/BST20140157 PMC4179473

[74] L. S. Jouaville , P. Pinton , C. Bastianutto , et al., “Regulation of Mitochondrial ATP Synthesis by Calcium: Evidence for a Long‐term Metabolic Priming,” PNAS 96, no. 24 (1999): 13807–13812. 10570154 10.1073/pnas.96.24.13807 PMC24146

[75] R. Rizzuto , D. De Stefani , A. Raffaello , and C. Mammucari , “Mitochondria as Sensors and Regulators of Calcium Signalling,” Nature Reviews Molecular Cell Biology 13, no. 9 (2012): 566–578. 22850819 10.1038/nrm3412

[76] A. Herrero‐Mendez , A. Almeida , E. Fernández , et al., “The Bioenergetic and Antioxidant Status of Neurons Is Controlled by Continuous Degradation of a Key Glycolytic Enzyme by APC/C‐Cdh1,” Nature Cell Biology 11, no. 6 (2009): 747–752. 19448625 10.1038/ncb1881

[77] L. Tretter , K. Takacs , K. Kövér , and V. Adam‐Vizi , “Stimulation of H(2)O(2) Generation by Calcium in Brain Mitochondria Respiring on Alpha‐glycerophosphate,” Journal of Neuroscience Research 85, no. 15 (2007): 3471–3479. 17600838 10.1002/jnr.21405

[78] C. Mammucari , A. Raffaello , D. Vecellio Reane , et al., “Mitochondrial Calcium Uptake in Organ Physiology: From Molecular Mechanism to Animal Models,” Pflugers Archiv: European journal of physiology 470, no. 8 (2018): 1165–1179. 29541860 10.1007/s00424-018-2123-2 PMC6060757

[79] D. Boehning , R. L. Patterson , L. Sedaghat , et al., “Cytochrome c Binds to Inositol (1,4,5) Trisphosphate Receptors, Amplifying Calcium‐dependent Apoptosis,” Nature Cell Biology 5, no. 12 (2003): 1051–1061. 14608362 10.1038/ncb1063

[80] R. C. Reyes and V. Parpura , “Mitochondria Modulate Ca2+‐dependent Glutamate Release From Rat Cortical Astrocytes,” The Journal of Neuroscience 28, no. 39 (2008): 9682–9691. 18815254 10.1523/JNEUROSCI.3484-08.2008 PMC2614891

[81] K. S. Lee , S. Huh , S. Lee , et al., “Altered ER‐mitochondria Contact Impacts Mitochondria Calcium Homeostasis and Contributes to Neurodegeneration in Vivo in Disease Models,” PNAS 115, no. 38 (2018): E8844–E8853. 30185553 10.1073/pnas.1721136115 PMC6156612

[82] E. Zampese , C. Fasolato , T. Pozzan , and P. Pizzo , “Presenilin‐2 Modulation of ER‐mitochondria Interactions: FAD Mutations, Mechanisms and Pathological Consequences,” Communicative and Integrative Biology 4, no. 3 (2011): 357–360. 21980580 10.4161/cib.4.3.15160 PMC3187908

[83] A. Rossi , G. Rigotto , G. Valente , et al., “Defective Mitochondrial Pyruvate Flux Affects Cell Bioenergetics in Alzheimer's Disease‐Related Models,” Cell reports 30, no. 7 (2020): 2332–2348. e10. 32075767 10.1016/j.celrep.2020.01.060

[84] D. J. Surmeier , P. T. Schumacker , J. D. Guzman , et al., “Calcium and Parkinson's Disease,” Biochemical and Biophysical Research Communications 483, no. 4 (2017): 1013–1019. 27590583 10.1016/j.bbrc.2016.08.168 PMC5449761

[85] A. Agarwal , P. H. Wu , E. G. Hughes , et al., “Transient Opening of the Mitochondrial Permeability Transition Pore Induces Microdomain Calcium Transients in Astrocyte Processes,” Neuron 93, no. 3 (2017): 587–605. e7. 28132831 10.1016/j.neuron.2016.12.034 PMC5308886

[86] C. Giorgi , S. Marchi , and P. Pinton , “The Machineries, Regulation and Cellular Functions of Mitochondrial Calcium,” Nature Reviews Molecular Cell Biology 19, no. 11 (2018): 713–730. 30143745 10.1038/s41580-018-0052-8

[87] M. Bonora , M. R. Wieckowski , D. A. Sinclair , et al., “Targeting Mitochondria for Cardiovascular Disorders: Therapeutic Potential and Obstacles,” Nature Reviews Cardiology 16, no. 1 (2019): 33–55. 30177752 10.1038/s41569-018-0074-0 PMC6349394

[88] A. Danese , S. Patergnani , M. Bonora , et al., “Calcium Regulates Cell Death in Cancer: Roles of the Mitochondria and Mitochondria‐associated Membranes (MAMs),” Biochimica et Biophysica (BBA) ‐ Bioenergetics 1858, no. 8 (2017): 615–627. 10.1016/j.bbabio.2017.01.003 28087257

[89] S. Patergnani , A. Danese , E. Bouhamida , et al., “Various Aspects of Calcium Signaling in the Regulation of Apoptosis, Autophagy, Cell Proliferation, and Cancer,” International Journal of Molecular Sciences 21, no. 21 (2020): 8323. 33171939 10.3390/ijms21218323 PMC7664196

[90] C. Cardanho‐Ramos and V. A. Morais , “Mitochondrial Biogenesis in Neurons: How and Where,” International Journal of Molecular Sciences 22, no. 23 (2021): 13059. 34884861 10.3390/ijms222313059 PMC8657637

[91] R. C. Scarpulla , “Transcriptional Paradigms in Mammalian Mitochondrial Biogenesis and Function,” Physiological Reviews 88, no. 2 (2008): 611–638. 18391175 10.1152/physrev.00025.2007

[92] J. R. Friedman and J. Nunnari , “Mitochondrial Form and Function,” Nature 505, no. 7483 (2014): 335–343. 24429632 10.1038/nature12985 PMC4075653

[93] I. G. Onyango , J. Lu , M. Rodova , et al., “Regulation of Neuron Mitochondrial Biogenesis and Relevance to Brain Health,” Biochimica Et Biophysica Acta 1802, no. 1 (2010): 228–234. 19682571 10.1016/j.bbadis.2009.07.014

[94] M. Amiri and P. J. Hollenbeck , “Mitochondrial Biogenesis in the Axons of Vertebrate Peripheral Neurons,” Developmental Neurobiology 68, no. 11 (2008): 1348–1361. 18666204 10.1002/dneu.20668 PMC2538952

[95] A. Vaarmann , M. Mandel , A. Zeb , et al., “Mitochondrial Biogenesis Is Required for Axonal Growth,” Development (Cambridge, England) 143, no. 11 (2016): 1981–1992. 27122166 10.1242/dev.128926

[96] M. Golpich , E. Amini , Z. Mohamed , et al., “Mitochondrial Dysfunction and Biogenesis in Neurodegenerative Diseases: Pathogenesis and Treatment,” CNS Neuroscience & Therapeutics 23, no. 1 (2017): 5–22. 27873462 10.1111/cns.12655 PMC6492703

[97] S. Jamwal , J. K. Blackburn , and J. D. Elsworth , “PPARγ/PGC1α Signaling as a Potential Therapeutic Target for Mitochondrial Biogenesis in Neurodegenerative Disorders,” Pharmacology & Therapeutics 219 (2021): 107705. 33039420 10.1016/j.pharmthera.2020.107705 PMC7887032

[98] F. Ye and A. Wu , “The Protective Mechanism of SIRT1 in the Regulation of Mitochondrial Biogenesis and Mitochondrial Autophagy in Alzheimer's Disease,” Journal of Alzheimer's Disease 82, no. 1 (2021): 149–157. 10.3233/JAD-210132 33998544

[99] S. A. Murphy , M. Miyamoto , A. Kervadec , et al., “PGC1/PPAR Drive Cardiomyocyte Maturation at Single Cell Level via YAP1 and SF3B2,” Nature Communications 12, no. 1 (2021): 1648. 10.1038/s41467-021-21957-z PMC7955035 33712605

[100] H. Wang , W. J. Yan , J. L. Zhang , et al., “Adiponectin Partially Rescues High Glucose/High Fat‐induced Impairment of Mitochondrial Biogenesis and Function in a PGC‐1α Dependent Manner,” European Review for Medical and Pharmacological Sciences 21, no. 3 (2017): 590–599. 28239807

[101] X. Zhang , Z. Zhang , Y. Zhao , et al., “Alogliptin, a Dipeptidyl Peptidase‐4 Inhibitor, Alleviates Atrial Remodeling and Improves Mitochondrial Function and Biogenesis in Diabetic Rabbits,” Journal of the American Heart Association 6, no. 5 (2017): e005945. 28507060 10.1161/JAHA.117.005945 PMC5524117

[102] G. A. Oriquat , M. A. Ali , S. A. Mahmoud , et al., “Improving Hepatic Mitochondrial Biogenesis as a Postulated Mechanism for the Antidiabetic Effect of Spirulina platensis in Comparison With metformin,” Applied Physiology, Nutrition, and Metabolism 44, no. 4 (2019): 357–364. 10.1139/apnm-2018-0354 30208279

[103] V. S. LeBleu , J. T. O'Connell , K. N. G. Herrera , et al., “PGC‐1α Mediates Mitochondrial Biogenesis and Oxidative Phosphorylation to Promote Metastasis,” Nature Cell Biology 16, no. 10 (2014): 992–915. 10.1038/ncb3039 PMC4369153 25241037

[104] A. De Luca , M. Fiorillo , M. Peiris‐Pagès , et al., “Mitochondrial Biogenesis Is Required for the Anchorage‐independent Survival and Propagation of Stem‐Like Cancer Cells,” Oncotarget 6, no. 17 (2015): 14777–14795. 26087310 10.18632/oncotarget.4401 PMC4558115

[105] M. Gorska‐Ponikowska , A. Kuban‐Jankowska , S. A. Eisler , et al., “2‐Methoxyestradiol Affects Mitochondrial Biogenesis Pathway and Succinate Dehydrogenase Complex Flavoprotein Subunit A in Osteosarcoma Cancer Cells,” Cancer Genomics & Proteomics 15, no. 1 (2018): 73–89. 29275365 10.21873/cgp.20067 PMC5822178

[106] T. Wai and T. Langer , “Mitochondrial Dynamics and Metabolic Regulation,” Trends in Endocrinology & Metabolism 27, no. 2 (2016): 105–117. 26754340 10.1016/j.tem.2015.12.001

[107] D. C. Chan , “Mitochondrial Dynamics and Its Involvement in Disease,” Annual Review of Pathology 15 (2020): 235–259. 10.1146/annurev-pathmechdis-012419-032711 31585519

[108] K. R. Pitts , Y. Yoon , E. W. Krueger , and M. A. McNiven , “The Dynamin‐Like Protein DLP1 Is Essential for Normal Distribution and Morphology of the Endoplasmic Reticulum and Mitochondria in Mammalian Cells,” Molecular Biology of the Cell 10, no. 12 (1999): 4403–4417. 10588666 10.1091/mbc.10.12.4403 PMC25766

[109] E. Smirnova , L. Griparic , D. L. Shurland , and A. M. van der Bliek , “Dynamin‐related Protein Drp1 Is Required for Mitochondrial Division in Mammalian Cells,” Molecular Biology of the Cell 12, no. 8 (2001): 2245–2256. 11514614 10.1091/mbc.12.8.2245 PMC58592

[110] H. Otera , N. Miyata , O. Kuge , and K. Mihara , “Drp1‐dependent Mitochondrial Fission via MiD49/51 Is Essential for Apoptotic Cristae Remodeling,” Journal of Cell Biology 212, no. 5 (2016): 531–544. 26903540 10.1083/jcb.201508099 PMC4772499

[111] O. C. Losón , Z. Song , H. Chen , and D. C. Chan , “Fis1, Mff, MiD49, and MiD51 Mediate Drp1 Recruitment in Mitochondrial Fission,” Molecular Biology of the Cell 24, no. 5 (2013): 659–667. 23283981 10.1091/mbc.E12-10-0721 PMC3583668

[112] F. Kraus , K. Roy , T. J. Pucadyil , and M. T. Ryan , “Function and Regulation of the Divisome for Mitochondrial Fission,” Nature 590, no. 7844 (2021): 57–66. 33536648 10.1038/s41586-021-03214-x

[113] Z. Song , M. Ghochani , J. M. McCaffery , et al., “Mitofusins and OPA1 Mediate Sequential Steps in Mitochondrial Membrane Fusion,” Molecular Biology of the Cell 20, no. 15 (2009): 3525–3532. 19477917 10.1091/mbc.E09-03-0252 PMC2719570

[114] A. S. Rambold , B. Kostelecky , and J. Lippincott‐Schwartz , “Together We Are Stronger: Fusion Protects Mitochondria From Autophagosomal Degradation,” Autophagy 7, no. 12 (2011): 1568–1569. 22024745 10.4161/auto.7.12.17992 PMC3327623

[115] N. Zemirli , E. Morel , and D. Molino , “Mitochondrial Dynamics in Basal and Stressful Conditions,” International Journal of Molecular Sciences 19, no. 2 (2018): 564. 29438347 10.3390/ijms19020564 PMC5855786

[116] M. Giacomello , A. Pyakurel , C. Glytsou , and L. Scorrano , “The Cell Biology of Mitochondrial Membrane Dynamics,” Nature Reviews Molecular Cell Biology 21, no. 4 (2020): 204–224. 32071438 10.1038/s41580-020-0210-7

[117] D. Larrea , M. Pera , A. Gonnelli , et al., “MFN2 mutations in Charcot–Marie–Tooth Disease Alter Mitochondria‐associated ER Membrane Function but Do Not Impair Bioenergetics,” Human Molecular Genetics 28, no. 11 (2019): 1782–1800. 30649465 10.1093/hmg/ddz008 PMC6522073

[118] A. B. Knott , G. Perkins , R. Schwarzenbacher , and E. Bossy‐Wetzel , “Mitochondrial Fragmentation in Neurodegeneration,” Nature Reviews Neuroscience 9, no. 7 (2008): 505–518. 18568013 10.1038/nrn2417 PMC2711514

[119] M. Liesa , M. Palacin , and A. Zorzano , “Mitochondrial Dynamics in Mammalian Health and Disease,” Physiological Reviews 89, no. 3 (2009): 799–845. 19584314 10.1152/physrev.00030.2008

[120] D. F. Kashatus , “The Regulation of Tumor Cell Physiology by Mitochondrial Dynamics,” Biochemical and Biophysical Research Communications 500, no. 1 (2018): 9–16. 28676396 10.1016/j.bbrc.2017.06.192 PMC5748380

[121] J. Rehman , H. J. Zhang , P. T. Toth , et al., “Inhibition of Mitochondrial Fission Prevents Cell Cycle Progression in Lung Cancer,” The FASEB Journal 26, no. 5 (2012): 2175–2186. 22321727 10.1096/fj.11-196543 PMC3336787

[122] J. A. Kashatus , A. Nascimento , L. J. Myers , et al., “Erk2 phosphorylation of Drp1 Promotes Mitochondrial Fission and MAPK‐driven Tumor Growth,” Molecular Cell 57, no. 3 (2015): 537–551. 25658205 10.1016/j.molcel.2015.01.002 PMC4393013

[123] F. J. Bock and S. W. G. Tait , “Mitochondria as Multifaceted Regulators of Cell Death,” Nature Reviews Molecular Cell Biology 21, no. 2 (2020): 85–100. 31636403 10.1038/s41580-019-0173-8

[124] J. Song , J. M. Herrmann , and T. Becker , “Quality Control of the Mitochondrial Proteome,” Nature Reviews Molecular Cell Biology 22, no. 1 (2021): 54–70. 33093673 10.1038/s41580-020-00300-2

[125] A. Sugiura , G. L. McLelland , E. A. Fon , and H. M. McBride , “A New Pathway for Mitochondrial Quality Control: Mitochondrial‐derived Vesicles,” Embo Journal 33, no. 19 (2014): 2142–2156. 25107473 10.15252/embj.201488104 PMC4282503

[126] T. König , H. Nolte , M. J. Aaltonen , et al., “MIROs and DRP1 Drive Mitochondrial‐derived Vesicle Biogenesis and Promote Quality Control,” Nature Cell Biology 23, no. 12 (2021): 1271–1286. 34873283 10.1038/s41556-021-00798-4

[127] A. Picca , J. Faitg , J. Auwerx , et al., “Mitophagy in human Health, Ageing and Disease,” Nature Metabolism 5, no. 12 (2023): 2047–2061. 10.1038/s42255-023-00930-8 PMC12159423 38036770

[128] A. C. Poole , R. E. Thomas , L. A. Andrews , et al., “The PINK1/Parkin Pathway Regulates Mitochondrial Morphology,” PNAS 105, no. 5 (2008): 1638–1643. 18230723 10.1073/pnas.0709336105 PMC2234197

[129] I. E. Clark , M. W. Dodson , C. Jiang , et al., “Drosophila pink1 Is Required for Mitochondrial Function and Interacts Genetically With Parkin,” Nature 441, no. 7097 (2006): 1162–1166. 16672981 10.1038/nature04779

[130] E. M. Valente , P. M. Abou‐Sleiman , V. Caputo , et al., “Hereditary Early‐onset Parkinson's Disease Caused by Mutations in PINK1,” Science 304, no. 5674 (2004): 1158–1160. 15087508 10.1126/science.1096284

[131] T. Kitada , S. Asakawa , N. Hattori , et al., “Mutations in the Parkin Gene Cause Autosomal Recessive Juvenile Parkinsonism,” Nature 392, no. 6676 (1998): 605–608. 9560156 10.1038/33416

[132] S. A. Sarraf , M. Raman , V. Guarani‐Pereira , et al., “Landscape of the PARKIN‐dependent Ubiquitylome in Response to Mitochondrial Depolarization,” Nature 496, no. 7445 (2013): 372–376. 23503661 10.1038/nature12043 PMC3641819

[133] M. Lazarou , D. A. Sliter , L. A. Kane , et al., “The Ubiquitin Kinase PINK1 Recruits Autophagy Receptors to Induce Mitophagy,” Nature 524, no. 7565 (2015): 309–314. 26266977 10.1038/nature14893 PMC5018156

[134] M. Y. W. Ng , T. Wai , and A. Simonsen , “Quality Control of the Mitochondrion,” Developmental Cell 56, no. 7 (2021): 881–905. 33662258 10.1016/j.devcel.2021.02.009

[135] T. T. Ho , M. R. Warr , E. R. Adelman , et al., “Autophagy Maintains the Metabolism and Function of Young and Old Stem Cells,” Nature 543, no. 7644 (2017): 205–210. 28241143 10.1038/nature21388 PMC5344718

[136] M. N. Quinsay , R. L. Thomas , Y. Lee , and A. B. Gustafsson , “Bnip3‐mediated Mitochondrial Autophagy Is Independent of the Mitochondrial Permeability Transition Pore,” Autophagy 6, no. 7 (2010): 855–862. 20668412 10.4161/auto.6.7.13005 PMC3039735

[137] H. Shitara , H. Kaneda , A. Sato , et al., “Selective and Continuous Elimination of Mitochondria Microinjected Into Mouse Eggs From Spermatids, but Not From Liver Cells, Occurs throughout Embryogenesis,” Genetics 156, no. 3 (2000): 1277–1284. 11063701 10.1093/genetics/156.3.1277 PMC1461340

[138] S. M. Jin and R. J. Youle , “PINK1‐ and Parkin‐mediated Mitophagy at a Glance,” Journal of Cell Science 125, no. Pt 4 (2012): 795–799. 22448035 10.1242/jcs.093849 PMC3656616

[139] E. F. Fang , Y. Hou , K. Palikaras , et al., “Mitophagy Inhibits Amyloid‐β and Tau Pathology and Reverses Cognitive Deficits in Models of Alzheimer's Disease,” Nature Neuroscience 22, no. 3 (2019): 401–412. 30742114 10.1038/s41593-018-0332-9 PMC6693625

[140] S. Hwang , M. H. Disatnik , and D. Mochly‐Rosen , “Impaired GAPDH‐induced Mitophagy Contributes to the Pathology of Huntington's Disease,” EMBO Molecular Medicine 7, no. 10 (2015): 1307–1326. 26268247 10.15252/emmm.201505256 PMC4604685

[141] J. P. Bernardini , M. Lazarou , and G. Dewson , “Parkin and Mitophagy in Cancer,” Oncogene 36, no. 10 (2017): 1315–1327. 27593930 10.1038/onc.2016.302

[142] S. Ito , J. Araya , Y. Kurita , et al., “PARK2‐mediated Mitophagy Is Involved in Regulation of HBEC Senescence in COPD Pathogenesis,” Autophagy 11, no. 3 (2015): 547–559. 25714760 10.1080/15548627.2015.1017190 PMC4502689

[143] H. Bugger and K. Pfeil , “Mitochondrial ROS in Myocardial Ischemia Reperfusion and Remodeling,” Biochimica et Biophysica Acta (BBA) ‐ Molecular Basis of Disease 1866, no. 7 (2020): 165768. 32173461 10.1016/j.bbadis.2020.165768

[144] H. Pei , Y. Yang , H. Zhao , et al., “The Role of Mitochondrial Functional Proteins in ROS Production in Ischemic Heart Diseases,” Oxidative Medicine and Cellular Longevity 2016 (2016): 5470457. 27119006 10.1155/2016/5470457 PMC4826939

[145] L. Galluzzi , J. M. Bravo‐San Pedro , I. Vitale , et al., “Essential versus Accessory Aspects of Cell Death: Recommendations of the NCCD 2015,” Cell Death and Differentiation 22, no. 1 (2015): 58–73. 25236395 10.1038/cdd.2014.137 PMC4262782

[146] F. H. Igney and P. H. Krammer , “Death and Anti‐death: Tumour Resistance to Apoptosis,” Nature Reviews Cancer 2, no. 4 (2002): 277–288. 12001989 10.1038/nrc776

[147] S. Elmore , “Apoptosis: A Review of Programmed Cell Death,” Toxicologic Pathology 35, no. 4 (2007): 495–516. 17562483 10.1080/01926230701320337 PMC2117903

[148] D. R. Green and G. Kroemer , “The Pathophysiology of Mitochondrial Cell Death,” Science 305, no. 5684 (2004): 626–629. 15286356 10.1126/science.1099320

[149] H. L. Vieira and G. Kroemer , “Pathophysiology of Mitochondrial Cell Death Control,” Cellular and Molecular Life Sciences CMLS 56, no. 11‐12 (1999): 971–976. 11212328 10.1007/s000180050486 PMC11146840

[150] R. J. Youle and A. Strasser , “The BCL‐2 Protein family: Opposing Activities That Mediate Cell Death,” Nature Reviews Molecular Cell Biology 9, no. 1 (2008): 47–59. 18097445 10.1038/nrm2308

[151] C. Hockings , K. Anwari , R. L. Ninnis , et al., “Bid Chimeras Indicate That Most BH3‐only Proteins Can Directly Activate Bak and Bax, and Show no Preference for Bak versus Bax,” Cell Death & Disease 6, no. 4 (2015): e1735. 25906158 10.1038/cddis.2015.105 PMC4650538

[152] H. Zou , W. J. Henzel , X. Liu , et al., “Apaf‐1, a Human Protein Homologous to C. elegans CED‐4, Participates in Cytochrome c–Dependent Activation of Caspase‐3,” Cell 90, no. 3 (1997): 405–413. 9267021 10.1016/s0092-8674(00)80501-2

[153] P. Li , D. Nijhawan , I. Budihardjo , et al., “Cytochrome c and dATP‐Dependent Formation of Apaf‐1/Caspase‐9 Complex Initiates an Apoptotic Protease Cascade,” Cell 91, no. 4 (1997): 479–489. 9390557 10.1016/s0092-8674(00)80434-1

[154] X. Liu , C. N. Kim , J. Yang , et al., “Induction of Apoptotic Program in Cell‐Free Extracts: Requirement for dATP and Cytochrome c,” Cell 86, no. 1 (1996): 147–157. 8689682 10.1016/s0092-8674(00)80085-9

[155] K. N. Alavian , G. Beutner , E. Lazrove , et al., “An Uncoupling Channel Within the c‐subunit Ring of the F1FO ATP Synthase Is the Mitochondrial Permeability Transition Pore,” Pnas 111, no. 29 (2014): 10580–10585. 24979777 10.1073/pnas.1401591111 PMC4115574

[156] V. Giorgio , S. Von Stockum , M. Antoniel , et al., “Dimers of Mitochondrial ATP Synthase Form the Permeability Transition Pore,” Pnas 110, no. 15 (2013): 5887–5892. 23530243 10.1073/pnas.1217823110 PMC3625323

[157] N. Mnatsakanyan and E. A. Jonas , “The New Role of F1Fo ATP Synthase in Mitochondria‐mediated Neurodegeneration and Neuroprotection,” Experimental Neurology 332 (2020): 113400. 32653453 10.1016/j.expneurol.2020.113400 PMC7877222

[158] S. Orrenius , V. Gogvadze , and B. Zhivotovsky , “Calcium and Mitochondria in the Regulation of Cell Death,” Biochemical and Biophysical Research Communications 460, no. 1 (2015): 72–81. 25998735 10.1016/j.bbrc.2015.01.137

[159] C. P. Baines , R. A. Kaiser , N. H. Purcell , et al., “Loss of Cyclophilin D Reveals a Critical Role for Mitochondrial Permeability Transition in Cell Death,” Nature 434, no. 7033 (2005): 658–662. 15800627 10.1038/nature03434

[160] B. Fadeel and S. Orrenius , “Apoptosis: A Basic Biological Phenomenon With Wide‐ranging Implications in human Disease,” Journal of Internal Medicine 258, no. 6 (2005): 479–517. 16313474 10.1111/j.1365-2796.2005.01570.x

[161] N. Salvadores , M. Sanhueza , P. Manque , and F. A. Court , “Axonal Degeneration During Aging and Its Functional Role in Neurodegenerative Disorders,” Frontiers in Neuroscience 11 (2017): 451. 28928628 10.3389/fnins.2017.00451 PMC5591337

[162] S. Saxena and P. Caroni , “Mechanisms of Axon Degeneration: From Development to Disease,” Progress in Neurobiology 83, no. 3 (2007): 174–191. 17822833 10.1016/j.pneurobio.2007.07.007

[163] T. G. Cotter , “Apoptosis and Cancer: The Genesis of a Research Field,” Nature Reviews Cancer 9, no. 7 (2009): 501–507. 19550425 10.1038/nrc2663

[164] N. S. Erekat , “Apoptosis and Its Therapeutic Implications in Neurodegenerative Diseases,” Clinical Anatomy 35, no. 1 (2022): 65–78. 34558138 10.1002/ca.23792

[165] A. Roulston , R. C. Marcellus , and P. E. Branton , “Viruses and Apoptosis,” Annual Review of Microbiology 53 (1999): 577–628. 10.1146/annurev.micro.53.1.577 10547702

[166] N. W. Cummins and A. D. Badley , “Mechanisms of HIV‐associated Lymphocyte Apoptosis: 2010,” Cell Death & Disease 1, no. 11 (2010): e99. 21368875 10.1038/cddis.2010.77 PMC3032328

[167] G. Olivetti , F. Quaini , R. Sala , et al., “Acute Myocardial Infarction in Humans Is Associated With Activation of Programmed Myocyte Cell Death in the Surviving Portion of the Heart,” Journal of Molecular and Cellular Cardiology 28, no. 9 (1996): 2005–2016. 8899559 10.1006/jmcc.1996.0193

[168] A. Saraste , K. Pulkki , M. Kallajoki , et al., “Apoptosis in human Acute Myocardial Infarction,” Circulation 95, no. 2 (1997): 320–323. 9008443 10.1161/01.cir.95.2.320

[169] A. Abbate , G. G. L. Biondi‐Zoccai , R. Bussani , et al., “Increased Myocardial Apoptosis in Patients With Unfavorable Left Ventricular Remodeling and Early Symptomatic Post‐infarction Heart Failure,” Journal of the American College of Cardiology 41, no. 5 (2003): 753–760. 12628718 10.1016/s0735-1097(02)02959-5

[170] J. L. Gollihue and A. G. Rabchevsky , “Prospects for Therapeutic Mitochondrial Transplantation,” Mitochondrion 35 (2017): 70–79. 28533168 10.1016/j.mito.2017.05.007 PMC5518605

[171] J. D. McCully , D. B. Cowan , S. M. Emani , and P. J. Del Nido , “Mitochondrial Transplantation: From Animal Models to Clinical Use in Humans,” Mitochondrion 34 (2017): 127–134. 28342934 10.1016/j.mito.2017.03.004

[172] C. C. Kuo , H. L. Su , T. L. Chang , et al., “Prevention of Axonal Degeneration by Perineurium Injection of Mitochondria in a Sciatic Nerve Crush Injury Model,” Neurosurgery 80, no. 3 (2017): 475–488. 28362972 10.1093/neuros/nyw090

[173] K. Liu , L. Guo , Z. Zhou , et al., “Mesenchymal Stem Cells Transfer Mitochondria Into Cerebral Microvasculature and Promote Recovery From Ischemic Stroke,” Microvascular Research 123 (2019): 74–80. 30611747 10.1016/j.mvr.2019.01.001

[174] M. N. Islam , S. R. Das , M. T. Emin , et al., “Mitochondrial Transfer From Bone Marrow‐derived Stromal Cells to Pulmonary Alveoli Protects Against Acute Lung Injury,” Nature Medicine 18, no. 5 (2012): 759–765. 10.1038/nm.2736 PMC3727429 22504485

[175] X. Shi , M. Zhao , C. Fu , and A. Fu , “Intravenous Administration of Mitochondria for Treating Experimental Parkinson's Disease,” Mitochondrion 34 (2017): 91–100. 28242362 10.1016/j.mito.2017.02.005

[176] M. Vos , E. Lauwers , and P. Verstreken , “Synaptic Mitochondria in Synaptic Transmission and Organization of Vesicle Pools in Health and Disease,” Frontiers in Synaptic Neuroscience 2 (2010): 139. 21423525 10.3389/fnsyn.2010.00139 PMC3059669

[177] Y. Wang , J. Ni , C. Gao , et al., “Mitochondrial Transplantation Attenuates Lipopolysaccharide‐ induced Depression‐Like Behaviors,” Progress in Neuro‐Psychopharmacology & Biological Psychiatry 93 (2019): 240–249. 31022424 10.1016/j.pnpbp.2019.04.010

[178] K. Hayakawa , E. Esposito , X. Wang , et al., “Transfer of Mitochondria From Astrocytes to Neurons After Stroke,” Nature 535, no. 7613 (2016): 551–555. 27466127 10.1038/nature18928 PMC4968589

[179] Y. C. Chuang , C. W. Liou , S. D. Chen , et al., “Mitochondrial Transfer From Wharton's Jelly Mesenchymal Stem Cell to MERRF Cybrid Reduces Oxidative Stress and Improves Mitochondrial Bioenergetics,” Oxidative Medicine and Cellular Longevity 2017 (2017): 5691215. 28607632 10.1155/2017/5691215 PMC5457778

[180] J. Zhao , D. Qu , Z. Xi , et al., “Mitochondria Transplantation Protects Traumatic Brain Injury via Promoting Neuronal Survival and Astrocytic BDNF,” Translational Research Journal of Laboratory and Clinical Medicine 235 (2021): 102–114. 10.1016/j.trsl.2021.03.017 33798765

[181] Z. Zhang , Z. Ma , C. Yan , et al., “Muscle‐derived Autologous Mitochondrial Transplantation: A Novel Strategy for Treating Cerebral Ischemic Injury,” Behavioural Brain Research 356 (2019): 322–331. 30213662 10.1016/j.bbr.2018.09.005

[182] Z. Zhao , Y. Hou , W. Zhou , et al., “Mitochondrial Transplantation Therapy Inhibit Carbon Tetrachloride‐induced Liver Injury Through Scavenging Free Radicals and Protecting Hepatocytes,” Bioengineering & Translational Medicine 6, no. 2 (2021): e10209. 34027095 10.1002/btm2.10209 PMC8126821

[183] L. Xia , C. Zhang , N. Lv , et al., “AdMSC‐derived Exosomes Alleviate Acute Lung Injury via Transferring Mitochondrial Component to Improve Homeostasis of Alveolar Macrophages,” Theranostics 12, no. 6 (2022): 2928–2947. 35401830 10.7150/thno.69533 PMC8965475

[184] Y. Guo , X. Chi , Y. Wang , et al., “Mitochondria Transfer Enhances Proliferation, Migration, and Osteogenic Differentiation of Bone Marrow Mesenchymal Stem Cell and Promotes Bone Defect Healing,” Stem Cell Research and Therapy 11 (2020): 245. 32586355 10.1186/s13287-020-01704-9 PMC7318752

[185] C. Cornelius , R. Crupi , V. Calabrese , et al., “Traumatic Brain Injury: Oxidative Stress and Neuroprotection,” Antioxid Redox Signaling 19, no. 8 (2013): 836–853. 10.1089/ars.2012.4981 23547621

[186] P. J. Huang , C. C. Kuo , H. C. Lee , et al., “Transferring Xenogenic Mitochondria Provides Neural Protection against Ischemic Stress in Ischemic Rat Brains,” Cell Transplantation 25, no. 5 (2016): 913–927. 26555763 10.3727/096368915X689785

[187] M. W. Lin , S. Y. Fang , J. Y. C. Hsu , et al., “Mitochondrial Transplantation Attenuates Neural Damage and Improves Locomotor Function after Traumatic Spinal Cord Injury in Rats,” Frontiers in neuroscience 16 (2022): 800883. 35495036 10.3389/fnins.2022.800883 PMC9039257

[188] N. Konari , K. Nagaishi , S. Kikuchi , and M. Fujimiya , “Mitochondria Transfer From Mesenchymal Stem Cells Structurally and Functionally Repairs Renal Proximal Tubular Epithelial Cells in Diabetic Nephropathy in Vivo,” Scientific Reports 9 (2019): 5184. 30914727 10.1038/s41598-019-40163-y PMC6435708

[189] C. T. Madreiter‐Sokolowski , C. Thomas , and M. Ristow , “Interrelation Between ROS and Ca2+ in Aging and Age‐related Diseases,” Redox Biology 36 (2020): 101678. 32810740 10.1016/j.redox.2020.101678 PMC7451758

[190] E. Cadenas and A. Boveris , “Enhancement of Hydrogen Peroxide Formation by Protophores and Ionophores in Antimycin‐supplemented Mitochondria,” Biochemical Journal 188, no. 1 (1980): 31–37. 7406888 10.1042/bj1880031 PMC1162533

[191] A. Görlach , K. Bertram , S. Hudecova , and O. Krizanova , “Calcium and ROS: A Mutual Interplay,” Redox Biology 6 (2015): 260–271. 26296072 10.1016/j.redox.2015.08.010 PMC4556774

[192] Z. Liu , Y. Sun , Z. Qi , et al., “Mitochondrial Transfer/Transplantation: An Emerging Therapeutic Approach for Multiple Diseases,” Cell BioSciences 12, no. 1 (2022): 66. 10.1186/s13578-022-00805-7 PMC9121600 35590379

[193] B. Mokhtari , R. Yavari , R. Badalzadeh , and A. Mahmoodpoor , “An Overview on Mitochondrial‐Based Therapies in Sepsis‐Related Myocardial Dysfunction: Mitochondrial Transplantation as a Promising Approach,” Canadian Journal of Infectious Diseases and Medical Microbiology 2022 (2022): 3277274. 35706715 10.1155/2022/3277274 PMC9192296

[194] S. D. Skaper , L. Facci , M. Zusso , and P. Giusti , “An Inflammation‐Centric View of Neurological Disease: Beyond the Neuron,” Frontiers in Cellular Neuroscience 12 (2018): 72. 29618972 10.3389/fncel.2018.00072 PMC5871676

[195] S. Y. Fang , J. N. Roan , J. S. Lee , et al., “Transplantation of Viable Mitochondria Attenuates Neurologic Injury After Spinal Cord Ischemia,” Journal of Thoracic and Cardiovascular Surgery 161, no. 5 (2021): e337–e347. 31866084 10.1016/j.jtcvs.2019.10.151

[196] C. C. Huang , H. Y. Chiu , P. H. Lee , et al., “Mitochondrial Transplantation Attenuates Traumatic Neuropathic Pain, Neuroinflammation, and Apoptosis in Rats With Nerve Root Ligation,” Molecular Pain 19 (2023): 17448069231210423. 37845039 10.1177/17448069231210423 PMC10605811

[197] B. Zhang , C. Pan , C. Feng , et al., “Role of Mitochondrial Reactive Oxygen Species in Homeostasis Regulation,” Redox Report: Communications in Free Radical Research 27, no. 1 (2022): 45–52. 35213291 10.1080/13510002.2022.2046423 PMC8890532

[198] U. Kaur , P. Banerjee , A. Bir , et al., “Reactive Oxygen Species, Redox Signaling and Neuroinflammation in Alzheimer's Disease: The NF‐κB Connection,” Current Topics in Medicinal Chemistry 15, no. 5 (2015): 446–457. 25620241 10.2174/1568026615666150114160543

[199] D. S. A. Simpson and P. L. Oliver , “ROS Generation in Microglia: Understanding Oxidative Stress and Inflammation in Neurodegenerative Disease,” Antioxidants 9, no. 8 (2020): 743. 32823544 10.3390/antiox9080743 PMC7463655

[200] Y. Yuan , L. Yuan , L. Li , et al., “Mitochondrial Transfer From Mesenchymal Stem Cells to Macrophages Restricts Inflammation and Alleviates Kidney Injury in Diabetic Nephropathy Mice via PGC‐1α Activation,” Stem Cells (Dayton, Ohio) 39, no. 7 (2021): 913–928. 33739541 10.1002/stem.3375

[201] S. H. Yu , S. Kim , Y. Kim , et al., “Human Umbilical Cord Mesenchymal Stem Cell‐derived Mitochondria (PN‐101) Attenuate LPS‐induced Inflammatory Responses by Inhibiting NFκB Signaling Pathway,” BMB Reports 55, no. 3 (2022): 136–141. 34488927 10.5483/BMBRep.2022.55.3.083 PMC8972135

[202] C. Yan , Z. Ma , H. Ma , et al., “Mitochondrial Transplantation Attenuates Brain Dysfunction in Sepsis by Driving Microglial M2 Polarization,” Molecular Neurobiology 57, no. 9 (2020): 3875–3890. 32613465 10.1007/s12035-020-01994-3

[203] X. Jia , Q. Wang , J. Ji , et al., “Mitochondrial Transplantation Ameliorates Hippocampal Damage Following Status Epilepticus,” Animal Models and Experimental Medicine 6, no. 1 (2023): 41–50. 36734302 10.1002/ame2.12310 PMC9986225

[204] C. Bamshad , M. Habibi Roudkenar , M. Abedinzade , et al., “Human Umbilical Cord‐derived Mesenchymal Stem Cells‐harvested Mitochondrial Transplantation Improved Motor Function in TBI Models Through Rescuing Neuronal Cells From Apoptosis and Alleviating Astrogliosis and Microglia Activation,” International Immunopharmacology 118 (2023): 110106. 37015158 10.1016/j.intimp.2023.110106

[205] L. Galluzzi , O. Kepp , C. Trojel‐Hansen , and G. Kroemer , “Mitochondrial Control of Cellular Life, Stress, and Death,” Circulation Research 111, no. 9 (2012): 1198–1207. 23065343 10.1161/CIRCRESAHA.112.268946

[206] G. Kroemer and J. C. Reed , “Mitochondrial Control of Cell Death,” Nature Medicine 6, no. 5 (2000): 513–519. 10.1038/74994 10802706

[207] D. R. Green and J. C. Reed , “Mitochondria and Apoptosis,” Science 281, no. 5381 (1998): 1309–1312. 9721092 10.1126/science.281.5381.1309

[208] P. Norat , S. Soldozy , J. D. Sokolowski , et al., “Mitochondrial Dysfunction in Neurological Disorders: Exploring Mitochondrial Transplantation,” NPJ Regenerative Medicine 5, no. 1 (2020): 22. 33298971 10.1038/s41536-020-00107-x PMC7683736

[209] A. Park , M. Oh , S. J. Lee , et al., “Mitochondrial Transplantation as a Novel Therapeutic Strategy for Mitochondrial Diseases,” International Journal of Molecular Sciences 22, no. 9 (2021): 4793. 33946468 10.3390/ijms22094793 PMC8124982

[210] J. C. Chang , S. L. Wu , K. H. Liu , et al., “Allogeneic/Xenogeneic Transplantation of Peptide‐labeled Mitochondria in Parkinson's disease: Restoration of Mitochondria Functions and Attenuation of 6‐hydroxydopamine‐induced Neurotoxicity,” Translational Research Journal of Laboratory and Clinical Medicine 170 (2016): 40–56. e3. 10.1016/j.trsl.2015.12.003 26730494

[211] H. W. Hyatt and S. K. Powers , “Mitochondrial Dysfunction Is a Common Denominator Linking Skeletal Muscle Wasting due to Disease, Aging, and Prolonged Inactivity,” Antioxidants 10, no. 4 (2021): 588. 33920468 10.3390/antiox10040588 PMC8070615

[212] C. Shi , H. Guo , and X. Liu , “Platelet Mitochondria Transplantation Rescues Hypoxia/Reoxygenation‐Induced Mitochondrial Dysfunction and Neuronal Cell Death Involving the FUNDC2/PIP3/Akt/FOXO3a Axis,” Cell Transplantation 30 (2021): 9636897211024210. 34105393 10.1177/09636897211024210 PMC8193664

[213] Q. Xie , J. Zeng , Y. Zheng , et al., “Mitochondrial Transplantation Attenuates Cerebral Ischemia‐Reperfusion Injury: Possible Involvement of Mitochondrial Component Separation,” Oxidative Medicine and Cellular Longevity 2021 (2021): 1006636. 34849186 10.1155/2021/1006636 PMC8627565

[214] J. D. Ly , D. R. Grubb , and A. Lawen , “The Mitochondrial Membrane Potential (deltapsi(m)) in Apoptosis; an Update,” Apoptosis: A Review of Programmed Cell Death 8, no. 2 (2003): 115–128. 10.1023/a:1022945107762 12766472

[215] L. Hosseini , M. Karimipour , F. Seyedaghamiri , et al., “Intranasal Administration of Mitochondria Alleviated Cognitive Impairments and Mitochondrial Dysfunction in the Photothrombotic Model of mPFC Stroke in Mice,” Journal of Stroke and Cerebrovascular Diseases Off Journal National Stroke Association 31, no. 12 (2022): 106801. 10.1016/j.jstrokecerebrovasdis.2022.106801 36257142

[216] O. Robicsek , H. M. Ene , R. Karry , et al., “Isolated Mitochondria Transfer Improves Neuronal Differentiation of Schizophrenia‐Derived Induced Pluripotent Stem Cells and Rescues Deficits in a Rat Model of the Disorder,” Schizophrenia Bulletin 44, no. 2 (2018): 432–442. 28586483 10.1093/schbul/sbx077 PMC5814822

[217] V. Weixler , R. Lapusca , G. Grangl , et al., “Autogenous Mitochondria Transplantation for Treatment of Right Heart Failure,” Journal of Thoracic and Cardiovascular Surgery 162, no. 1 (2021): e111–e121. 32919774 10.1016/j.jtcvs.2020.08.011

[218] A. Masuzawa , K. M. Black , C. A. Pacak , et al., “Transplantation of Autologously Derived Mitochondria Protects the Heart From Ischemia‐reperfusion Injury,” American Journal of Physiology‐Heart and Circulatory Physiology 304, no. 7 (2013): H966–H982. 23355340 10.1152/ajpheart.00883.2012 PMC3625892

[219] H. Jabbari , A. M. Roushandeh , M. K. Rostami , et al., “Mitochondrial Transplantation Ameliorates Ischemia/Reperfusion‐induced Kidney Injury in Rat,” Biochimica et Biophysica Acta ‐ Molecular Basis of Disease 1866, no. 8 (2020): 165809. 32353613 10.1016/j.bbadis.2020.165809

[220] J. W. Hwang , M. J. Lee , T. N. Chung , et al., “The Immune Modulatory Effects of Mitochondrial Transplantation on Cecal Slurry Model in Rat,” Critical Care (London, England) 25, no. 1 (2021): 20. 33413559 10.1186/s13054-020-03436-x PMC7789332

[221] F. Zhang , X. Zheng , F. Zhao , et al., “TFAM‐Mediated Mitochondrial Transfer of MSCs Improved the Permeability Barrier in Sepsis‐associated Acute Lung Injury,” Apoptosis: An International Journal on Programmed Cell Death 28, no. 7‐8 (2023): 1048–1059. 37060506 10.1007/s10495-023-01847-z

[222] G. B. Kubat , M. Ozler , O. Ulger , et al., “The Effects of Mesenchymal Stem Cell Mitochondrial Transplantation on Doxorubicin‐mediated Nephrotoxicity in Rats,” Journal of Biochemical and Molecular Toxicology 35, no. 1 (2021): e22612. 32870571 10.1002/jbt.22612

[223] M. J. Kim , J. M. Lee , K. Min , and Y. S. Choi , “Xenogeneic Transplantation of Mitochondria Induces Muscle Regeneration in an in Vivo Rat Model of Dexamethasone‐induced Atrophy,” Journal of Muscle Research and Cell Motility 45, no. 2 (2024): 53–68. 36802005 10.1007/s10974-023-09643-7

[224] S. E. Alway , H. G. Paez , C. R. Pitzer , et al., “Xenogeneic Transplantation of Mitochondria Induces Muscle Regeneration in an in Vivo,” Journal of Cachexia, Sarcopenia and Muscle 14, no. 1 (2023): 493–507. 36604839 10.1002/jcsm.13153 PMC9891964

[225] J. Suh , N. K. Kim , W. Shim , et al., “Mitochondrial Fragmentation and Donut Formation Enhance Mitochondrial Secretion to Promote Osteogenesis,” Cell metabolism 35, no. 2 (2023): 345–360. e7. 36754021 10.1016/j.cmet.2023.01.003

[226] C. Y. Chang , M. Z. Liang , and L. Chen , “Current Progress of Mitochondrial Transplantation That Promotes Neuronal Regeneration,” Translational Neurodegeneration 8 (2019): 17. 31210929 10.1186/s40035-019-0158-8 PMC6567446

[227] L. Chien , M. Z. Liang , C. Y. Chang , et al., “Mitochondrial Therapy Promotes Regeneration of Injured Hippocampal Neurons,” Biochimica et Biophysica Acta ‐ Molecular Basis of Disease 1864, no. 9 Pt B (2018): 3001–3012. 29913215 10.1016/j.bbadis.2018.06.012

[228] G. Nascimento‐Dos‐Santos , E. de‐Souza‐Ferreira , R. Lani , et al., “Neuroprotection From Optic Nerve Injury and Modulation of Oxidative Metabolism by Transplantation of Active Mitochondria to the Retina,” Biochimica et Biophysica Acta ‐ Molecular Basis of Disease 1866, no. 5 (2020): 165686. 31953215 10.1016/j.bbadis.2020.165686

[229] G. M. Smith and G. Gallo , “The Role of Mitochondria in Axon Development and Regeneration,” Developmental Neurobiology 78, no. 3 (2018): 221–237. 29030922 10.1002/dneu.22546 PMC5816701

[230] T. Chen , Y. Zhu , J. Jia , et al., “Mitochondrial Transplantation Promotes Remyelination and Long‐Term Locomotion Recovery Following Cerebral Ischemia,” Mediators of Inflammation 2022 (2022): 1346343. 36157892 10.1155/2022/1346343 PMC9499812

[231] J. L. Gollihue , S. P. Patel , and A. G. Rabchevsky , “Mitochondrial Transplantation Strategies as Potential Therapeutics for central Nervous System Trauma,” Neural Regeneration Research 13, no. 2 (2018): 194–197. 29557359 10.4103/1673-5374.226382 PMC5879881

[232] C. Schiliro and B. L. Firestein , “Mechanisms of Metabolic Reprogramming in Cancer Cells Supporting Enhanced Growth and Proliferation,” Cells 10, no. 5 (2021): 1056. 33946927 10.3390/cells10051056 PMC8146072

[233] J. Ježek , K. F. Cooper , and R. Strich , “Reactive Oxygen Species and Mitochondrial Dynamics: The Yin and Yang of Mitochondrial Dysfunction and Cancer Progression,” Antioxidants 7, no. 1 (2018): 13. 29337889 10.3390/antiox7010013 PMC5789323

[234] J. L. Spees , S. D. Olson , M. J. Whitney , and D. J. Prockop , “Mitochondrial Transfer Between Cells Can Rescue Aerobic Respiration,” PNAS 103, no. 5 (2006): 1283–1288. 16432190 10.1073/pnas.0510511103 PMC1345715

[235] C. Sun , X. Liu , B. Wang , et al., “Endocytosis‐mediated Mitochondrial Transplantation: Transferring Normal human Astrocytic Mitochondria Into Glioma Cells Rescues Aerobic Respiration and Enhances Radiosensitivity,” Theranostics 9, no. 12 (2019): 3595–3607. 31281500 10.7150/thno.33100 PMC6587163

[236] J. C. Chang , H. S. Chang , Y. C. Wu , et al., “Mitochondrial Transplantation Regulates Antitumour Activity, Chemoresistance and Mitochondrial Dynamics in Breast Cancer,” Journal of Experimental & Clinical Cancer Research 38 (2019): 30. 30674338 10.1186/s13046-019-1028-z PMC6343292

[237] Z. Yu , Y. Hou , W. Zhou , et al., “The Effect of Mitochondrial Transplantation Therapy From Different Gender on Inhibiting Cell Proliferation of Malignant Melanoma,” International Journal of Biological Sciences 17, no. 8 (2021): 2021–2033. 34131403 10.7150/ijbs.59581 PMC8193273

[238] R. L. Elliott , X. P. Jiang , and J. F. Head , “Mitochondria Organelle Transplantation: Introduction of Normal Epithelial Mitochondria Into human Cancer Cells Inhibits Proliferation and Increases Drug Sensitivity,” Breast Cancer Research and Treatment 136, no. 2 (2012): 347–354. 23080556 10.1007/s10549-012-2283-2

[239] W. Zhou , Z. Zhao , Z. Yu , et al., “Mitochondrial Transplantation Therapy Inhibits the Proliferation of Malignant Hepatocellular Carcinoma and Its Mechanism,” Mitochondrion 65 (2022): 11–22. 35504558 10.1016/j.mito.2022.04.004

[240] V. Aggarwal , H. S. Tuli , A. Varol , et al., “Role of Reactive Oxygen Species in Cancer Progression: Molecular Mechanisms and Recent Advancements,” Biomolecules 9, no. 11 (2019): 735. 31766246 10.3390/biom9110735 PMC6920770

[241] A. Cruz‐Gregorio , A. K. Aranda‐Rivera , J. Pedraza‐Chaverri , et al., “Redox‐sensitive Signaling Pathways in Renal Cell Carcinoma,” BioFactors (Oxford, England) 48, no. 2 (2022): 342–358. 34590744 10.1002/biof.1784

[242] E. C. Cheung , G. M. DeNicola , C. Nixon , et al., “Dynamic ROS Control by TIGAR Regulates the Initiation and Progression of Pancreatic Cancer,” Cancer Cell 37, no. 2 (2020): 168–182. e4. 31983610 10.1016/j.ccell.2019.12.012 PMC7008247

[243] C. Wiel , K. Le Gal , M. X. Ibrahim , et al., “BACH1 Stabilization by Antioxidants Stimulates Lung Cancer Metastasis,” Cell 178, no. 2 (2019): 330–345. e22. 31257027 10.1016/j.cell.2019.06.005

[244] E. Piskounova , M. Agathocleous , M. M. Murphy , et al., “Oxidative Stress Inhibits Distant Metastasis by human Melanoma Cells,” Nature 527, no. 7577 (2015): 186–191. 26466563 10.1038/nature15726 PMC4644103

[245] L. F. Abbott and S. B. Nelson , “Synaptic Plasticity: Taming the Beast,” Nature Neuroscience 3, no. 11 (2000): 1178–1183. 11127835 10.1038/81453

[246] A. Citri and R. C. Malenka , “Synaptic Plasticity: Multiple Forms, Functions, and Mechanisms,” Neuropsychopharmacol Off Publ Am Coll Neuropsychopharmacol 33, no. 1 (2008): 18–41. 10.1038/sj.npp.1301559 17728696

[247] A. S. Dashkova , V. I. Kovalev , A. V. Chaplygina , et al., “Unique Properties of Synaptosomes and Prospects for Their Use for the Treatment of Alzheimer's Disease,” Biochemistry (Moscow) 89, no. 6 (2024): 1031–1044. 38981699 10.1134/S0006297924060051

[248] A. Faria‐Pereira and V. A. Morais , “Synapses: The Brain's Energy‐Demanding Sites,” International Journal of Molecular Sciences 23, no. 7 (2022): 3627. 35408993 10.3390/ijms23073627 PMC8998888

[249] Z. Li , K. I. Okamoto , Y. Hayashi , and M. Sheng , “The Importance of Dendritic Mitochondria in the Morphogenesis and Plasticity of Spines and Synapses,” Cell 119, no. 6 (2004): 873–887. 15607982 10.1016/j.cell.2004.11.003

[250] M. A. Sutton and E. M. Schuman , “Dendritic Protein Synthesis, Synaptic Plasticity, and Memory,” Cell 127, no. 1 (2006): 49–58. 17018276 10.1016/j.cell.2006.09.014

[251] Z. H. Sheng and Q. Cai , “Mitochondrial Transport in Neurons: Impact on Synaptic Homeostasis and Neurodegeneration,” Nature Reviews Neuroscience 13, no. 2 (2012): 77–93. 22218207 10.1038/nrn3156 PMC4962561

[252] B. Zhang , Y. Gao , Q. Li , et al., “Effects of Brain‐Derived Mitochondria on the Function of Neuron and Vascular Endothelial Cell after Traumatic Brain Injury,” World neurosurgery 138 (2020): e1–e9. 31816451 10.1016/j.wneu.2019.11.172

[253] C. Severini , “Neurotrophic Factors in Health and Disease,” Cells 12, no. 1 (2022): 47. 36611840 10.3390/cells12010047 PMC9818562

[254] E. E. Benarroch , “Brain‐derived Neurotrophic Factor: Regulation, Effects, and Potential Clinical Relevance,” Neurology 84, no. 16 (2015): 1693–1704. 25817841 10.1212/WNL.0000000000001507

[255] F. Zheng , Y. Luo , and H. Wang , “Regulation of BDNF‐mediated Transcription of Immediate Early Gene Arc by Intracellular Calcium and Calmodulin,” Journal of Neuroscience Research 87, no. 2 (2009): 380–392. 18798281 10.1002/jnr.21863 PMC2628963

[256] A. J. Kowaltowski , S. L. Menezes‐Filho , E. A. Assali , et al., “Mitochondrial Morphology Regulates Organellar Ca^2+^ Uptake and Changes Cellular Ca^2+^ Homeostasis,” FASEB J Off Publ Fed Am Soc Exp Biol 33, no. 12 (2019): 13176–13188. 10.1096/fj.201901136R PMC9272750 31480917

[257] L. Luo , “Architectures of Neuronal Circuits,” Science 373, no. 6559 (2021): eabg7285. 34516844 10.1126/science.abg7285 PMC8916593

[258] H. Eo , S. H. Yu , Y. Choi , et al., “Mitochondrial Transplantation Exhibits Neuroprotective Effects and Improves Behavioral Deficits in an Animal Model of Parkinson's Disease,” Neurotherapeutics: The journal of the American Society for Experimental NeuroTherapeutics 21, no. 4 (2024): e00355. 38580511 10.1016/j.neurot.2024.e00355 PMC11067340

[259] F. Bradke , J. W. Fawcett , and M. E. Spira , “Assembly of a New Growth Cone After Axotomy: The Precursor to Axon Regeneration,” Nature Reviews Neuroscience 13, no. 3 (2012): 183–193. 22334213 10.1038/nrn3176

[260] B. Yang , F. Zhang , F. Cheng , et al., “Strategies and Prospects of Effective Neural Circuits Reconstruction After Spinal Cord Injury,” Cell Death & Disease 11, no. 6 (2020): 1–14. 32513969 10.1038/s41419-020-2620-z PMC7280216

[261] A. Oyarzabal and I. Marin‐Valencia , “Synaptic Energy Metabolism and Neuronal Excitability, in Sickness and Health,” Journal of Inherited Metabolic Disease 42, no. 2 (2019): 220–236. 30734319 10.1002/jimd.12071

[262] B. Lu , G. Nagappan , and Y. Lu , “BDNF and Synaptic Plasticity, Cognitive Function, and Dysfunction,” Handbook of Experimental Pharmacology 220 (2014): 223–250. 24668475 10.1007/978-3-642-45106-5_9

[263] S. S. Kang , M. P. Keasey , S. A. Arnold , et al., “Endogenous CNTF Mediates Stroke‐induced Adult CNS Neurogenesis in Mice,” Neurobiology of Disease 49 (2013): 68–78. 22960105 10.1016/j.nbd.2012.08.020 PMC3657597

[264] Y. Xiao and T. Czopka , “Myelination‐independent Functions of Oligodendrocyte Precursor Cells in Health and Disease,” Nature Neuroscience 26, no. 10 (2023): 1663–1669. 37653126 10.1038/s41593-023-01423-3

[265] A. Lopez Juarez , D. He , and Q. Richard Lu , “Oligodendrocyte Progenitor Programming and Reprogramming: Toward Myelin Regeneration,” Brain Research 1638 (2016): 209–220. 26546966 10.1016/j.brainres.2015.10.051 PMC5119932

[266] A. M. Bertholet , T. Delerue , A. M. Millet , et al., “Mitochondrial Fusion/Fission Dynamics in Neurodegeneration and Neuronal Plasticity,” Neurobiology of Disease 90 (2016): 3–19. 26494254 10.1016/j.nbd.2015.10.011

[267] M. A. Clark and J. W. Shay , “Mitochondrial Transformation of Mammalian Cells,” Nature 295, no. 5850 (1982): 605–607. 7057918 10.1038/295605a0

[268] J. D. McCully , D. B. Cowan , C. A. Pacak , et al., “Injection of Isolated Mitochondria During Early Reperfusion for Cardioprotection,” American Journal of Physiology‐Heart and Circulatory Physiology 296, no. 1 (2009): H94–H105. 18978192 10.1152/ajpheart.00567.2008 PMC2637784

[269] K. Tripathi and D. Ben‐Shachar , “Mitochondria in the Central Nervous System in Health and Disease: The Puzzle of the Therapeutic Potential of Mitochondrial Transplantation,” Cells 13, no. 5 (2024): 410. 38474374 10.3390/cells13050410 PMC10930936

[270] J. Suh and Y. S. Lee , “Mitochondria as Secretory Organelles and Therapeutic Cargos,” Experimental & Molecular Medicine 56, no. 1 (2024): 66–85. 38172601 10.1038/s12276-023-01141-7 PMC10834547

[271] N. Borcherding and J. R. Brestoff , “The Power and Potential of Mitochondria Transfer,” Nature 623, no. 7986 (2023): 283–291. 37938702 10.1038/s41586-023-06537-z PMC11590279

[272] Y. Nakamura , J. H. Park , and K. Hayakawa , “Therapeutic Use of Extracellular Mitochondria in CNS Injury and Disease,” Experimental Neurology 324 (2020): 113114. 31734316 10.1016/j.expneurol.2019.113114 PMC6980730

[273] F. Liu , J. Lu , A. Manaenko , J. Tang , and Q. Hu , “Mitochondria in Ischemic Stroke: New Insight and Implications,” Aging and Disease 9, no. 5 (2018): 924–937. 30271667 10.14336/AD.2017.1126 PMC6147588

[274] C. Li , M. K. H. Cheung , S. Han , et al., “Mesenchymal Stem Cells and Their Mitochondrial Transfer: A Double‐edged Sword,” Bioscience Reports 39, no. 5 (2019): BSR20182417. 30979829 10.1042/BSR20182417 PMC6500894

[275] D. Liu , Y. Gao , J. Liu , et al., “Intercellular Mitochondrial Transfer as a Means of Tissue Revitalization,” Signal Transduction and Targeted Therapy 6, no. 1 (2021): 65. 33589598 10.1038/s41392-020-00440-z PMC7884415

[276] S. Paliwal , R. Chaudhuri , A. Agrawal , and S. Mohanty , “Regenerative Abilities of Mesenchymal Stem Cells Through Mitochondrial Transfer,” Journal of Biomedical Science 25 (2018): 31. 29602309 10.1186/s12929-018-0429-1 PMC5877369

[277] J. C. Chang , Y. C. Chao , H. S. Chang , et al., “Intranasal Delivery of Mitochondria for Treatment of Parkinson's Disease Model Rats Lesioned With 6‐hydroxydopamine,” Scientific Reports 11, no. 1 (2021): 10597. 34011937 10.1038/s41598-021-90094-w PMC8136477

[278] K. Nitzan , S. Benhamron , M. Valitsky , et al., “Mitochondrial Transfer Ameliorates Cognitive Deficits, Neuronal Loss, and Gliosis in Alzheimer's Disease Mice,” Journal of Alzheimer's Disease 72, no. 2 (2019): 587–604. 10.3233/JAD-190853 31640104

[279] S. Sweetat , K. Nitzan , N. Suissa , et al., “The Beneficial Effect of Mitochondrial Transfer Therapy in 5XFAD Mice via Liver‐Serum‐Brain Response,” Cells 12, no. 7 (2023): 1006. 37048079 10.3390/cells12071006 PMC10093713

[280] Z. Zhang , D. Wei , Z. Li , et al., “Hippocampal Mitochondrial Transplantation Alleviates Age‐Associated Cognitive Decline via Enhancing Wnt Signaling and Neurogenesis,” Computational Intelligence and Neuroscience 2022 (2022): 9325302. 35685133 10.1155/2022/9325302 PMC9173953

[281] H. Ma , T. Jiang , W. Tang , et al., “Transplantation of Platelet‐derived Mitochondria Alleviates Cognitive Impairment and Mitochondrial Dysfunction in db/db Mice,” Clinical Science (London, England: 1979) 134, no. 16 (2020): 2161–2175. 32794577 10.1042/CS20200530

[282] G. Javani , S. Babri , F. Farajdokht , et al., “Mitochondrial Transplantation Improves Anxiety‐ and Depression‐Like Behaviors in Aged Stress‐exposed Rats,” Mechanisms of Ageing and Development 202 (2022): 111632. 35065970 10.1016/j.mad.2022.111632

[283] Z. Zhao , Z. Yu , Y. Hou , et al., “Improvement of Cognitive and Motor Performance With Mitotherapy in Aged Mice,” International Journal of Biological Sciences 16, no. 5 (2020): 849–858. 32071554 10.7150/ijbs.40886 PMC7019143

[284] J. L. Gollihue , S. P. Patel , K. C. Eldahan , et al., “Effects of Mitochondrial Transplantation on Bioenergetics, Cellular Incorporation, and Functional Recovery After Spinal Cord Injury,” Journal of Neurotrauma 35, no. 15 (2018): 1800–1818. 29648982 10.1089/neu.2017.5605 PMC6053898

[285] Z. Pourmohammadi‐Bejarpasi , A. M. Roushandeh , A. Saberi , et al., “Mesenchymal Stem Cells‐derived Mitochondria Transplantation Mitigates I/R‐induced Injury, Abolishes I/R‐induced Apoptosis, and Restores Motor Function in Acute Ischemia Stroke Rat Model,” Brain Research Bulletin 165 (2020): 70–80. 33010349 10.1016/j.brainresbull.2020.09.018

[286] Y. Nakamura , E. H. Lo , and K. Hayakawa , “Placental Mitochondria Therapy for Cerebral Ischemia‐Reperfusion Injury in Mice,” Stroke; A Journal of Cerebral Circulation 51, no. 10 (2020): 3142–3146. 10.1161/STROKEAHA.120.030152 PMC7530055 32819193

[287] H. Li , C. Wang , T. He , et al., “Mitochondrial Transfer From Bone Marrow Mesenchymal Stem Cells to Motor Neurons in Spinal Cord Injury Rats via Gap Junction,” Theranostics 9, no. 7 (2019): 2017–2035. 31037154 10.7150/thno.29400 PMC6485285

[288] V. A. Babenko , D. N. Silachev , L. D. Zorova , et al., “Improving the Post‐Stroke Therapeutic Potency of Mesenchymal Multipotent Stromal Cells by Cocultivation with Cortical Neurons: The Role of Crosstalk between Cells,” Stem Cells Translational Medicine 4, no. 9 (2015): 1011–1020. 26160961 10.5966/sctm.2015-0010 PMC4542870

[289] H. K. Yip , N. K. Dubey , K. C. Lin , et al., “Melatonin Rescues Cerebral Ischemic Events Through Upregulated Tunneling Nanotube‐mediated Mitochondrial Transfer and Downregulated Mitochondrial Oxidative Stress in Rat Brain,” Biomedicine & Pharmacotherapy 139 (2021): 111593. 33865018 10.1016/j.biopha.2021.111593

[290] N. V. Bobkova , D. Y. Zhdanova , N. V. Belosludtseva , et al., “Intranasal Administration of Mitochondria Improves Spatial Memory in Olfactory Bulbectomized Mice,” Experimental Biology and Medicine 247, no. 5 (2022): 416–425. 34727745 10.1177/15353702211056866 PMC8919317

[291] J. F. Alexander , A. V. Seua , L. D. Arroyo , et al., “Nasal Administration of Mitochondria Reverses Chemotherapy‐induced Cognitive Deficits,” Theranostics 11, no. 7 (2021): 3109–3130. 33537077 10.7150/thno.53474 PMC7847685

[292] N. Boukelmoune , G. S. Chiu , A. Kavelaars , and C. J. Heijnen , “Mitochondrial Transfer From Mesenchymal Stem Cells to Neural Stem Cells Protects Against the Neurotoxic Effects of Cisplatin,” Acta Neuropathologica Communications 6, no. 1 (2018): 139. 30541620 10.1186/s40478-018-0644-8 PMC6292021

[293] L. Peruzzotti‐Jametti , J. D. Bernstock , C. M. Willis , et al., “Neural Stem Cells Traffic Functional Mitochondria via Extracellular Vesicles,” Plos Biology 19, no. 4 (2021): e3001166. 33826607 10.1371/journal.pbio.3001166 PMC8055036

[294] K. M. Dave , D. B. Stolz , V. R. Venna , et al., “Mitochondria‐containing Extracellular Vesicles (EV) Reduce Mouse Brain Infarct Sizes and EV/HSP27 Protect Ischemic Brain Endothelial Cultures,” J Control Release Off J Control Release Soc 354 (2023): 368–393. 10.1016/j.jconrel.2023.01.025 PMC9974867 36642252

[295] D. B. Cowan , R. Yao , V. Akurathi , et al., “Intracoronary Delivery of Mitochondria to the Ischemic Heart for Cardioprotection,” PLoS ONE 11, no. 8 (2016): e0160889. 27500955 10.1371/journal.pone.0160889 PMC4976938

[296] A. K. Kaza , I. Wamala , I. Friehs , et al., “Myocardial Rescue With Autologous Mitochondrial Transplantation in a Porcine Model of Ischemia/Reperfusion,” Journal of Thoracic and Cardiovascular Surgery 153, no. 4 (2017): 934–943. 27938904 10.1016/j.jtcvs.2016.10.077

[297] B. Shin , M. Y. Saeed , J. J. Esch , et al., “A Novel Biological Strategy for Myocardial Protection by Intracoronary Delivery of Mitochondria: Safety and Efficacy,” JACC Basic Transl Sci 4, no. 8 (2019): 871–888. 31909298 10.1016/j.jacbts.2019.08.007 PMC6938990

[298] G. Alvise , P. D. Ilias , D. Thomas , et al., “Mitochondrial Transplantation for Myocardial Protection in Ex‐situ‒Perfused Hearts Donated After Circulatory Death,” J Heart Lung Transplant Off Publ Int Soc Heart Transplant 39, no. 11 (2020): 1279–1288. 10.1016/j.healun.2020.06.023 32703639

[299] D. Blitzer , A. Guariento , I. P. Doulamis , et al., “Delayed Transplantation of Autologous Mitochondria for Cardioprotection in a Porcine Model,” Annals of Thoracic Surgery 109, no. 3 (2020): 711–719. 31421103 10.1016/j.athoracsur.2019.06.075

[300] K. Moskowitzova , B. Shin , K. Liu , et al., “Mitochondrial Transplantation Prolongs Cold Ischemia Time in Murine Heart Transplantation,” J Heart Lung Transplant Off Publ Int Soc Heart Transplant 38, no. 1 (2019): 92–99. 10.1016/j.healun.2018.09.025 PMC6574228 30391192

[301] I. P. Doulamis , A. Guariento , T. Duignan , et al., “Mitochondrial Transplantation for Myocardial Protection in Diabetic Hearts,” Eur J Cardio‐Thorac Surg Off J Eur Assoc Cardio‐Thorac Surg 57, no. 5 (2020): 836–845. 10.1093/ejcts/ezz326 31782771

[302] Y. Zhang , Z. Yu , D. Jiang , et al., “iPSC‐MSCs With High Intrinsic MIRO1 and Sensitivity to TNF‐α Yield Efficacious Mitochondrial Transfer to Rescue Anthracycline‐Induced Cardiomyopathy,” Stem Cell Reports 7, no. 4 (2016): 749–763. 27641650 10.1016/j.stemcr.2016.08.009 PMC5063626

[303] G. Ikeda , M. R. Santoso , Y. Tada , et al., “Mitochondria‐Rich Extracellular Vesicles From Autologous Stem Cell‐Derived Cardiomyocytes Restore Energetics of Ischemic Myocardium,” Journal of the American College of Cardiology 77, no. 8 (2021): 1073–1088. 33632482 10.1016/j.jacc.2020.12.060 PMC8626617

[304] X. Shi , H. Bai , M. Zhao , et al., “Treatment of Acetaminophen‐induced Liver Injury With Exogenous Mitochondria in Mice,” Transl Res J Lab Clin Med 196 (2018): 31–41. 10.1016/j.trsl.2018.02.003 29548626

[305] O. Ulger , G. B. Kubat , Z. Cicek , et al., “The Effects of Mitochondrial Transplantation in Acetaminophen‐induced Liver Toxicity in Rats,” Life Sciences 279 (2021): 119669. 34081988 10.1016/j.lfs.2021.119669

[306] A. Fu , X. Shi , H. Zhang , and B. Fu , “Mitotherapy for Fatty Liver by Intravenous Administration of Exogenous Mitochondria in Male Mice,” Frontiers in pharmacology 8 (2017): 241. 28536524 10.3389/fphar.2017.00241 PMC5422541

[307] H. C. Lin , S. Y. Liu , H. S. Lai , and I. R. Lai , “Isolated Mitochondria Infusion Mitigates Ischemia‐reperfusion Injury of the Liver in Rats,” Shock Augusta Ga 39, no. 3 (2013): 304–310. 23364428 10.1097/SHK.0b013e318283035f

[308] T. Lu , J. Zhang , J. Cai , et al., “Extracellular Vesicles Derived From Mesenchymal Stromal Cells as Nanotherapeutics for Liver Ischaemia‐reperfusion Injury by Transferring Mitochondria to Modulate the Formation of Neutrophil Extracellular Traps,” Biomaterials 284 (2022): 121486. 35447404 10.1016/j.biomaterials.2022.121486

[309] C. M. Cloer , C. S. Givens , L. K. Buie , et al., “Mitochondrial Transplant After Ischemia Reperfusion Promotes Cellular Salvage and Improves Lung Function During Ex‐vivo Lung Perfusion,” J Heart Lung Transplant Off Publ Int Soc Heart Transplant 42, no. 5 (2023): 575–584. 10.1016/j.healun.2023.01.002 36707296

[310] K. Moskowitzova , A. Orfany , K. Liu , et al., “Mitochondrial Transplantation Enhances Murine Lung Viability and Recovery After Ischemia‐reperfusion Injury,” Am J Physiol—Lung Cell Mol Physiol 318, no. 1 (2020): L78–L88. 31693391 10.1152/ajplung.00221.2019 PMC6985877

[311] T. Huang , T. Zhang , X. Jiang , et al., “Iron Oxide Nanoparticles Augment the Intercellular Mitochondrial Transfer–mediated Therapy,” Science Advances 7, no. 40 (2021): eabj0534. 34586849 10.1126/sciadv.abj0534 PMC8480934

[312] A. Rossi , A. Asthana , C. Riganti , et al., “Mitochondria Transplantation Mitigates Damage in an in Vitro Model of Renal Tubular Injury and in an Ex Vivo Model of DCD Renal Transplantation,” Annals of Surgery 278, no. 6 (2023): e1313–e1326. 37450698 10.1097/SLA.0000000000006005 PMC10631499

[313] I. P. Doulamis , A. Guariento , T. Duignan , et al., “Mitochondrial Transplantation by Intra‐arterial Injection for Acute Kidney Injury,” American Journal of Physiology. Renal Physiology 319, no. 3 (2020): F403–F413. 32686525 10.1152/ajprenal.00255.2020 PMC7509287

[314] M. Zhao , S. Liu , C. Wang , et al., “Mesenchymal Stem Cell‐Derived Extracellular Vesicles Attenuate Mitochondrial Damage and Inflammation by Stabilizing Mitochondrial DNA,” ACS Nano 15, no. 1 (2021): 1519–1538. 33369392 10.1021/acsnano.0c08947

[315] H. Cao , Y. Cheng , H. Gao , et al., “In Vivo Tracking of Mesenchymal Stem Cell‐Derived Extracellular Vesicles Improving Mitochondrial Function in Renal Ischemia‐Reperfusion Injury,” ACS Nano 14, no. 4 (2020): 4014–4026. 32212674 10.1021/acsnano.9b08207

[316] J. M. Lee , J. W. Hwang , M. J. Kim , et al., “Mitochondrial Transplantation Modulates Inflammation and Apoptosis, Alleviating Tendinopathy both in Vivo and in Vitro,” Antioxid Basel Switz 10, no. 5 (2021): 696. 10.3390/antiox10050696 PMC8146308 33925007

[317] A. Orfany , C. G. Arriola , I. P. Doulamis , et al., “Mitochondrial Transplantation Ameliorates Acute Limb Ischemia,” Journal of Vascular Surgery 71, no. 3 (2020): 1014–1026. 31353269 10.1016/j.jvs.2019.03.079

[318] J. Sun , H. T. J. Lo , L. Fan , et al., “High‐efficiency Quantitative Control of Mitochondrial Transfer Based on Droplet Microfluidics and Its Application on Muscle Regeneration,” Science Advances 8, no. 33 (2022): eabp9245. 35977014 10.1126/sciadv.abp9245 PMC9385153

[319] A. R. Lee , J. S. Woo , S. Y. Lee , et al., “Mitochondrial Transplantation Ameliorates the Development and Progression of Osteoarthritis,” Immune Netw 22, no. 2 (2022): e14. 35573148 10.4110/in.2022.22.e14 PMC9066007

[320] Z. Zhang , C. Yan , J. Miao , et al., “Muscle‐Derived Mitochondrial Transplantation Reduces Inflammation, Enhances Bacterial Clearance, and Improves Survival in Sepsis,” Shock Augusta Ga 56, no. 1 (2021): 108–118. 33060455 10.1097/SHK.0000000000001681

[321] L. R. P. de Carvalho , S. C. Abreu , L. L. de Castro , et al., “Mitochondria‐Rich Fraction Isolated from Mesenchymal Stromal Cells Reduces Lung and Distal Organ Injury in Experimental Sepsis,” Critical Care Medicine 49, no. 9 (2021): e880–e890. 33870913 10.1097/CCM.0000000000005056

[322] Y. S. Kim , H. A. R. Lee , M. J. Lee , et al., “The Effects of Mitochondrial Transplantation on Sepsis Depend on the Type of Cell From Which They Are Isolated,” International Journal of Molecular Sciences 24, no. 12 (2023): 10113. 37373260 10.3390/ijms241210113 PMC10299019

[323] A. C. Court , A. Le‐Gatt , P. Luz‐Crawford , et al., “Mitochondrial Transfer From MSCs to T Cells Induces Treg Differentiation and Restricts Inflammatory Response,” Embo Reports 21, no. 2 (2020): e48052. 31984629 10.15252/embr.201948052 PMC7001501

[324] J. C. Chang , H. S. Chang , Y. C. Wu , et al., “Antitumor Actions of Intratumoral Delivery of Membrane‐Fused Mitochondria in a Mouse Model of Triple‐Negative Breast Cancers,” OncoTargets Ther 13 (2020): 5241–5255. 10.2147/OTT.S238143 PMC7294573 32606744

[325] J. C. Chang , H. S. Chang , C. Y. Yeh , et al., “Regulation of Mitochondrial Fusion and Mitophagy by Intra‐tumoral Delivery of Membrane‐fused Mitochondria or Midiv‐1 Enhances Sensitivity to Doxorubicin in Triple‐negative Breast Cancer,” Biomedicine & Pharmacotherapy 153 (2022): 113484. 36076583 10.1016/j.biopha.2022.113484

[326] A. Fu , Y. Hou , Z. Yu , et al., “Healthy Mitochondria Inhibit the Metastatic Melanoma in Lungs,” Int J Biol Sci 15, no. 12 (2019): 2707–2718. 31754341 10.7150/ijbs.38104 PMC6854369

[327] C. A. Pinkert , M. H. Irwin , L. W. Johnson , and R. J. Moffatt , “Mitochondria Transfer Into Mouse Ova by Microinjection,” Transgenic Research 6, no. 6 (1997): 379–383. 9423287 10.1023/a:1018431316831

[328] A. Caicedo , V. Fritz , J. M. Brondello , et al., “MitoCeption as a New Tool to Assess the Effects of Mesenchymal Stem/Stromal Cell Mitochondria on Cancer Cell Metabolism and Function,” Scientific Reports 5 (2015): 9073. 25766410 10.1038/srep09073 PMC4358056

[329] F. Cabrera , M. Ortega , F. Velarde , et al., “Primary Allogeneic Mitochondrial Mix (PAMM) Transfer/Transplant by MitoCeption to Address Damage in PBMCs Caused by Ultraviolet Radiation,” BMC Biotechnology [Electronic Resource] 19, no. 1 (2019): 42. 31253149 10.1186/s12896-019-0534-6 PMC6599354

[330] T. Macheiner , V. H. I. Fengler , M. Agreiter , et al., “Magnetomitotransfer: An Efficient Way for Direct Mitochondria Transfer Into Cultured human Cells,” Scientific Reports 6 (2016): 35571. 27767193 10.1038/srep35571 PMC5073296

[331] J. C. Chang , K. H. Liu , Y. C. Li , et al., “Functional Recovery of human Cells Harbouring the Mitochondrial DNA Mutation MERRF A8344G via Peptide‐mediated Mitochondrial Delivery,” Neuro‐Signals 21, no. 3‐4 (2013): 160–173. 23006856 10.1159/000341981

[332] S. Wu , A. Zhang , S. Li , et al., “Polymer Functionalization of Isolated Mitochondria for Cellular Transplantation and Metabolic Phenotype Alteration,” Adv Sci Weinh Baden‐Wurtt Ger 5, no. 3 (2018): 1700530. 10.1002/advs.201700530 PMC5867055 29593955

[333] T. H. Wu , E. Sagullo , D. Case , et al., “Mitochondrial Transfer by Photothermal Nanoblade Restores Metabolite Profile in Mammalian Cells,” Cell metabolism 23, no. 5 (2016): 921–929. 27166949 10.1016/j.cmet.2016.04.007 PMC5062745

[334] A. J. Sercel , A. N. Patananan , T. Man , et al., “Stable Transplantation of human Mitochondrial DNA by High‐throughput, Pressurized Isolated Mitochondrial Delivery,” Elife 10 (2021): e63102. 33438576 10.7554/eLife.63102 PMC7864630

[335] T. H. Wu , Y. C. Wu , E. Sagullo , et al., “Direct Nuclear Delivery of DNA by Photothermal Nanoblade,” J Lab Autom 20, no. 6 (2015): 659–662. 25900925 10.1177/2211068215583630

[336] C. G. Gäbelein , Q. Feng , E. Sarajlic , et al., “Mitochondria Transplantation Between Living Cells,” Plos Biology 20, no. 3 (2022): e3001576. 35320264 10.1371/journal.pbio.3001576 PMC8942278

[337] P. Picone , G. Porcelli , C. C. Bavisotto , et al., “Synaptosomes: New Vesicles for Neuronal Mitochondrial Transplantation,” J Nanobiotechnology 19 (2021): 6. 33407593 10.1186/s12951-020-00748-6 PMC7789323

[338] N. Tseng , S. C. Lambie , C. Q. Huynh , et al., “Mitochondrial Transfer From Mesenchymal Stem Cells Improves Neuronal Metabolism After Oxidant Injury in Vitro: The Role of Miro1,” J Cereb Blood Flow Metab Off J Int Soc Cereb Blood Flow Metab 41, no. 4 (2021): 761–770. 10.1177/0271678X20928147 PMC7983509 32501156

[339] X. Li , Y. Li , Z. Zhang , et al., “Mild Hypothermia Facilitates Mitochondrial Transfer From Astrocytes to Injured Neurons During Oxygen‐glucose Deprivation/Reoxygenation,” Neuroscience Letters 756 (2021): 135940. 33971244 10.1016/j.neulet.2021.135940

[340] X. Y. Cheng , S. Biswas , J. Li , et al., “Human iPSCs Derived Astrocytes Rescue Rotenone‐induced Mitochondrial Dysfunction and Dopaminergic Neurodegeneration in Vitro by Donating Functional Mitochondria,” Transl Neurodegener 9, no. 1 (2020): 13. 32345341 10.1186/s40035-020-00190-6 PMC7325238

[341] K. English , A. Shepherd , N. E. Uzor , et al., “Astrocytes Rescue Neuronal Health After Cisplatin Treatment Through Mitochondrial Transfer,” Acta Neuropathol Commun 8, no. 1 (2020): 36. 32197663 10.1186/s40478-020-00897-7 PMC7082981

[342] M. Mahrouf‐Yorgov , L. Augeul , C. C. Da Silva , et al., “Mesenchymal Stem Cells Sense Mitochondria Released From Damaged Cells as Danger Signals to Activate Their Rescue Properties,” Cell Death and Differentiation 24, no. 7 (2017): 1224–1238. 28524859 10.1038/cdd.2017.51 PMC5520168

[343] H. Han , J. Hu , Q. Yan , et al., “Bone Marrow‐derived Mesenchymal Stem Cells Rescue Injured H9c2 Cells via Transferring Intact Mitochondria Through Tunneling Nanotubes in an in Vitro Simulated Ischemia/Reperfusion Model,” Mol Med Rep 13, no. 2 (2016): 1517–1524. 26718099 10.3892/mmr.2015.4726 PMC4732861

[344] S. Kim , Y. Kim , S. H. Yu , et al., “Platelet‐derived Mitochondria Transfer Facilitates Wound‐closure by Modulating ROS Levels in Dermal Fibroblasts,” Platelets 34, no. 1 (2022): 2151996. 36529914 10.1080/09537104.2022.2151996

[345] P. Jin , Q. Pan , Y. Lin , et al., “Platelets Facilitate Wound Healing by Mitochondrial Transfer and Reducing Oxidative Stress in Endothelial Cells,” Oxid Med Cell Longev 2023 (2023): 2345279. 36860732 10.1155/2023/2345279 PMC9970712

[346] K. Unuma , T. Aki , T. Funakoshi , et al., “Extrusion of Mitochondrial Contents From Lipopolysaccharide‐stimulated Cells: Involvement of Autophagy,” Autophagy 11, no. 9 (2015): 1520–1536. 26102061 10.1080/15548627.2015.1063765 PMC4590602

[347] A. Arjmand , S. Shiranirad , F. Ameritorzani , et al., “Mitochondrial Transplantation Against Gentamicin‐induced Toxicity on Rat Renal Proximal Tubular Cells: The Higher Activity of Female Rat Mitochondria,” In Vitro Cellular & Developmental Biology Animal 59, no. 1 (2023): 31–40. 36630058 10.1007/s11626-022-00743-1

[348] E. Seydi , M. Rahemi , H. Esmaily , et al., “Mitochondrial Transplantation Attenuates Toxicity in Rat Renal Proximal Tubular Cells Caused by Favipiravir,” Journal of Pharmacy and Pharmacology 75, no. 11 (2023): 1458–1466. 37738481 10.1093/jpp/rgad079

[349] A. Arjmand , M. Faizi , M. Rezaei , and J. Pourahmad , “The Effect of Donor Rat Gender in Mitochondrial Transplantation Therapy of Cisplatin‐Induced Toxicity on Rat Renal Proximal Tubular Cells,” Iran J Pharm Res IJPR 22, no. 1 (2023): e135666. 38148888 10.5812/ijpr-135666 PMC10750785

[350] M. J. Kim , J. W. Hwang , C. K. Yun , et al., “Delivery of Exogenous Mitochondria via Centrifugation Enhances Cellular Metabolic Function,” Scientific Reports 8, no. 1 (2018): 3330. 29463809 10.1038/s41598-018-21539-y PMC5820364

[351] V. Budnik , C. Ruiz‐Cañada , and F. Wendler , “Extracellular Vesicles Round off Communication in the Nervous System,” Nature Reviews Neuroscience 17, no. 3 (2016): 160–172. 26891626 10.1038/nrn.2015.29 PMC4989863

[352] S. Ahmad , R. K. Srivastava , P. Singh , et al., “Role of Extracellular Vesicles in Glia‐Neuron Intercellular Communication,” Frontiers in Molecular Neuroscience 15 (2022): 844194. 35493327 10.3389/fnmol.2022.844194 PMC9043804

[353] S. M. Emani , B. L. Piekarski , D. Harrild , et al., “Autologous Mitochondrial Transplantation for Dysfunction After Ischemia‐reperfusion Injury,” Journal of Thoracic and Cardiovascular Surgery 154, no. 1 (2017): 286–289. 28283239 10.1016/j.jtcvs.2017.02.018

[354] A. Yoshimi , K. Ishikawa , C. Niemeyer , and S. C. Grünert , “Pearson Syndrome: A Multisystem Mitochondrial Disease With Bone Marrow Failure,” Orphanet journal of rare diseases 17, no. 1 (2022): 379. 36253820 10.1186/s13023-022-02538-9 PMC9575259

[355] A. Guariento , B. L. Piekarski , I. P. Doulamis , et al., “Autologous Mitochondrial Transplantation for Cardiogenic Shock in Pediatric Patients Following Ischemia‐reperfusion Injury,” Journal of Thoracic and Cardiovascular Surgery 162, no. 3 (2021): 992–1001. 33349443 10.1016/j.jtcvs.2020.10.151

[356] M. Walker , E. Federico , Y. Sancak , and M. R. Levitt , “Mitochondrial Transplantation in Ischemic Stroke: Insights From a First‐in‐Human Brain Trial,” Current Transplantation Reports 11, no. 2 (2024): 53–62.

[357] M. Sun , W. Jiang , N. Mu , et al., “Mitochondrial Transplantation as a Novel Therapeutic Strategy for Cardiovascular Diseases,” Journal of translational medicine 21 (2023): 347. 37231493 10.1186/s12967-023-04203-6 PMC10210445

[358] R. Jain , N. Begum , K. P. Tryphena , et al., “Inter and Intracellular Mitochondrial Transfer: Future of Mitochondrial Transplant Therapy in Parkinson's Disease,” Biomedicine & Pharmacotherapy 159 (2023): 114268. 36682243 10.1016/j.biopha.2023.114268

[359] C. E. D. la Fuente‐Muñoz and C. Arias , “The Therapeutic Potential of Mitochondrial Transplantation for the Treatment of Neurodegenerative Disorders,” Reviews in the Neurosciences 32, no. 2 (2021): 203–217. 33550783 10.1515/revneuro-2020-0068

[360] Y. Chen , F. Yang , Y. Chu , et al., “Mitochondrial Transplantation: Opportunities and Challenges in the Treatment of Obesity, Diabetes, and Nonalcoholic Fatty Liver Disease,” Journal of translational medicine 20, no. 1 (2022): 1–16. 36273156 10.1186/s12967-022-03693-0 PMC9588235

[361] A. Cruz‐Gregorio , A. K. Aranda‐Rivera , I. Amador‐Martinez , and P. Maycotte , “Mitochondrial Transplantation Strategies in Multifaceted Induction of Cancer Cell Death,” Life Sciences 332 (2023): 122098. 37734433 10.1016/j.lfs.2023.122098

[362] E. M. Fock and R. G. Parnova , “Protective Effect of Mitochondria‐Targeted Antioxidants Against Inflammatory Response to Lipopolysaccharide Challenge: A Review,” Pharmaceutics 13, no. 2 (2021): 144. 33499252 10.3390/pharmaceutics13020144 PMC7910823

[363] A. C. Bulua , A. Simon , R. Maddipati , et al., “Mitochondrial Reactive Oxygen Species Promote Production of Proinflammatory Cytokines and Are Elevated in TNFR1‐associated Periodic Syndrome (TRAPS),” Journal of Experimental Medicine 208, no. 3 (2011): 519–533. 21282379 10.1084/jem.20102049 PMC3058571

[364] A. Rimessi , M. Previati , F. Nigro , et al., “Mitochondrial Reactive Oxygen Species and Inflammation: Molecular Mechanisms, Diseases and Promising Therapies,” International Journal of Biochemistry & Cell Biology 81 (2016): 281–293. 27373679 10.1016/j.biocel.2016.06.015

[365] J. R. Mercer , E. Yu , N. Figg , et al., “The Mitochondria‐targeted Antioxidant MitoQ Decreases Features of the Metabolic Syndrome in ATM+/‐/ApoE‐/‐ mice,” Free Radical Biology and Medicine 52, no. 5 (2012): 841–849. 22210379 10.1016/j.freeradbiomed.2011.11.026

[366] R. Ni , T. Cao , S. Xiong , et al., “Therapeutic Inhibition of Mitochondrial Reactive Oxygen Species With mito‐TEMPO Reduces Diabetic Cardiomyopathy,” Free Radical Biology and Medicine 90 (2016): 12–23. 26577173 10.1016/j.freeradbiomed.2015.11.013 PMC5066872

[367] J. McLachlan , E. Beattie , M. P. Murphy , et al., “Combined Therapeutic Benefit of Mitochondria‐targeted Antioxidant, MitoQ10, and Angiotensin Receptor Blocker, Losartan, on Cardiovascular Function,” Journal of Hypertension 32, no. 3 (2014): 555–564. 24309493 10.1097/HJH.0000000000000054 PMC3914904

[368] D. Graham , N. N. Huynh , C. A. Hamilton , et al., “Mitochondria‐targeted Antioxidant MitoQ10 Improves Endothelial Function and Attenuates Cardiac Hypertrophy,” Hypertens Dallas Tex 1979 54, no. 2 (2009): 322–328. 10.1161/HYPERTENSIONAHA.109.130351 19581509

[369] T. A. Ajith , “Role of Mitochondria and Mitochondria‐targeted Agents in Non‐alcoholic Fatty Liver Disease,” Clinical and Experimental Pharmacology & Physiology 45, no. 5 (2018): 413–421. 29112771 10.1111/1440-1681.12886

[370] K. Gariani , K. J. Menzies , D. Ryu , et al., “Eliciting the Mitochondrial Unfolded Protein Response by Nicotinamide Adenine Dinucleotide Repletion Reverses Fatty Liver Disease in Mice,” Hepatol Baltim Md 63, no. 4 (2016): 1190–1204. 10.1002/hep.28245 PMC4805450 26404765

[371] S. A. Mortensen , F. Rosenfeldt , A. Kumar , et al., “The Effect of Coenzyme Q10 on Morbidity and Mortality in Chronic Heart Failure: Results From Q‐SYMBIO: A Randomized Double‐blind Trial,” JACC Heart Fail 2, no. 6 (2014): 641–649. 25282031 10.1016/j.jchf.2014.06.008

[372] F. Forini , P. Canale , G. Nicolini , and G. Iervasi , “Mitochondria‐Targeted Drug Delivery in Cardiovascular Disease: A Long Road to Nano‐Cardio Medicine,” Pharmaceutics 12, no. 11 (2020): 1122. 33233847 10.3390/pharmaceutics12111122 PMC7699942

[373] X. Du , Q. Zeng , Y. Luo , et al., “Application Research of Novel Peptide Mitochondrial‐targeted Antioxidant SS‐31 in Mitigating Mitochondrial Dysfunction,” Mitochondrion 75 (2024): 101846. 38237649 10.1016/j.mito.2024.101846

[374] E. Miquel , A. Cassina , L. Martínez‐Palma , et al., “Neuroprotective Effects of the Mitochondria‐targeted Antioxidant MitoQ in a Model of Inherited Amyotrophic Lateral Sclerosis,” Free Radical Biology and Medicine 70 (2014): 204–213. 24582549 10.1016/j.freeradbiomed.2014.02.019

[375] H. Komaki , N. Faraji , A. Komaki , et al., “Investigation of Protective Effects of Coenzyme Q10 on Impaired Synaptic Plasticity in a Male Rat Model of Alzheimer's Disease,” Brain Research Bulletin 147 (2019): 14–21. 30721766 10.1016/j.brainresbull.2019.01.025

[376] P. J. Flannery and E. Trushina , “Mitochondrial Dysfunction in Alzheimer's Disease and Progress in Mitochondria‐Targeted Therapeutics,” Current Behavioral Neuroscience Reports 6, no. 3 (2019): 88–102.

[377] S. Bido , F. N. Soria , R. Z. Fan , E. Bezard , and K. Tieu , “Mitochondrial Division Inhibitor‐1 Is Neuroprotective in the A53T‐α‐synuclein Rat Model of Parkinson's disease,” Scientific Reports 7, no. 1 (2017): 7495. 28790323 10.1038/s41598-017-07181-0 PMC5548731

[378] S. H. Baek , S. J. Park , J. I. Jeong , et al., “Inhibition of Drp1 Ameliorates Synaptic Depression, Aβ Deposition, and Cognitive Impairment in an Alzheimer's Disease Model,” The Journal of Neuroscience 37, no. 20 (2017): 5099–5110. 28432138 10.1523/JNEUROSCI.2385-16.2017 PMC6596467

[379] Y. Zhao , X. Sun , D. Hu , et al., “ATAD3A oligomerization Causes Neurodegeneration by Coupling Mitochondrial Fragmentation and Bioenergetics Defects,” Nature Communications 10, no. 1 (2019): 1371. 10.1038/s41467-019-09291-x PMC6435701 30914652

[380] L. Zhang , S. Zhang , I. Maezawa , et al., “Modulation of Mitochondrial Complex I Activity Averts Cognitive Decline in Multiple Animal Models of Familial Alzheimer's Disease,” EBioMedicine 2, no. 4 (2015): 294–305. 26086035 10.1016/j.ebiom.2015.03.009 PMC4465115

[381] W. Zhao , M. Varghese , P. Vempati , et al., “Caprylic Triglyceride as a Novel Therapeutic Approach to Effectively Improve the Performance and Attenuate the Symptoms due to the Motor Neuron Loss in ALS Disease,” PLoS ONE 7, no. 11 (2012): e49191. 23145119 10.1371/journal.pone.0049191 PMC3492315

[382] T. W. Tefera , Y. Wong , M. E. Barkl‐Luke , et al., “Triheptanoin Protects Motor Neurons and Delays the Onset of Motor Symptoms in a Mouse Model of Amyotrophic Lateral Sclerosis,” PLoS ONE 11, no. 8 (2016): e0161816. 27564703 10.1371/journal.pone.0161816 PMC5001695

[383] D. A. Butterfield and B. Halliwell , “Oxidative Stress, Dysfunctional Glucose Metabolism and Alzheimer Disease,” Nature Reviews Neuroscience 20, no. 3 (2019): 148–160. 30737462 10.1038/s41583-019-0132-6 PMC9382875

[384] K. Tieu , C. Perier , C. Caspersen , et al., “D‐beta‐hydroxybutyrate Rescues Mitochondrial Respiration and Mitigates Features of Parkinson disease,” Journal of Clinical Investigation 112, no. 6 (2003): 892–901. 12975474 10.1172/JCI18797 PMC193668

[385] Z. Ou , X. Kong , X. Sun , et al., “Metformin Treatment Prevents Amyloid Plaque Deposition and Memory Impairment in APP/PS1 Mice,” Brain, Behavior, and Immunity 69 (2018): 351–363. 29253574 10.1016/j.bbi.2017.12.009

[386] I. Arnoux , M. Willam , N. Griesche , et al., “Metformin Reverses Early Cortical Network Dysfunction and Behavior Changes in Huntington's disease,” Elife 7 (2018): e38744. 30179155 10.7554/eLife.38744 PMC6156080

[387] H. H. Szeto and A. V. Birk , “Serendipity and the Discovery of Novel Compounds That Restore Mitochondrial Plasticity,” Clinical Pharmacology & Therapeutics 96, no. 6 (2014): 672–683. 25188726 10.1038/clpt.2014.174 PMC4267688

[388] V. J. A. Jameson , H. M. Cochemé , A. Logan , et al., “Synthesis of Triphenylphosphonium Vitamin E Derivatives as Mitochondria‐targeted Antioxidants,” Tetrahedron 71, no. 44 (2015): 8444–8453. 26549895 10.1016/j.tet.2015.09.014 PMC4596152

[389] T. Khan , R. Waseem , Z. Zehra , et al., “Mitochondrial Dysfunction: Pathophysiology and Mitochondria‐Targeted Drug Delivery Approaches,” Pharmaceutics 14, no. 12 (2022): 2657. 36559149 10.3390/pharmaceutics14122657 PMC9785072

[390] M. Picard , T. Taivassalo , D. Ritchie , et al., “Mitochondrial Structure and Function Are Disrupted by Standard Isolation Methods,” PLoS ONE 6, no. 3 (2011): e18317. 21512578 10.1371/journal.pone.0018317 PMC3065478

[391] I. R. Lanza and K. S. Nair , “Functional Assessment of Isolated Mitochondria in Vitro,” Methods in Enzymology 457 (2009): 349–372. 19426878 10.1016/S0076-6879(09)05020-4 PMC2782617

[392] A. P. West and G. S. Shadel , “Mitochondrial DNA in Innate Immune Responses and Inflammatory Pathology,” Nature Reviews Immunology 17, no. 6 (2017): 363–375. 10.1038/nri.2017.21 PMC7289178 28393922

[393] A. P. West , W. Khoury‐Hanold , M. Staron , et al., “Mitochondrial DNA Stress Primes the Antiviral Innate Immune Response,” Nature 520, no. 7548 (2015): 553–557. 25642965 10.1038/nature14156 PMC4409480

[394] E. E. Kesner , A. Saada‐Reich , and H. Lorberboum‐Galski , “Characteristics of Mitochondrial Transformation Into Human Cells,” Scientific Reports 6 (2016): 26057. 27184109 10.1038/srep26057 PMC4868981

[395] E. Fernández‐Vizarra , G. Ferrín , A. Pérez‐Martos , et al., “Isolation of Mitochondria for Biogenetical Studies: An Update,” Mitochondrion 10, no. 3 (2010): 253–262. 20034597 10.1016/j.mito.2009.12.148

[396] P. C. Liao , C. Bergamini , R. Fato , et al., “Isolation of Mitochondria From Cells and Tissues,” Methods in Cell Biology 155 (2020): 3–31. 32183964 10.1016/bs.mcb.2019.10.002 PMC8530414

[397] J. A. MacDonald , A. M. Bothun , S. N. Annis , et al., “A Nanoscale, Multi‐parametric Flow Cytometry‐based Platform to Study Mitochondrial Heterogeneity and Mitochondrial DNA Dynamics,” Communications Biology 2 (2019): 258. 31312727 10.1038/s42003-019-0513-4 PMC6624292

[398] D. Greiff and M. Myers , “Effect of Dimethyl Sulphoxide on the Cryo‐tolerance of Mitochondria,” Nature 190 (1961): 1202–1204. 13708435 10.1038/1901202b0

[399] V. N. Nukala , I. N. Singh , L. M. Davis , and P. G. Sullivan , “Cryopreservation of Brain Mitochondria: A Novel Methodology for Functional Studies,” Journal of Neuroscience Methods 152, no. 1‐2 (2006): 48–54. 16246427 10.1016/j.jneumeth.2005.08.017

[400] Y. Yamada , M. Ito , M. Arai , et al., “Challenges in Promoting Mitochondrial Transplantation Therapy,” International Journal of Molecular Sciences 21, no. 17 (2020): 6365. 32887310 10.3390/ijms21176365 PMC7504154

[401] R. Yamaguchi , A. Andreyev , A. N. Murphy , et al., “Mitochondria Frozen With Trehalose Retain a Number of Biological Functions and Preserve Outer Membrane Integrity,” Cell Death and Differentiation 14, no. 3 (2007): 616–624. 16977331 10.1038/sj.cdd.4402035

[402] M. D'Amato , F. Morra , I. Di Meo , and V. Tiranti , “Mitochondrial Transplantation in Mitochondrial Medicine: Current Challenges and Future Perspectives,” International Journal of Molecular Sciences 24, no. 3 (2023): 1969. 36768312 10.3390/ijms24031969 PMC9916997

[403] T. G. Zhang and C. Y. Miao , “Mitochondrial Transplantation as a Promising Therapy for Mitochondrial Diseases,” Acta Pharmaceutica Sinica B 13, no. 3 (2023): 1028–1035. 36970208 10.1016/j.apsb.2022.10.008 PMC10031255

[404] N. Borcherding , W. Jia , R. Giwa , et al., “Dietary Lipids Inhibit Mitochondria Transfer to Macrophages to Divert Adipocyte‐derived Mitochondria Into the Blood,” Cell metabolism 34, no. 10 (2022): 1499–1513. e8. 36070756 10.1016/j.cmet.2022.08.010 PMC9547954

[405] J. R. Brestoff , C. B. Wilen , J. R. Moley , et al., “Intercellular Mitochondria Transfer to Macrophages Regulates White Adipose Tissue Homeostasis and Is Impaired in Obesity,” Cell metabolism 33, no. 2 (2021): 270–282. e8. 33278339 10.1016/j.cmet.2020.11.008 PMC7858234

[406] J. Xie , Z. Shen , Y. Anraku , et al., “Nanomaterial‐based Blood‐brain‐barrier (BBB) Crossing Strategies,” Biomaterials 224 (2019): 119491. 31546096 10.1016/j.biomaterials.2019.119491 PMC6915305

[407] A. Aleynik , K. M. Gernavage , Y. S. Mourad , et al., “Stem Cell Delivery of Therapies for Brain Disorders,” Clinical and translational medicine 3 (2014): 24. 25097727 10.1186/2001-1326-3-24 PMC4106911

[408] G. Ramirez‐Barbieri , K. Moskowitzova , B. Shin , et al., “Alloreactivity and Allorecognition of Syngeneic and Allogeneic Mitochondria,” Mitochondrion 46 (2019): 103–115. 29588218 10.1016/j.mito.2018.03.002

[409] J. Pollara , R. W. Edwards , L. Lin , et al., “Circulating Mitochondria in Deceased Organ Donors Are Associated With Immune Activation and Early Allograft Dysfunction,” JCI Insight 3, no. 15 (2018): e121622. 30089724 10.1172/jci.insight.121622 PMC6129133

[410] Q. Zhang , M. Raoof , Y. Chen , et al., “Circulating Mitochondrial DAMPs Cause Inflammatory Responses to Injury,” Nature 464, no. 7285 (2010): 104–107. 20203610 10.1038/nature08780 PMC2843437

[411] L. Lin , H. Xu , M. Bishawi , et al., “Circulating Mitochondria in Organ Donors Promote Allograft Rejection,” Am J Transplant Off J Am Soc Transplant Am Soc Transpl Surg 19, no. 7 (2019): 1917–1929. 10.1111/ajt.15309 PMC6591073 30761731

[412] J. Burrello , S. Monticone , C. Gai , et al., “Stem Cell‐Derived Extracellular Vesicles and Immune‐Modulation,” Frontiers in Cell and Developmental Biology 4 (2016): 83. 27597941 10.3389/fcell.2016.00083 PMC4992732

[413] K. Reinhardt , D. K. Dowling , and E. H. Morrow , “Medicine. Mitochondrial Replacement, Evolution, and the Clinic,” Science 341, no. 6152 (2013): 1345–1346. 24052294 10.1126/science.1237146

[414] R. Dobler , D. K. Dowling , E. H. Morrow , and K. Reinhardt , “A Systematic Review and Meta‐analysis Reveals Pervasive Effects of Germline Mitochondrial Replacement on Components of Health,” Human Reproduction Update 24, no. 5 (2018): 519–534. 29757366 10.1093/humupd/dmy018

[415] A. Eyre‐Walker , “Mitochondrial Replacement Therapy: Are Mito‐nuclear Interactions Likely To Be a Problem?,” Genetics 205, no. 4 (2017): 1365–1372. 28360127 10.1534/genetics.116.196436 PMC5378100

[416] R. Gupta , M. Kanai , T. J. Durham , et al., “Nuclear Genetic Control of mtDNA Copy Number and Heteroplasmy in Humans,” Nature 620, no. 7975 (2023): 839–848. 37587338 10.1038/s41586-023-06426-5 PMC10447254

[417] M. S. Sharpley , C. Marciniak , K. Eckel‐Mahan , et al., “Heteroplasmy of Mouse mtDNA Is Genetically Unstable and Results in Altered Behavior and Cognition,” Cell 151, no. 2 (2012): 333–343. 23063123 10.1016/j.cell.2012.09.004 PMC4175720

[418] D. B. Cowan , R. Yao , J. K. Thedsanamoorthy , et al., “Transit and Integration of Extracellular Mitochondria in human Heart Cells,” Scientific Reports 7, no. 1 (2017): 17450. 29234096 10.1038/s41598-017-17813-0 PMC5727261

[419] A. G. Cox , C. C. Winterbourn , and M. B. Hampton , “Mitochondrial Peroxiredoxin Involvement in Antioxidant Defence and Redox Signalling,” Biochemical Journal 425, no. 2 (2010): 313–325. 10.1042/BJ20091541 20025614

